# Synthetic and structure–activity studies of SP2577 and TCP towards LSD1 targeting PROTACs†

**DOI:** 10.1039/d5md00420a

**Published:** 2025-08-05

**Authors:** Megan E. Coulson, James K. S. Norris, Sean A. Smith, Joshua P. Smalley, John W. R. Schwabe, Shaun M. Cowley, James T. Hodgkinson

**Affiliations:** a Leicester Institute of Structural and Chemical Biology and School of Chemistry, University of Leicester University Road Leicester LE1 7RH UK JTHodgkinson@le.ac.uk; b Department of Molecular and Cell Biology, University of Leicester Leicester LE1 9HN UK smc57@leicester.ac.uk; c Leicester Institute of Structural and Chemical Biology and Department of Molecular and Cell Biology, University of Leicester Leicester LE1 9HN UK john.schwabe@le.ac.uk

## Abstract

Lysine-specific histone demethylase 1A (LSD1) is involved in epigenetic regulation and is a viable drug target with a number of LSD1 inhibitors in clinical trials. We report synthetic and structure–activity studies of two LSD1 inhibitors, TCP and SP2577, in clinical trials towards PROTAC development. 16 Heterobifunctional molecules were synthesised based on TCP and SP2577. No LSD1 degraders were identified in HCT116 cells, however two TCP analogues functionalised from the phenyl ring with an aklyl and PEG linker in combination with a VHL ligand demonstrated potent LSD1 inhibition *in vitro* in the HDAC1-CoREST-LSD1 complex (43 nM and 63 nM respectively). Our findings provide important SAR data towards LSD1 PROTACs.

## Abstract 2

An SAR study of SP2509/SP2577 and TCP-based PROTACs targeting LSD1 revealed potent *in vitro* inhibitors, but no LSD1 degradation was observed in HCT116 cells.

## Graphical abstract

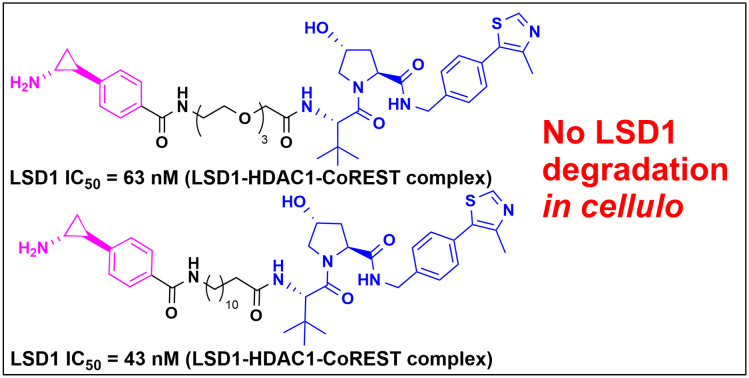

## Introduction

Lysine-specific histone demethylase 1A (LSD1) also abbreviated as KDM1A is a flavin dependent monoamine oxidase that exists *in vivo* as a tripartite complex with HDAC1/2 and CoREST. As part of the CoREST complex LSD1 interacts with nucleosomes and catalyses the demethylation of methylated lysine histone residues including mono or di-methylated histone H3 lysine 4 (H3K4me1/2) and histone H3 lysine 9 (H3K9me1/2).^1–3^ Hence, LSD1 in the CoREST complex influences chromatin structure, DNA accessibility and gene expression and is considered an epigenetic regulator.^4^

**Fig. 1:**
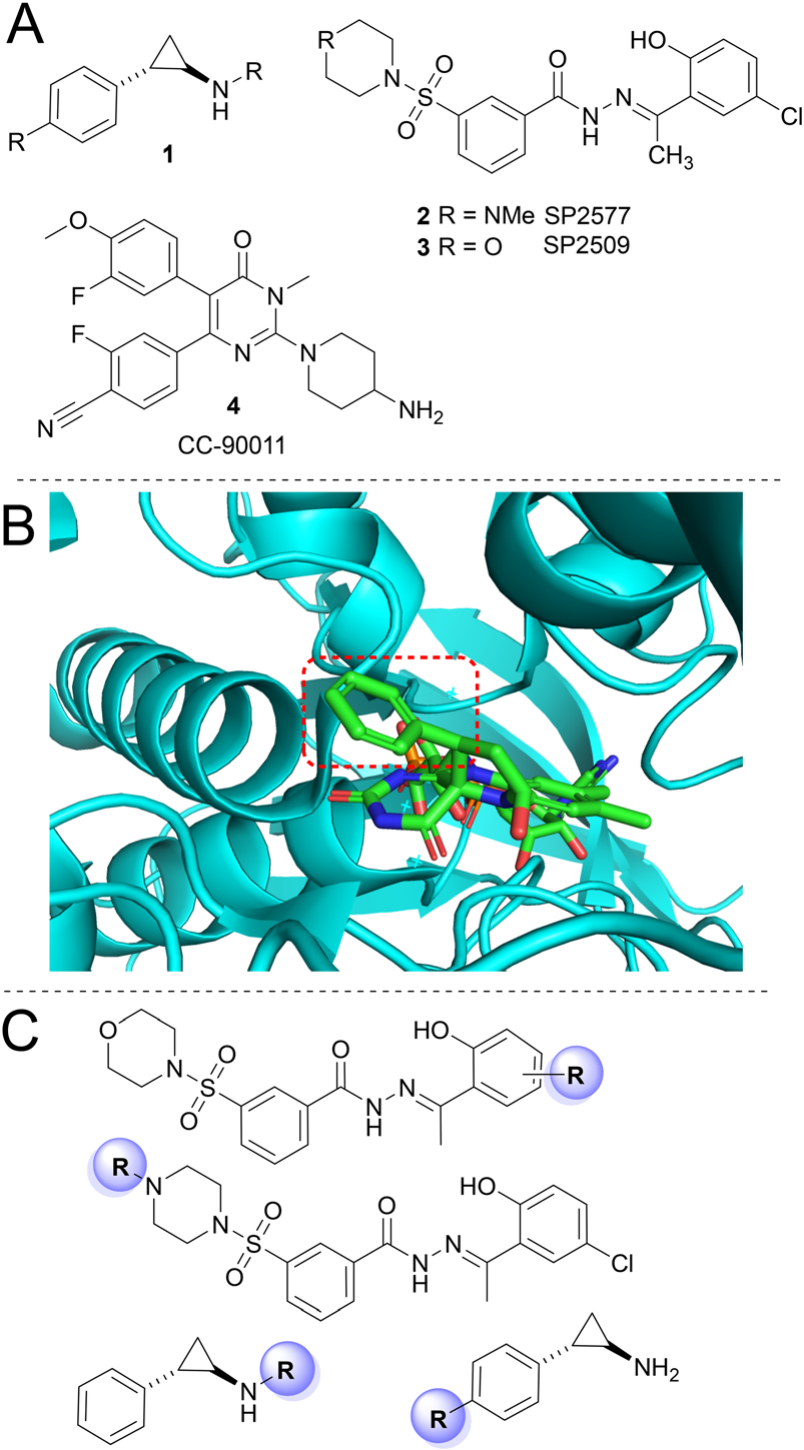
A) Structural scaffolds of LSD1 inhibitors currently in clinical trials. B) LSD1 crystal structure with TCP covalently bound to the cofactor FAD (cyclopropane ring cleaved), the phenyl ring of TCP is highlighted in the dashed red box (PDB: 2uxx). C) Structure activity studies of SP257/2509 and TCP reported in this study towards LSD1 PROTACs. R groups represent positions of functionalisation for linker attachment for PROTAC development.

A number of LSD1 inhibitors have entered clinical trials (Fig. 1A).^5^ The large majority of these inhibitors incorporate the monoamine oxidase inhibitor tranylcypromine (TCP), 1, as a core scaffold (Fig. 1A). TCP can bind to LSD1 *via* covalent bonds to the cofactor flavin adenine dinucleotide (FAD) in the active site of LSD1 (Fig. 1B),^6^ with the cyclopropane ring of TCP no longer intact. TCP itself has relatively low affinity for LSD1,^7^ however modified TCP analogues can exhibit high affinity and selectivity for LSD1.^7,8^ Examples of such LSD1 inhibitors include ORY1001, ORY2001, GSK-2879552, INCB059872, JBI-802, TAK-418 and LH-1802 that are in clinical trials for a range of diseases including acute myeloid leukaemia (AML), solid tumours, neurological disorders and multiple sclerosis.^5^

SP2577, 2, originally derived from SP2509, 3, is another LSD1 inhibitor in clinical trials for Ewing sarcoma (Fig. 1A).^9,10^ SP2509 and SP2577 are reported to be reversible non-competitive inhibitors of LSD1, differing to the covalent LSD1 inhibition of TCP. It has previously been reported that SP2509 inhibits LSD1 by an allosteric mechanism of action, interfering with LSD1 protein–protein interactions such as the CoREST-LSD1 interaction.^11–13^ However, counter to these studies others have isolated full FLAG tagged CoREST-LSD1-HDAC1 complex from insect cells during treatment with SP2509.^14^ No structural data of SP2509 or SP2577 bound to LSD1 or components of the CoREST complex have been reported to date. CC-90011, 4, is a reversible inhibitor of LSD1 and has been in clinical trials for AML and solid tumours (Fig. 1A). Structural data show CC-90011 binds reversibly to the LSD1 catalytic active site, in relatively close proximity to the FAD co-factor pocket.^15^

Proteolysis targeting chimeras (PROTACs) are heterobifunctional molecules that engage a protein of interest (POI) and an E3-ligase to recruit the ubiquitin-proteasome system to degrade the POI and remove it from the cell. PROTACs and other targeted protein degradation approaches are receiving significant investment from the pharmaceutical industry as novel strategies in drug discovery.^16^ There are many comprehensive reviews available on the PROTAC field and their potential advantages and challenges in drug discovery.^17–20^

Epigenetic drug targets are of particular interest and significance in PROTAC discovery.^21^ Probably the most frequently explored POI in the PROTAC field is bromodomain-containing protein 4 (BRD4).^22^ Other notable epigenetic PROTAC targets include histone acetyl transferases (HATs),^23^ histone deacetylases (HDACs)^24^ and polycomb-group proteins (PcG).^25^ The Jumonji C (JmjC) domain-containing lysine demethylases KDM4 and KDM5B have been successfully targeted with PROTACs.^26,27^ The mode of action of a molecular glue, UM171, that degrades HDAC1/2-CoREST and subsequently LSD1 in cells has been reported.^28^ PROTACs targeting HDAC1/2 for degradation also have the capacity to reduce LSD1 abundance.^29^

Recently, a PROTAC based on CC-90011 for the targeted degradation of LSD1 was reported by Hosseini *et al.*^30^ To date, this is the only PROTAC reported for LSD1. PROTACs that directly engage with LSD1 to target its degradation may have advantages in avoiding targeting other HDAC1/2 containing corepressor complexes, as it would be expected to exclusively target the HDAC1/2-CoREST-LSD1 complex that LSD1 resides in.

In comparison to the study by Hosseini *et al.*, focusing on CC-90011, we set about synthesising LSD1 targeting PROTACs using the covalent LSD1 inhibitor (TCP) and reversible inhibitor SP2577/SP2509. Our investigations included building off multiple positions on both inhibitors to evaluate the optimal site for PROTAC development (Fig. 1C). In total 16 heterobifunctional molecules were synthesised and assessed for their ability to inhibit LSD1 *in vitro* and degrade LSD1 in cells. Two heterobifunctional TCP analogues functionalised with an alkyl and PEG liker in combination with the VHL E3-ligand demonstrated potent LSD1 inhibition *in vitro*. However, despite this, no LSD1 degraders were identified in HCT-116 cells.

## Results and discussion

### Synthetic and structure–activity studies of SP2509 and SP2577

**Fig. 2:**
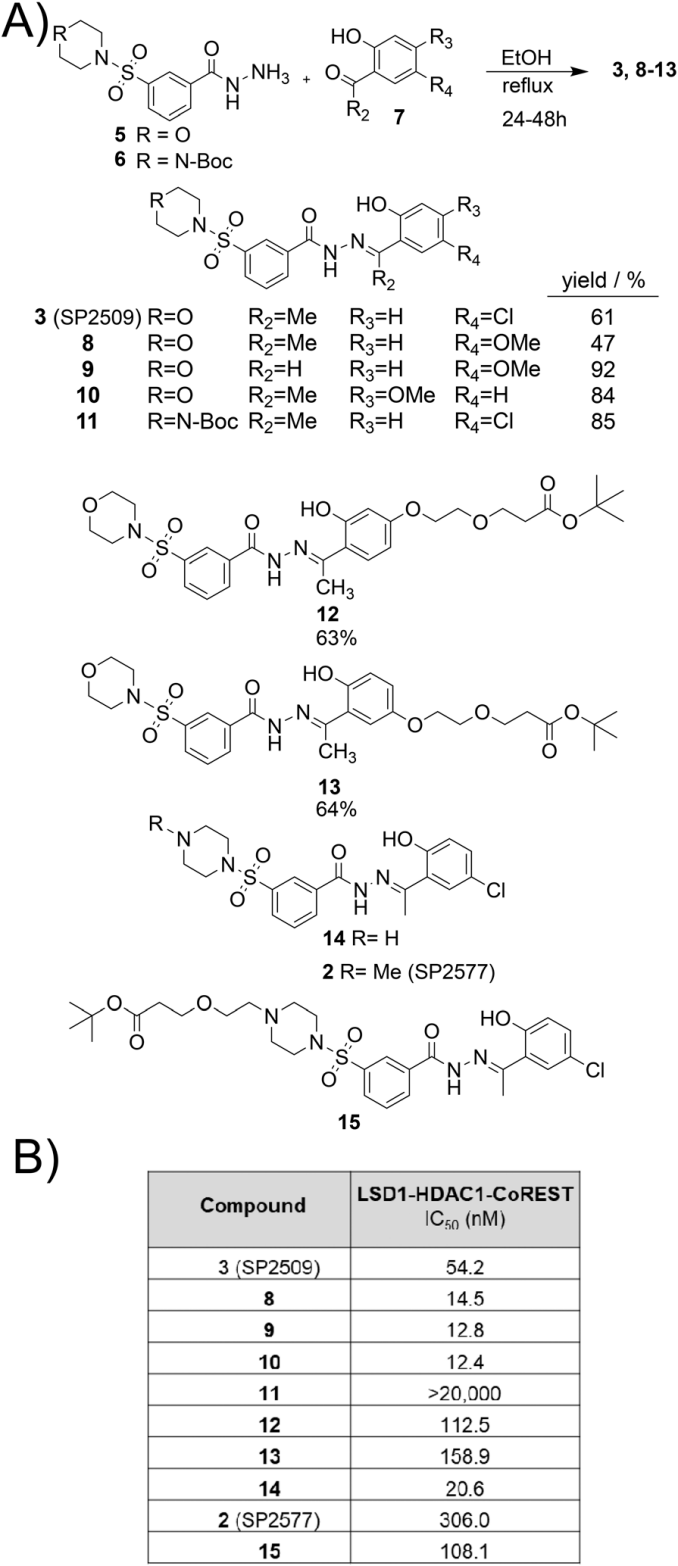
A) SP2509 and SP2577 analogues synthesised B) IC_50_ values for LSD1 enzymatic activity in the LSD1-HDAC1-CoREST complex (50 nM). IC_50_ values for compounds 8, 9, and 10 were determined in parallel with SP2509, as were those for compounds 11, 2, and 14, and separately for 13, 12, and 15. Each set included SP-2509 as a reference standard. The mean IC_50_ value for SP2509 was 54.2 nM ± 2.0 nM. Full assay conditions and protocols are available in the ESI.†

No crystal structure of SP2509 or SP2577 bound to LSD1 have been reported. Based on this, we synthesised a series of analogues to explore alternative linker attachment sites for PROTAC development. We hypothesised that the chlorine atom present in SP2509 and SP2577 could be substituted for a methoxy group (Fig. 1C), a known bioisostere for Cl, and if tolerated derivatised into a linker. Additionally, we rationalised that the nitrogen atom in SP2577 could also be a potential position for linker attachment (Fig. 1C). Hydrazides 5 and 6 (Fig. 2A) were prepared in 3 steps (see ESI†). 5 and 6 were then reacted with commercially available substituted acetophenone analogues 7 (Fig. 2A) to yield SP2509 and SP2577 analogues 8–13. The same reaction was carried out for the linker functionalised analogues of SP2509, 12 and 13 (Fig. 2A), although the corresponding acetophenones were synthesised (see ESI†).

To prepare 2 (SP2577) and analogues 14 and 15, we initially attempted to remove the Boc group of 11 using 1 : 1 TFA/DCM over 18 hours, however these conditions also completely cleaved the hydrazone. With 9% TFA in DCM and a short reaction time (1 hour) the Boc group could be removed while maintaining the hydrazone to give 14. Reductive amination with formaldehyde and sodium cyanoborohydride gave 2 (SP2577). We again used reductive amination to prepare linker functionalised SP2577 analogue 15 (see ESI† for full synthesis details). The purification of 15 was initially attempted by semi-preparative HPLC, however we found TFA present in the buffer also cleaved the hydrazone bond, and TFA had to be excluded from buffers for purification.

The analogues synthesised were then tested for their ability to inhibit LSD1 using purified LSD1-HDAC1-CoREST complex (Fig. 2B). We used an established fluorescent read out horseradish peroxidase (HRP) assay to monitor LSD1 enzymatic activity in the CoREST complex^8^ (see ESI† for full details). SP2509 had an IC_50_ = 54.2 nM, this is comparable with previous literature whereby SP2509 was reported to inhibit LSD1 with an IC_50_ = 13 nm (evaluated not part of the CoREST complex).^9^ SP2577 exhibited an IC_50_ = 0.306 μM, there are few reports for the IC_50_ value of SP2577 against LSD1. The original patent including SP2577 reported a mean IC_50_ value of 0.127 μM,^31^ and a recent study by Sacilotto *et al.* reported a IC_50_ = 1.33 μM,^8^ our result is within this range. It was pleasing to observe that substituting the Cl atom in SP2509 with an methoxy group in 8 resulted in more potent LSD1 inhibition in this assay compared to SP2509 (Fig. 2B).

In fact, the methoxy group was tolerated on a differing position on the phenyl ring in 10 (Fig. 2B). Additionally, the presence of a methyl group or hydrogen atom in the R2 position in 8 or 9 did not affect LSD1 inhibition, with analogues 8–10 near equipotent (Fig. 2B). With the linker functionalised SP2509 analogues 12 and 13, there was an increase in the IC_50_ value to 113 nM and 159 nM respectively, however we were pleased with this result given 13 and 14 were only 2–3 fold less potent than SP2509 and still retained sub-micromolar LSD1 inhibition. Intriguingly, regards the SP2577 analogues, analogue 14 without the methyl substitution on the piperazine had a potent IC_50_ value of 20.6 nM, introduction of the linker in 15 increased that IC_50_ value to 108 nM and addition of the Boc group in 11 increased the IC_50_ value to greater than 20 μM.

**Scheme 1:**
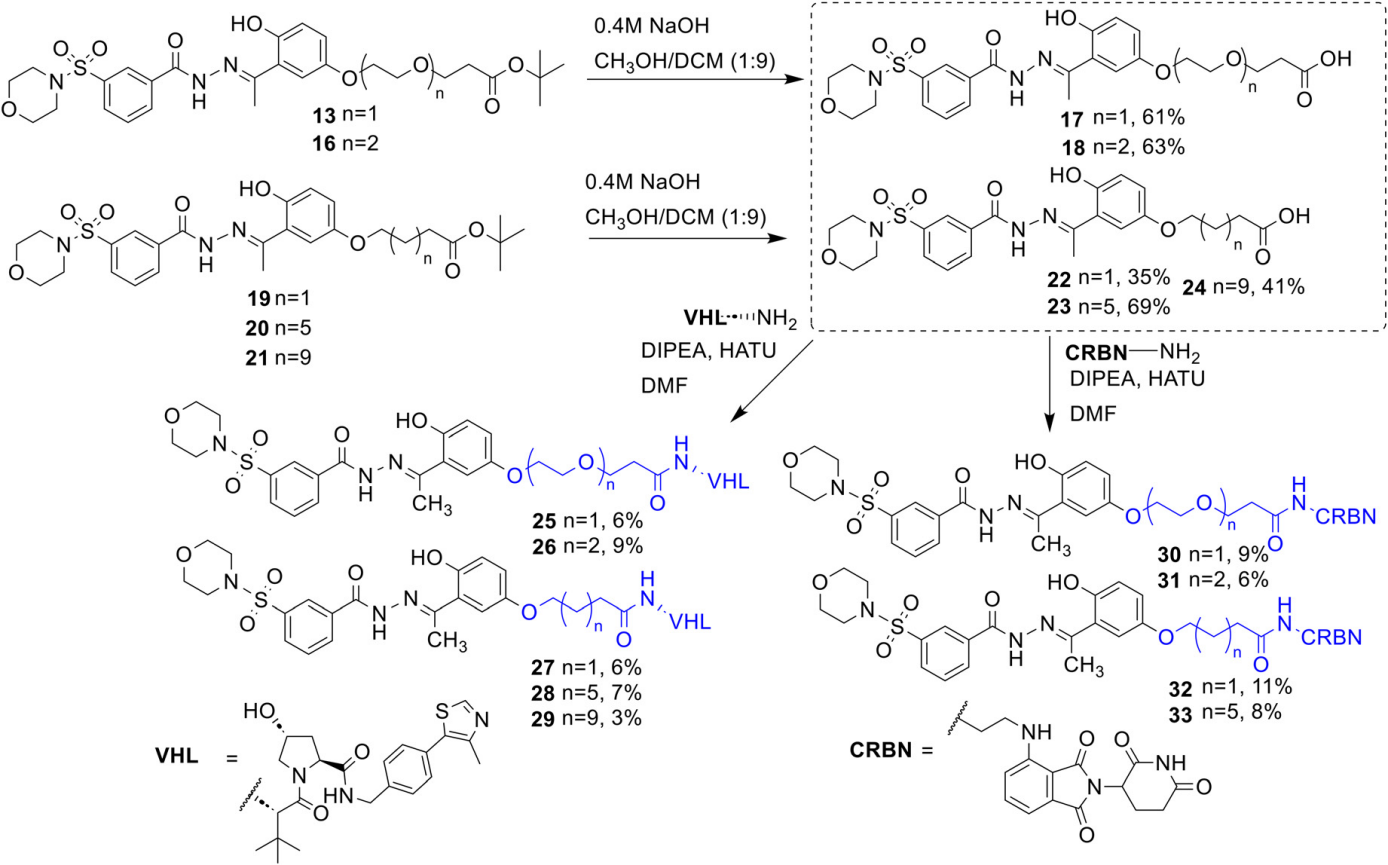
Synthetic routes to SP2509 heterobifunctional analogues with cereblon and VHL E3 ligands incorporated.

Having determined that functionalisation of analogues 12, 13 and 15, with a linker maintained sub-micromolar inhibition of LSD1 with similar IC_50_ values, based on synthetic tractability we decided to pursue 13 for PROTAC development. Four more analogues of 13 were prepared (Scheme 1). Analogue 16 is one PEG unit longer that 13, and analogues 19, 20, and 21 vary in alkyl linker length (for full synthesis see ESI†). To remove the *tert*-butyl group to make analogues 17, 18, 22 and 23, based on our previous results on the acid lability of the hydrazone bond, we employed basic conditions in NaOH, MeOH and DCM to remove the *tert*-butyl group (Scheme 1). HATU amide coupling reaction conditions were employed with 17, 18, 22 and 23, and VHL ligand to make the VHL heterobifunctional molecules 25–29. The same conditions were employed to prepare the cereblon heterobifunctional molecules 30–33, the reaction between 24 and the cereblon ligand was attempted however failed. Yields for these reactions were exceptionally poor, a side product observed was again cleavage of the hydrazone bond which seemed to be dependent upon the equivalents of HATU employed. We suspect that the nucleophilicity of 1-hydroxy-7-azabenzotriazole generated in the HATU reaction may react with the labile hydrazone. The purifications of these compounds were also exceptionally challenging due to multiple side-products that co-eluted close to the desired products. However, despite these challenges we were able to obtain analytically pure 25–33 (≥95% by analytical HPLC). ^1^H NMR analysis of 25–33 was also consistent with this.

We next screened 25–33 and SP2509 over a concentration range of 0.1–10 μM in HCT116 cells (obtained from ATCC, CCL-247) and determined LSD1 abundance after 24 hours with an antibody for LSD1 (Fig. S4†). Disappointingly, no degradation or change in LSD1 levels were observed. Using the same conditions, we also determined the effects of SP2509, 25, 27, 28, 30 and 31 on histone H3 lysine 4 dimethyl (H3K4me2) a validated substrate for LSD1 using a specific antibody for H3K4me2 (Fig. S4†). Again, disappointingly, we observed no increase in H3K4me2 with SP2509 or 25, 27, 28, 30 and 31. We were particularly surprised that SP2509 did not increase H3K4me2 abundance. However, in accordance with these results, a recent study by Senanayaka *et al.*^32^ specifically assessed the ability of LSD1 inhibitors TCP, SP2577 and CC-9001 to increase H3K4me2 on peptides and nucleosomes, whereby SP2577 had no effect. TCP and CC-9001 did result in increased H3K4me2 levels as expected, suggesting SP2577 is not acting as a LSD1 inhibitor, or at least an inhibitor of the enzymatic active site of LSD1. Additionally, in their study only SP2577 failed to increase H3K4me2 levels in cells.

The authors suggest that SP2577 may be specifically interfering with the LSD1 HRP-coupled inhibition assay, and for SP2577 and related analogues this assay may be misleading in terms of LSD1 enzymatic inhibition evaluation. This is something we cannot rule out in our study. We also observed significant cytotoxic effects with SP2509 in cells that were not observed with the much more potent TCP based LSD1 inhibitor ORY1001 (IC_50_ = 2 nM) in clinical trials (Fig. S3†), suggesting potential off-targets. Overall, we were discouraged by these results, in terms of any potential future PROTAC drug development. Based on this, we next turned our attention to TCP analogues for SAR studies and potential PROTAC development.

### Synthetic and structure–activity studies of TCP

As a crystal structure of TCP bound to the cofactor FAD in the LSD1 active site is available, we decided to use it to inform PROTAC design.^6^ Based on this, functionalising from the phenyl ring of TCP should perturb the linker and E3-ligand into solvent exposed space for potential interactions with an E3 ligase (Fig. 1B and C). We also decided to functionalise the amine position of TCP (Fig. 1C), a number of potent LSD inhibitors including ORY1001 and GSK-2879552 and many others have been functionalised on this position.^7,8,33,34^

**Scheme 2:**
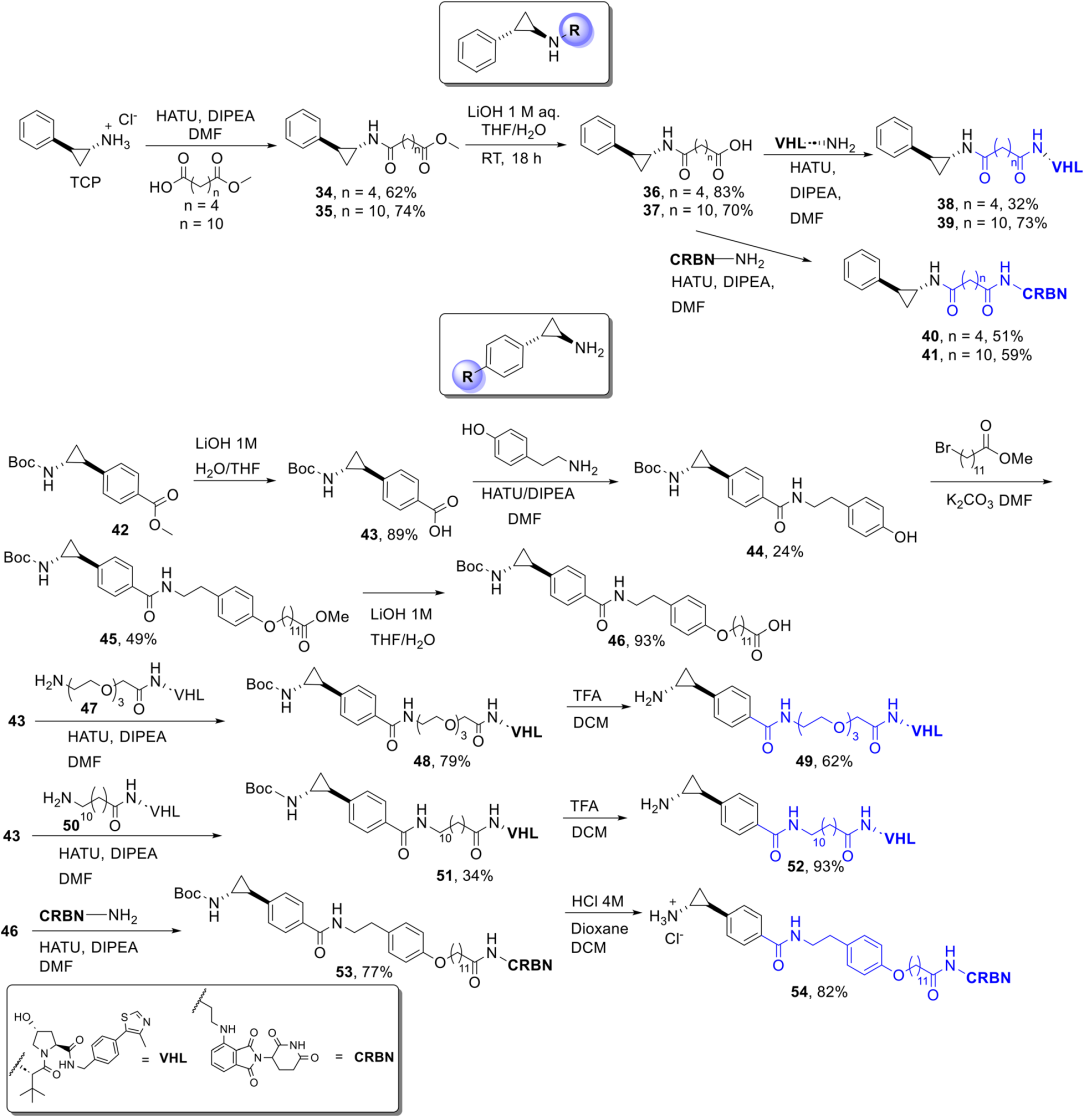
Synthetic routes to TCP heterobifunctional analogues with cereblon and VHL E3 ligands incorporated.

To synthesise the TCP analogues functionalised from the amide position we utilised a similar synthetic route we previously reported for HDAC targeting PROTACs.^35,36^ Racemic TCP hydrochloride salt was reacted with mono-methyl adipate and 12-(methyloxy)-12-oxododecanoic acid using HATU amide coupling conditions to yield 34 and 35 (Scheme 2). These were subsequently hydrolysed to give acids 36 and 37. 36 and 37 were coupled to VHL and Cereblon E3 ligands to give 38–41 in modest to good yields.

Functionalisation from the phenyl ring of TCP required more synthetic effort. 42 was prepared in four steps as previously reported by Borrello *et al.*^37^ (ESI† for full synthesis). 43 was reacted with commercially available VHL functionalised PEG linker, 47, *via* HATU coupling conditions to give intermediate 48. The Boc group was removed under standard conditions in TFA/DCM to give the TCP analogue 49 functionalised from the phenyl ring with a PEG linker and VHL. 43 was also reacted in the same HATU coupling conditions with a VHL ligand functionalised with a 12-carbon linker 50, which was synthesised (see ESI† for full synthesis). Removal of the Boc group in TFA/DCM yielded the TCP functionalised analogue from the phenyl ring with a 12-carbon alkyl linker and VHL ligand 52. Compound 44 was inspired by Borrello *et al.*,^37^ we introduced the hydroxyl group in 44 as a position for linker attachment for potential PROTACs. 44 was reacted with methyl 12-bromododecanoate to give 45 followed by hydrolysis of the methyl ester to give 46. HATU amide coupling conditions were employed between 46 and the CRBN E3-ligand to give 53. The Boc group was removed using HCl in dioxane/DCM to give 54.

We did attempt the synthesis of the VHL analogue of 54 from 53, which was successfully identified in NMR of the crude reaction mixture, however, on the purification of this VHL analogue the cyclopropane ring opened.

**Fig. 3:**
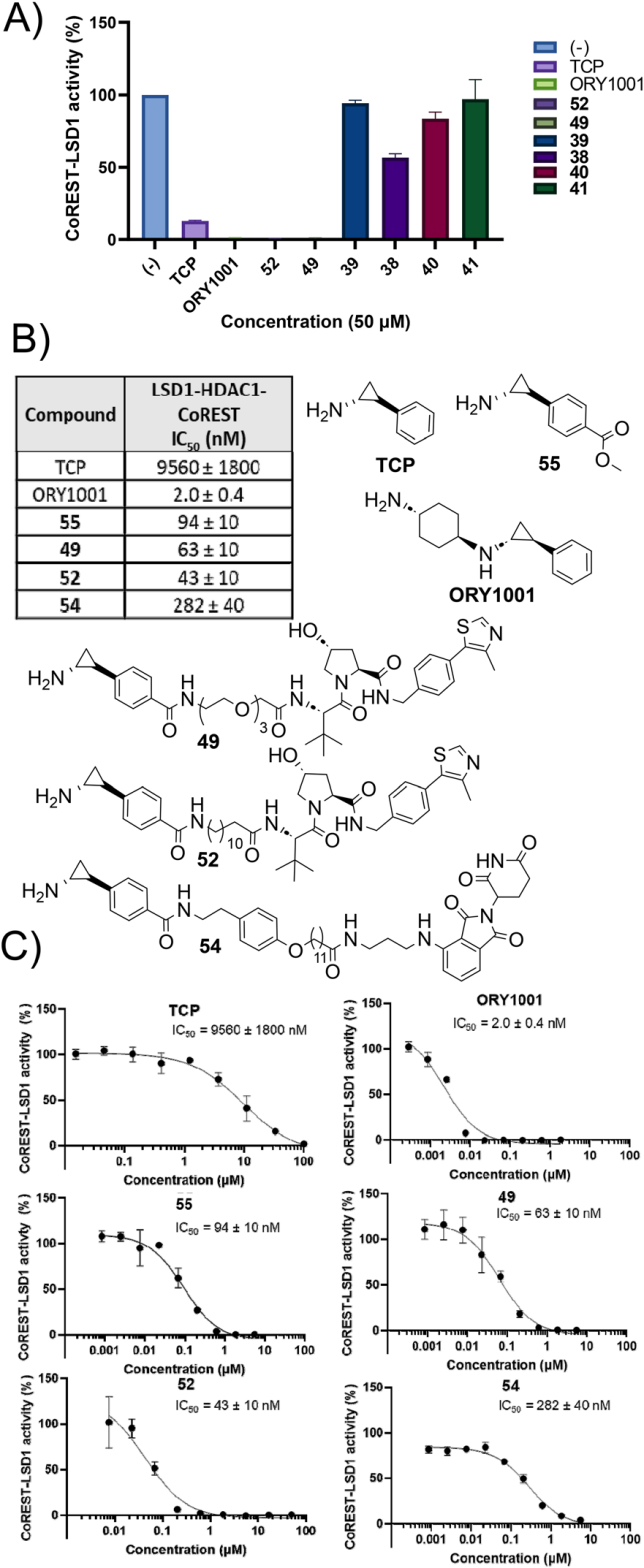
A) LSD1 inhibition in the HDAC1-CoREST-LSD1 complex (50 nM) of selected compounds at 50 μM. B) Structures of compounds tested for dose-responsive LSD1 inhibition and their IC_50_ values. C) Dose response curves for LSD1 inhibition in the HDAC1-CoREST-LSD1 (50 nM). IC_50_ values are expressed as mean ± SD from three replicates. Full assay conditions and protocols are available in the ESI.†

We then evaluated LSD1 inhibition in the LSD1-HDAC1-CoREST complex using the established horseradish peroxidase (HRP) assay.^8^ We initially tested TCP, ORY1001 (in clinical trials) and selected compounds 49, 52 and 39–41, each at a single concentration of 50 μM, to determine which compounds to further evaluate for IC_50_ determination. TCP inhibited LSD1 activity at 50 μM with approx. 88% inhibition compared to no compound treatment control (Fig. 3A). ORY1001, 52 and 49 inhibited LSD1 enzymatic activity 100% at this concentration. 39, 40 and 41 were not LSD1 inhibitors at 50 μM, although 38 demonstrated modest inhibition at this high concentration (Fig. 3A). Clearly, based on these results, 39–41 functionalised from the amine of TCP, compromised LSD1 inhibition. Interestingly, ORY1001 is also functionalised from the amine of TCP yet maintains LSD1 inhibition as do other LSD1 inhibitors functionalised from this position in clinical trials. One significant difference between 39–41 and inhibitors such as ORY1001 in clinical trials is the presence of the amide bond in 39–41. Based on this, we hypothesise that the lone pair on the nitrogen atom of the amide bond is not as available for the single electron reduction of FAD that has been proposed as the first step in LSD1 inhibition by TCP.^6^

Given 52 and 49, functionalised from the phenyl ring of TCP, maintained LSD1 inhibition, we determined the IC_50_ values of 49, 52, 54 and 55 (Fig. 1B and C), all of which are functionalised from the phenyl ring position. We also determined the IC_50_ values of TCP and ORY1001 for direct comparison. TCP exhibited an IC_50_ value of 9.56 μM, this is comparable to previous literature (IC_50_ = 5.63 μM, LSD1 only).^8^ ORY1001 exhibited an IC_50_ value of 2 nM, again comparable to previously reported literature values (IC_50_ = 18 nM and 0.3 nM).^8,33^ We were astonished that the introduction of the methyl ester on the phenyl ring of TCP, 55, reduced the IC_50_ value to 94 nM. This is an approximately 100-fold enhancement in LSD1 inhibition compared to TCP with such a small modification. 55 has previously been reported in two patents related to LSD1 theraputics.^38,39^ Pleasingly, this LSD1 inhibition was maintained with 49 (IC_50_ = 63 nM) and 52 (IC_50_ = 43 nM). Analogue 54 based on the LSD1 inhibitor developed by Borrello *et al.*^37^ with an alkyl linker and cereblon E3 ligand exhibited an IC_50_ value of 0.28 μM. While higher than 49 and 52, the IC_50_ value of 54 is comparable to the parental inhibitor from which it was originally derived (IC_50_ = 0.4 μM).^37^ Clearly these results demonstrate that functionalising TCP from the phenyl ring with linkers and E3-ligands does not interfere with LSD1 inhibition, and that such analogues are suitable for PROTAC development in at least with regards to not interfering with LSD1 engagement within the LSD1-HDAC1/2-CoREST complex.

Compounds 39–41, 49, 52 and 54 were screened for LSD1 degradation in HCT116 cells using a specific antibody for LSD1. This was performed side-by-side with TCP. Compounds were tested at concentration ranges of 0.2–20 μM and LSD1 abundance was quantified at 24 hours (Fig. S5†). 49, 52 and 54 were also evaluated at 48 and 72 hours. Disappointingly, no degradation of LSD1 was observed. We also determined the effects of these compounds on the histone methyl marker H3K4me2 (Fig. S5 and S6†). Again, we saw no changes in these histone makers, surprisingly neither did we observe changes with these histone markers in the presence of TCP or ORY1001.

## Conclusions

16 Heterobifunctional molecules were synthesised based on SP2577 and TCP, two LSD1 inhibitors in clinical trials. These heterobifunctional molecules encompassed various linkers in combination with the VHL or CRBN E3-liagnd. We identified three linker attachment positions to SP2509/SP2577 in 12, 13 and 15 that maintained sub-micromolar LSD1 inhibition *in vitro*. Analogue 13 was selected for further derivatization into nine potential PROTACs; however, no LSD1 degraders were identified in HCT116 cells. Dulmi Senanayaka *et al.* recently proposed that SP2577 interferes with the LSD1 HRP assay itself (rather LSD1 enzymatic inhibition) leading to false positives regards LSD1 inhibition in this assay.^32^ Additionally, we observed cytotoxic effects in HCT116 cells with SP2509 that was not observed with TCP and the more potent TCP based LSD1 inhibitor ORY1001 (Fig. S3†), thus suggesting potential off-targets related to SP2509 which others have also reported.^40^ During our synthetic studies of these analogues, we observed that the hydrazone bond was particularly labile, which may have also influenced the results observed in in cells.

Given these results, any potential further development of SP2577/SP2509 into LSD1 targeting PROTACs will have to overcome significant challenges.

Regards TCP analogues for potential PROTAC development, 49 and 52, functionalised from the phenyl ring of TCP demonstrated potent LSD1 inhibition of the LSD1-HDAC1-CoREST complex *in vitro* (63 nM and 43 nM respectively). Hence, at least with regards to maintaining LSD1 binding, such analogues are suitable for further PROTAC development. Disappointingly, neither 49 or 52 or any of the TCP based heterobifunctional molecules reduced LSD1 abundance in HCT116 cells. Surprisingly, TCP and ORY1001 also did not increase H3K4me2 levels either. Additionally, similar to any PROTAC study, we cannot rule out that an optimum linker length/composition or E3 ligand combination analogous to 49 or 52 would yet lead to LSD1 degraders. Hence, additional exploration of TCP from the phenyl position may warrant further studies towards LSD1 targeting PROTACs.

Although covalent PROTACs for various targets have been reported, there are examples whereby irreversible covalent PROTACs, such that would be expected for TCP based PROTACs reported here, have not led to degradation compared to reversible PROTACs.^41^ In this regard CC-90011 is a reversible LSD1 inhibitor with a crystal structure of CC-90011 bound to LSD1 to guide PROTAC design. During the preparation of this manuscript a LSD1 targeting PROTAC was reported based on CC-90011.^30^ Although this study focused exclusively on the CRBN E3 ligand, the linkers explored in this study do not differ greatly from ours. Including the shorter PEG linkers that led to the active LSD1 degraders based on CC-90011. This perhaps further highlights the importance of the choice of ligand for the protein of interest in PROTAC design.

## Author contributions

JTH, SMC and JWRS conceived the project and oversaw supervision of different aspects of the project. JTH and SAS wrote and prepared the manuscript. MEC was the main experimentalist regards TCP aspects of the manuscript and JKSN regards SP2509/SP2577 aspects of the manuscript. JPS synthesised the Cereblon E3-ligand used in the study and linker intermediates.

## Conflicts of interest

There are no conflicts to declare.

## Supplementary Material

MD-OLF-D5MD00420A-s001

## Data Availability

The data supporting this article have been included as part of the ESI.†
