# Supplementary material for: Synthetic and structure–activity studies of SP2577 and TCP towards LSD1 targeting PROTACs
Source: RSC Med Chem. 2025 Aug 5;16(10):4952–9. doi: 10.1039/d5md00420a (PMC12323867; doi:10.1039/d5md00420a)
Supplement: MD-016-D5MD00420A-s001 [file MD-016-D5MD00420A-s001.pdf]

Supplementary Information (SI) for RSC Medicinal Chemistry.
This journal is © The Royal Society of Chemistry 2025

Synthetic and structure-activity studies of SP2577 and TCP towards LSD1 targeting PROTACs

Megan E. Coulson,[a,b†] James K.S. Norris,[a†] Sean A. Smith,[a] Joshua P. Smalley[a] , John W.R. Schwabe,[c*] Shaun M. Cowley[b*] and James T. Hodgkinson[a]*

Supplementary Information

Summary

1. Supplementary Information: Chemistry 3

1.1 General Information 3

1.1.1. Synthesis of Cereblon-based amine **CRBN-NH$_2$** 4

1.2. Synthesis of SP2509 and SP2577 related compounds and PROTACs 7

1.2.1. Synthesis of precursor hydrazides **5-6** 7

1.2.2. Synthesis of hydrazones **3** (SP2509) and **8-11** 13

1.2.3. Synthesis of acetophenone precursors to **12** and **13** 17

1.2.4. Synthesis of hydrazones **2** (SP2577) and **11-15** 20

1.2.5. Preparation of compounds **16**, **19**, **20** and **21** 24

1.2.6 Synthesis of **25-33** 34

1.3. Synthesis of TCP related compounds 44

1.3.1. Synthesis of TCP-amide analogues **38-41** 44

1.3.2 Synthesis of starting material **42** 52

1.3.3 Synthesis of TCP-phenyl carboxylic acid intermediates **43- 46** 56

1.3.4 Synthesis of VHL-based linker intermediate **50** 60

1.3.5 Synthesis of **49, 52** and **54** 63

2. Supplementary Information: Biology 70

2.1. LSD-HDAC1-CoREST expression and purification 70

2.2. SP2509/SP2577 analogues LSD1 HRP-coupled demethylase assay protocol 73

2.3. TCP analogues LSD1 HRP-coupled demethylase assay protocol 76

2.4. CTG cell viability assays 79

2.5 Protocols for LSD1 degradation and histone methylation in HCT116 cells 80

2.6 Western blots for LSD1 degradation and histone methylation with SP2509/SP2577 analogues – 24h. 81

2.7 Western blots for LSD1 degradation and histone methylation with TCP analogues – 24h 83

2.8 Western blots for LSD1 degradation and histone methylation with selected TCP analogues – 48h and 72h 84

3. References 86

Appendix: Analytical data for the final compounds 88

1. Supplementary Information: Chemistry

1.1 General Information

All reagents and solvents were obtained from Sigma Aldrich, Acros Organics, Fluorochem, Fisher Scientific and were used as supplied unless stated otherwise. Compounds **VHL-NH$_2$**, **47** and racemic Tranylcypromine (TCP) hydrochloride were purchased from TOCRIS. Biotage® Macroporous polystyrene-co-divinylbenzene (MP) carbonate resin (3.02 mmol/g loading capacity) was used for neutralising amine TFA salts and scavenging excess TFA during tert-butoxycarbonyl deprotection reactions. Room temperature refers to ambient temperature. Temperatures of 0 °C were maintained using an ice-water bath. The reactions were monitored by thin-layer chromatography (TLC) on aluminum backed silica gel. Unless otherwise stated Flash column chromatography was carried out with Silica Gel 60 using commercial solvents. All evaporations *in vacuo* were performed under reduced pressure using a Büchi rotary evaporator. All chemical names have been generated using ChemDraw Professional.

Nuclear magnetic resonance (NMR) spectra were acquired using a Bruker 400 (^{1}H, 400 MHz; ^{13}C 101 MHz) instrument at ambient temperature using deuterated solvent as reference - $CDCl_3$ (δ_H = 7.26 ppm, δ_C = 77.00 ppm), DMSO-*d$_6$* (δ_H = 2.50 ppm, δ_C = 39.51 ppm) or CD_3OD (δ_H = 3.31 ppm, δ_C = 49.15 ppm). ^{1}H NMR data are reported as: chemical shift, multiplicity [b, broad; s, singlet; d, doublet; t, triplet; q, quartet; quin, quintet m for multiplet; or as a combination (e.g., dd, dt, etc.)], coupling constant(s) and integration. ^{13}C NMR spectra were recorded by broadband proton decoupling. Stereochemistry of final compounds is assumed to be retained through all synthetic steps and purification procedures, due to only single peaks and/or splitting patterns with no shoulders being observed in the HPLC and NMR data respectively for these final compounds.

1.1.1. Synthesis of Cereblon-based amine **CRBN-NH2**

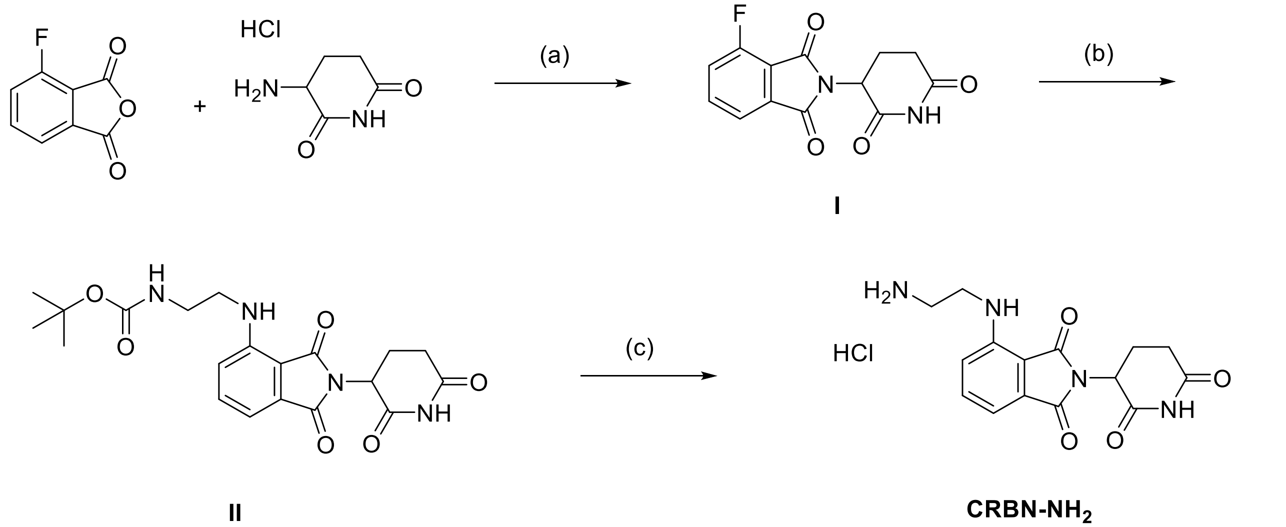

Reagents and conditions (a) NaOAc, AcOH, reflux, 20 h, 83 %; (b) Tert-butyl (2-aminoethyl)carbamate, DIPEA, DMF, 90 °C, 16 hr, 28 %; (c) HCl (4 N in 1,4-dioxane), DCM, rt, 4 hr, 99 %.

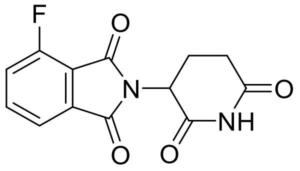

2-(2,6-dioxopiperidin-3-yl)-4-fluoroisoindoline-1,3-dione, I: 3-aminopiperidine-2,6-dione hydrochloride (1.008 g, 6.12 mmol) and sodium acetate (0.602 g, 7.34 mmol) were added to a solution of 3-fluorophthalic anhydride (1.003 g, 6.04 mmol) in AcOH (20 mL), then the resultant mixture was refluxed at 140 ˚C overnight.

The reaction mixture was cooled to room temperature and then concentrated *in vacuo* to afford a grey/black solid (2.159 g). The crude product was purified by column chromatography (dry load, 1-6% MeOH in DCM) to afford **I** (1.396 g, 5.00 mmol, 83% yield) as a powdery white solid.

^{1}H NMR (400 MHz, DMSO-d_6) δ ppm 11.15 (s, 1 H), 7.95 (ddd, *J*=8.3, 7.3, 4.6 Hz, 1 H), 7.79 (d, *J*=7.3 Hz), 7.73 (dd, *J*=9.3, 8.3 Hz, 1 H), 5.16 (dd, *J*=12.8, 5.4 Hz, 1 H), 2.89 (ddd, *J*=17.2, 13.9, 5.4 Hz, 1 H), 2.57 - 2.65 (m, 1 H), 2.50 - 2.55 (m, 1 H), 2.01 - 2.11 (m, 1 H).

^{13}C NMR (101 MHz, DMSO-d_6) δ ppm 172.7, 169.7, 166.1, 163.9, 156.8, 138.0, 133.4, 123.0, 120.0, 117.0, 49.1, 30.9, 21.8.

^{19}F NMR (376 MHz, DMSO-d_6) δ ppm -114.7.

HRMS (ESI) m/z: $[M+Na]^+$ calculated for $C_{13}H_{10}FN_2O_4$: 277.0625, found 277.0626.

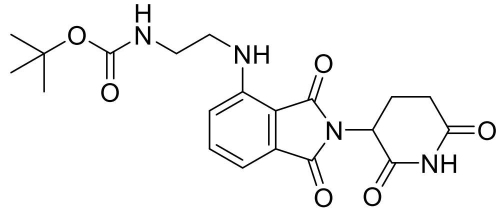

Tert-butyl (2-((2-(2,6-dioxopiperidin-3-yl)-1,3-dioxoisoindolin-4-yl)amino)ethyl)carbamate, II: To a solution of **I** (1.177 g, 4.26 mmol) in dry DMF (20 mL), DIPEA (1.48 mL, 8.52 mmol) and tert-butyl (2-aminoethyl)carbamate (0.742 mL, 4.69 mmol mmol) was added and the resultant solution was stirred at 90°C for 16 hours.

The reaction mixture was cooled to room temperature then poured in water (30 mL) and extracted with EtOAc (2 x 50 mL). The organic layers were combined, washed with sat. brine (2 x 50 mL), dried over Na_2SO_4 then concentrated *in vacuo* to afford a dark green tar.

The crude product was purified by column chromatography (10-100% EtOAc in hexane) to afford **II** (0.505 g, 1.20 mmol, 28% yield) as a yellow/green solid.

^1H NMR (400 MHz, $CDCl_3$) δ ppm 8.37 (br s, 1 H), 7.50 (dd, *J*=8.3, 7.3 Hz, 1 H), 7.11 (d, *J*=7.3 Hz, 1 H), 6.98 (d, *J*=8.3 Hz, 1 H), 6.40 (t, *J*=5.8 Hz, 1 H), 4.85 - 5.03 (m, 2 H), 3.41 - 3.50 (m, 2 H), 3.32 - 3.40 (m, 2 H), 2.72 - 2.92 (m, 3 H), 2.09 - 2.15 (m, 1 H), 1.45 (s, 9 H).

^{13}C NMR (101 MHz, $CDCl_3$) δ ppm 171.1, 169.4, 168.4, 167.5, 156.1, 146.8, 136.2, 132.5, 116.7, 111.9, 110.3, 79.8, 48.9, 42.5, 40.1, 31.4, 28.3, 22.7.

HRMS (ESI) m/z: $[M+H]^+$ calculated for $C_{20}H_{25}N_4O_6$: 417.1774, found 417.1774, $[M+Na]^+$ calculated for $C_{20}H_{24}N_4O_6Na$: 439.1594, found 439.1593.

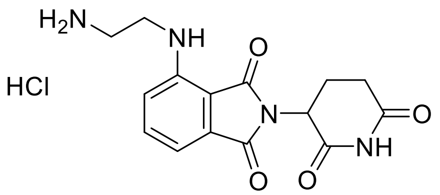

4-((2-aminoethyl)amino)-2-(2,6-dioxopiperidin-3-yl)isoindoline-1,3-dione hydrochloride, CRBN-NH$_2$: HCl in dioxane (4 N, 5 mL) was added to a stirring solution of **II** (483.3 mg, 1.16 mmol) in DCM (15 mL) and the resulting reaction mixture stirred at room temperature for 4 hours.

The reaction mixture was concentrated *in vacuo* to afford **CRBN-NH$_2$** (428.1 mg, 1.16 mmol, 99% yield) as a yellow solid.

^{1}H NMR (400 MHz, DMSO-d_6) δ ppm 11.10 (s, 1 H), 8.13 (br s, 3 H), 7.61 (dd, *J*=8.4, 7.2 Hz, 1 H), 7.25 (d, *J*=8.4 Hz, 1 H), 7.08 (d, *J*=7.2 Hz, 1 H), 6.83 (br t, *J*=6.1 Hz, 1 H), 5.07 (dd, *J*=12.8, 5.4 Hz, 1 H), 3.62 (q, *J*=6.0 Hz, 2 H), 2.97 (q, *J*=6.1 Hz, 2 H), 2.84 - 2.93 (m, 1 H), 2.51 - 2.65 (m, 2 H), 1.98 - 2.07 (m, 1 H).

^{13}C NMR (101 MHz, DMSO-d_6) δ ppm 172.8, 170.1, 168.6, 167.2, 145.8, 136.3, 132.3, 117.3, 111.0, 110.0, 48.5, 39.8, 37.6, 31.0, 22.2.

HRMS (ESI) m/z: $[M+H]^+$ calculated for $C_{15}H_{17}N_4O_4$: 317.1250, found 317.1254.

1.2. Synthesis of SP2509 and SP2577 related compounds and PROTACs

1.2.1. Synthesis of precursor hydrazides **5-6**

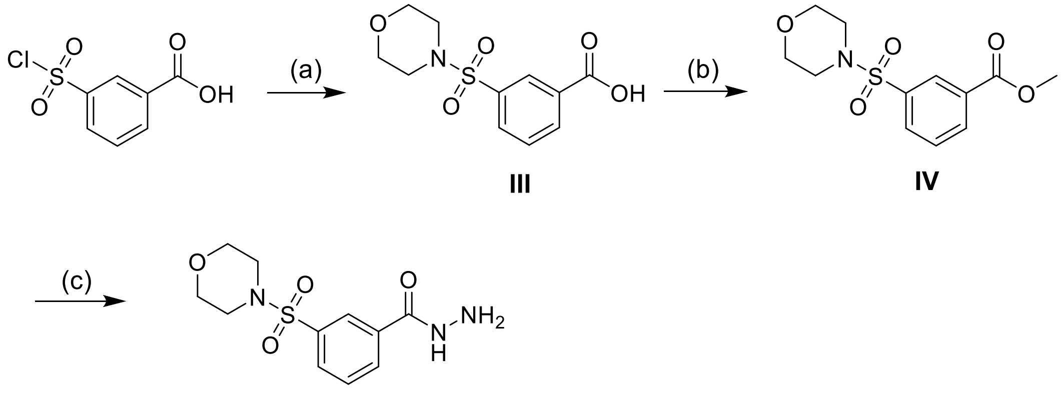

5

Reagents and conditions (a) morpholine, THF, r.t., 72 hrs, 91 %; (b) MeI, K_2CO_3, DMF, r.t., 3 hr, 86 %; (c) Hydrazine monohydrate, MeOH, 60 °C, 18 hr, 80 %.

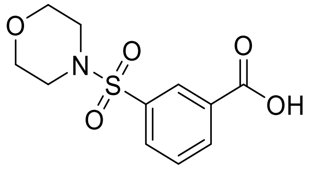

3-(Morpholinosulfonyl)benzoic acid, III: To a solution of 3-(chlorosulfonyl)benzoic acid (3.00 g, 13.6 mmol) in THF (20 mL) was added morpholine (12 mL, 139 mmol) which provided a white cloudy solution. The flask was stirred at room temperature for 72 hr, where TLC confirmed complete conversion. The reaction was quenched with HCl (3 M, aq.) until pH ~4 was obtained (the white solution turned pale yellow). The product was extracted into EtOAc (5 x 50 mL) and then washed with brine (100 mL). The organics were then dried over Na_2SO_4 (anhyd.), filtered and then solvent removed *in vacuo.* to yield **III** as a crystalline white solid (3.34 g, 91 %) which was used without further purification.

^{1}H NMR (400 MHz, $CDCl_3$) 8.49 (t, J = 1.4 Hz, 1H), 8.37 (dt, J = 7.9, 1.4 Hz, 1H), 8.02 (dt, J = 7.9, 1.4 Hz, 1H), 7.72 (t, J = 7.9 Hz, 1H), 3.78 (t, J = 4.7 Hz, 4H), 3.05 - 3.09 (m, 4H). Exchangeable proton not observed.

^{13}C NMR (101 MHz, $CDCl_3$) 168.8, 136.4, 134.4, 132.6, 130.5, 129.7, 129.4, 66.1, 46.0.

HRMS (ES^+) $C_{11}H_{14}NO_5S$ requires 272.0593 found 272.0593.

Spectroscopic data are consistent with previous literature.[1]

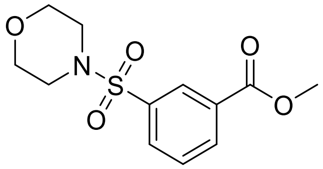

Methyl 3-(morpholinosulfonyl)benzoate, IV: Compound **III** (3.34 g, 13.6 mmol) and K_2CO_3 (3.76 g, 21.8 mmol) were flushed with N_2, and dissolved in DMF (40 mL). The mixture was stirred for 10 minutes after which methyl iodide (1.35 mL, 27.2 mmol) was added portion wise by needle-stick and the reaction stirred at r.t. for 3 hr, where TLC confirmed complete conversion. The reaction was quenched with NH_4Cl (10 % aq., 40 mL) and then extracted into EtOAc (5 x 100 mL). The organics were then dried over Na_2SO_4 (anhyd.), filtered and then solvent removed *in vacuo.* to yield **IV** as an off-white crystalline solid (3.32 g, 86 %) which was used without further purification.

^1H NMR (400 MHz, $CDCl_3$) 8.41 (td, J = 1.6, 0.5 Hz, 1 H), 8.30 (ddd, J = 7.9, 2 × 1.6 Hz, 1 H), 7.95 (ddd, J = 7.9, 2 × 1.6 Hz, 1 H), 7.67 (td, J = 7.9, 0.5 Hz, 1 H), 3.98 (s, 3 H), 3.74 - 3.78 (m, 4 H), 3.01 - 3.06 (m, 4 H).

^{13}C NMR (101 MHz, $CDCl_3$) 165.4, 136.0, 133.9, 131.8, 131.5, 129.5, 128.8, 66.1, 52.7, 46.0.

HRMS (ES+) $C_{12}H_{16}NO_5S$ requires 286.0749 found 286.0753.

Spectroscopic data are consistent with previous literature.[2]

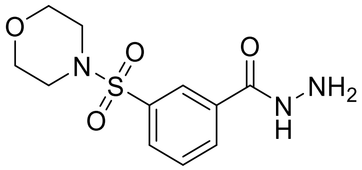

3-(Morpholinosulfonyl)benzohydrazide, 5: Compound **IV** (3.32 g, 11.6 mmol) was dissolved in bench MeOH (10 mL) and cooled in an ice bath. Hydrazine monohydrate (7.2 mL, 148 mmol) was added and the mixture heated and stirred at 60 °C for 18 hr, where TLC confirmed complete

conversion. The solvent was removed *in vacuo.* and the product recrystallized from hot methanol, yielding **5** as a crystalline white solid (2.66 g, 80 %).

^{1}H NMR (400 MHz, DMSO-d_6) 10.10 (br s, 1 H), 8.16 (ddd, J = 7.5, 2 × 1.7 Hz, 1 H), 8.13 (td, J = 1.7, 0.6 Hz, 1 H), 7.87 (ddd, J = 7.5, 2 × 1.7 Hz, 1 H), 7.76 (td, J = 7.5, 0.6 Hz, 1 H), 4.67 (br s, 2 H), 3.59 - 3.66 (m, 4 H), 2.85 - 2.92 (m, 4 H).

^{13}C NMR (101 MHz, DMSO-d_6) 164.6, 135.3, 134.9, 132.1, 130.5, 130.3, 126.5, 65.7, 46.4.

^{15}N NMR (40 MHz, DMSO-d_6) 98.5, 130.5, 200.0.

HRMS (ES+) $C_{11}H_{16}N_3O_4S$ requires 286.0862 found 286.0861.

Spectroscopic data are consistent with previous literature.[3]

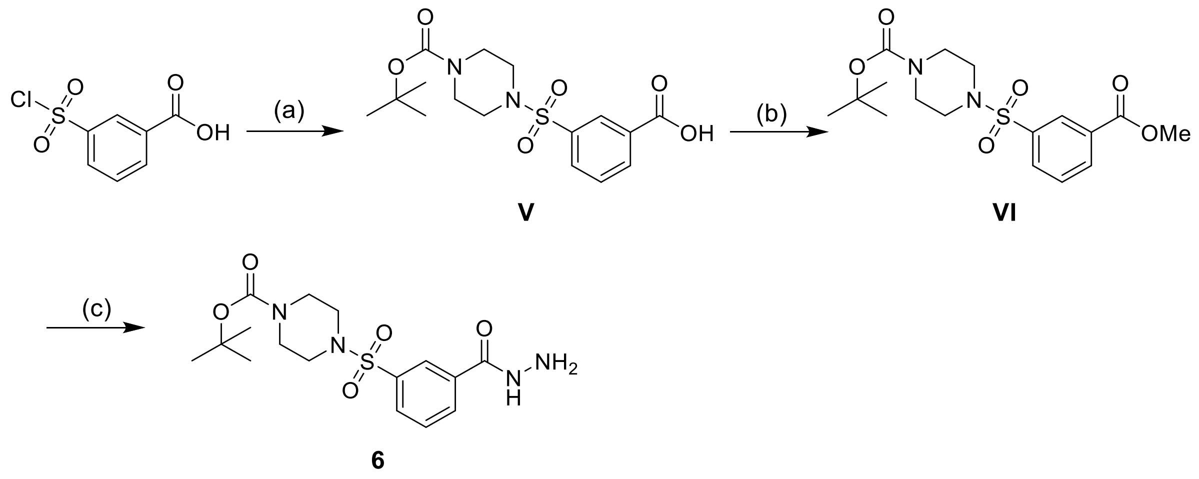

Reagents and conditions. (a) 1-Boc-piperazine, NEt_3, MeCN, r.t., 48 hr, 70 %; (b) MeI, K_2CO_3, DMF, r.t., 2 hr, 98 %; (c) $NH_2NH_2{\cdot}H_2O$, MeOH, 60 °C, 18 hr, 83 %.

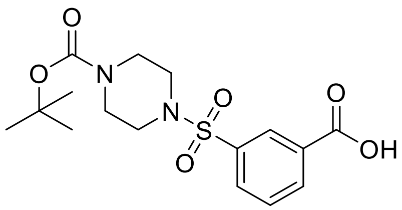

3-((4-(Tert-butoxycarbonyl)piperazin-1-yl)sulfonyl)benzoic acid, V: To a solution of 2-chlorobenzoic acid (2.02 g, 9.07 mmol) in acetonitrile (10 mL) was added 1-Boc-piperazine (6.53 g, 36.3 mmol) followed by triethylamine (3.80 mL, 27.2 mmol). The flask was stirred at room temperature for 40 hr, where TLC confirmed complete conversion. The solvent was removed *in vacuo.*, and then taken up in $NaHCO_3$ (sat. aq., 50 mL), shaken vigorously and then taken to pH 1 with HCl (3 M, aq.). The product was extracted into EtOAc (3 x 200 mL). The organics were then dried over Na_2SO_4 (anhyd.), filtered and then solvent removed *in vacuo.* to yield **V** as a crystalline white solid (3.23 g, 96 %) which was used without further purification.

^{1}H NMR (400 MHz, $CDCl_3$) δ ppm 8.46 (s, 1 H), 8.34 (d, J = 7.5 Hz, 1 H), 7.99 (d, J = 7.9 Hz, 1 H), 7.69 (t, J = 7.8 Hz, 1 H), 3.50 - 3.58 (m, 4 H), 2.99 - 3.07 (m, 4 H), 1.42 (s, 9 H). Note: Exchangeable proton not observed.

^{13}C NMR (101 MHz, DMSO-*d*$_6$) δ ppm 166.4, 153.8, 135.85, 134.3, 132.6, 132.0, 130.73, 128.4, 79.8, 46.1, 42.7, 28.4.

HRMS (ES-) $C_{16}H_{21}N_2O_6S$ requires 369.1120 found 369.1120.

Spectroscopic data are consistent with previous literature.[4]

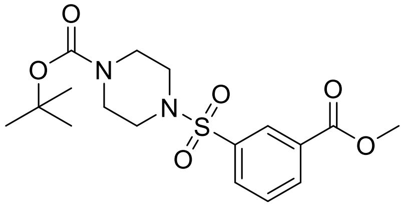

Tert-butyl 4-((3-(methoxycarbonyl)phenyl) sulfonyl)piperazine-1-carboxylate, VI: Compound **V** (3.23 g, 8.71 mmol) was dissolved in DMF (40 mL) and K_2CO_3 (2.40 g, 17.4 mmol) was added. The mixture was stirred for 10 minutes after which methyl iodide (0.98 mL, 15.68 mmol) was added portionwise. The flask was stirred at room temperature for 2 hours, where TLC confirmed complete conversion. The reaction was quenched with NH_4Cl (10 % aq., 60 mL) and then extracted into diethyl ether (5 x 150 mL) and washed with LiCl (5 % aq., 3 x 50 mL). The organics were then dried over Na_2SO_4 (anhyd.), filtered and then solvent removed *in vacuo.* to yield **VI** as a beige amorphous solid (3.12 g, 93 %) which was used without further purification.

^{1}H NMR (400 MHz, $CDCl_3$) δ ppm 8.40 (t, J = 1.6 Hz, 1 H), 8.30 (dt, J = 7.7, 1.6 Hz, 1 H), 7.95 (dt, J = 7.7, 1.6 Hz, 1 H), 7.66 (t, J = 7.7 Hz, 1 H), 3.98 (s, 3 H), 3.50 - 3.55 (m, 4 H), 3.01 (m, 4 H), 1.41 (s, 9 H).

^{13}C NMR (101 MHz, $CDCl_3$) δ ppm 164.4, 153.1, 135.3, 132.9, 130.7, 130.5, 128.5, 127.7, 79.5, 51.7, 44.9, 41.9, 27.3.

HRMS (ES+) $C_{17}H_{24}N_2O_6SNa$ requires 407.1253 found 407.1255.

Spectroscopic data are consistent with previous literature.[5]

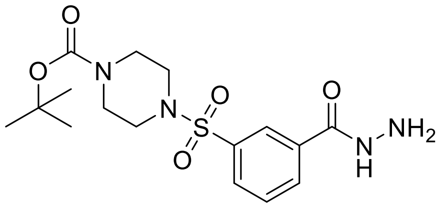

Tert-butyl 4-((3-(hydrazinecarbonyl)phenyl) sulfonyl)piperazine-1-carboxylate, 6: Compound **VI** (3.12 g, 8.11 mmol) was dissolved in MeOH (140 mL) and then cooled to 0 °C. Hydrazine monohydrate (5.11 mL, 105 mmol) was added and the mixture was stirred at 60 °C for 24 hr. The

solvent was removed *in vacuo.* and the product recrystallized from hot methanol, yielding **6** as a crystalline white solid (2.62 g, 84 %).

Melting point: 200.7 – 201.4 °C

^{1}H NMR (400 MHz, DMSO-d_6) δ ppm 10.08 (br s, 1 H), 8.14 (d, J = 7.6 Hz, 1 H), 8.12 (s, 1 H), 7.87 (d, J = 7.6 Hz, 1 H), 7.75 (t, J = 7.6 Hz, 1 H), 4.58 (br s, 2 H), 3.39 (br t, J = 5.0 Hz, 4 H), 2.88 (t, J = 5.0 Hz, 4 H), 1.34 (s, 9 H).

^{13}C NMR (101 MHz, DMSO-d_6) δ ppm 164.7, 153.8, 135.7, 134.9, 132.1, 130.4, 130.3, 126.3, 79.8, 46.2, 42.9, 28.4.

HRMS (ES+) $C_{16}H_{25}N_4O_5S$ requires 385.1546 found 385.1543, $C_{16}H_{24}N_4O_5SNa$ requires 407.1365 found 407.1357.

IR (neat) / cm^{-1} 3326, 3258, 1682, 1631, 1349, 1163, 1126.

1.2.2. Synthesis of hydrazones **3** (SP2509) and **8-11**

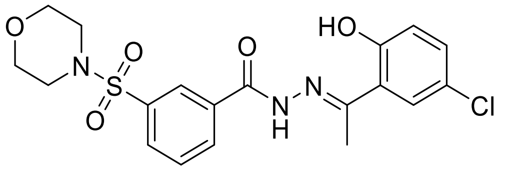

N′**-(1-(5-chloro-2-hydroxyphenyl)ethylidene)-3-(morpholinosulfonyl)benzohydrazide, 3** (SP2509)**:** **5** (50.3 mg, 0.18 mmol) and 2′-hydroxy-5′-chloroacetophenone (33.0 mg, 0.19 mmol) in ethanol (4 mL) was refluxed for 24 hours, where TLC confirmed complete conversion. The solvents were removed *in vacuo.* and the resulting solid was purified by column chromatography (0-5 % MeOH in DCM) to yield **3** as a brown solid (47.2 mg, 61%).

^{1}H NMR (400 MHz, DMSO-*d*$_6$) δ ppm 13.36 (br s, 1 H), 11.71 (s, 1 H), 8.30 (d, J = 7.9 Hz, 1 H), 8.21 (s, 1 H), 7.98 (d, J = 7.9 Hz, 1 H), 7.82 - 7.88 (t, J = 7.9 Hz, 1 H), 7.68 (d, J = 2.6 Hz, 1 H), 7.36 (dd, J = 8.8, 2.6 Hz, 1 H), 6.97 (d, J = 8.8 Hz, 1 H), 3.63 - 3.67 (m, 4 H), 2.90 - 2.95 (m, 4 H), 2.51 (m, 3 H).

^{13}C NMR (101 MHz, DMSO-*d*$_6$) δ ppm 163.8, 158.2, 158.0, 135.3, 134.6, 133.4, 131.4, 131.3, 130.3, 128.4, 127.6, 122.6, 121.2, 119.6, 65.7, 46.4, 14.9.

IR (neat) / cm^{-1} 3568, 3459, 3283, 1677, 577

HRMS (ES+): $C_{19}H_{21}N_3O_5S^{35}Cl$ requires 438.0890 found 438.0891, $C_{19}H_{21}N_3O_5S^{37}Cl$ requires 440.0861 found 440.0868.

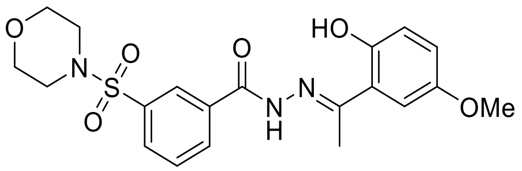

N′**-(1-(2-hydroxy-5-methoxyphenyl)ethylidene)-3-(morpholinosulfonyl)benzohydrazide, 8: 5** (50.0 mg, 0.175 mmol) and 2′-hydroxy-5′-methoxyacetophenone (29.8 mg, 0.179 mmol) in ethanol (4 mL) was refluxed for 48 hours, where TLC confirmed complete conversion. The solvents were removed *in vacuo.* and the resulting solid was purified by column chromatography (3 % MeOH in DCM) to yield **8** as a crystalline orange solid (35.8 mg, 47%).

Melting point: 188.7 – 189.3 °C

^{1}H NMR (400 MHz, DMSO-d_6) δ ppm 12.75 (br s, 1 H), 11.60 (br s, 1 H), 8.29 (d, J = 7.9 Hz, 1 H), 8.20 (s, 1 H), 7.96 (br d, J = 7.9 Hz, 1 H), 7.84 (t, J = 7.9 Hz, 1 H), 7.14 (d, J = 2.9 Hz, 1 H), 6.96 (dd, J = 8.9, 2.9 Hz, 1 H), 6.86 (d, J = 8.9 Hz, 1 H), 3.76 (s, 3 H), 3.62 - 3.67 (m, 4 H), 2.89 - 2.95 (m, 4 H), 2.48 (m, 3 H).

^{13}C NMR (101 MHz, DMSO-d_6) δ ppm 163.7, 159.2, 153.3, 151.9, 135.3, 135.0, 133.36, 131.1, 130.3, 127.6, 119.8, 118.4, 118.3, 113.5, 79.7, 65.7, 56.1, 46.4, 14.9.

IR (neat) / cm^{-1} 3472, 3276, 3068, 1668, 1348, 1170.

HRMS (ES+) $C_{20}H_{24}N_3O_6S$ requires 434.1386 found 434.1382.

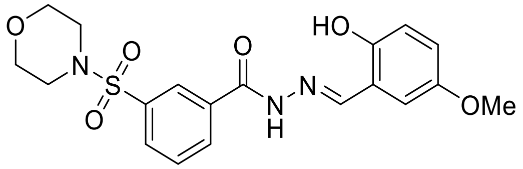

***N'*-(2-hydroxy-5-methoxybenzylidene)-3-(morpholinosulfonyl)benzohydrazide, 9: 5** (50.3 mg, 0.176 mmol) and 2-hydroxy-5-methoxybenzaldehyde (24.2 µL, 0.194 mmol) in ethanol (3.5 mL) was refluxed for 48 hours, where TLC confirmed complete conversion. The solvents were removed *in vacuo.* and the resulting solid was purified by column chromatography (1-3 % MeOH in DCM) to yield **9** as a crystalline brown solid (67.9 mg, 92%).

Melting point 110.6 – 112.0 °C

^{1}H NMR (400 MHz, DMSO-d_6) δ ppm 12.30 (br s, 1 H), 10.56 (s, 1 H), 8.69 (s, 1 H), 8.26 - 8.33 (m, 2 H), 7.97 (d, J = 8.0 Hz, 1 H), 7.86 (t, J = 8.0 Hz, 1 H), 7.17 (d, J = 3.0 Hz, 1 H), 6.94 (dd, J = 8.6, 3.0 Hz, 1 H), 6.88 (d, J = 8.6 Hz, 1 H), 3.74 (s, 3 H), 3.60 - 3.69 (m, 4 H), 2.87 - 2.97 (m, 4 H).

^{13}C NMR (101 MHz, DMSO-d_6) δ ppm 161.8, 152.7, 152.0, 148.5, 135.5, 134.6, 133.0, 131.2, 130.5, 127.0, 119.5, 119.0, 117.8, 112.4, 65.7, 56.0, 46.4.

IR (neat) / cm^{-1} 3447, 3233, 3068, 1641, 1350, 1173, 1163.

HRMS (ES+) $C_{19}H_{22}N_3O_6S$ requires 420.1229 found 420.1224; $C_{19}H_{21}N_3O_6SNa$ requires 442.1049 found 442.1040.

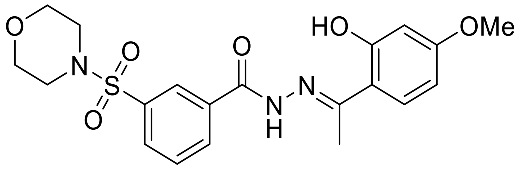

***N′*-(1-(2-hydroxy-4-methoxyphenyl)ethylidene)-3-(morpholinosulfonyl)benzohydrazide, 10: 5** (49.7 mg, 0.174 mmol) and 2′-hydroxy-4′-methoxyacetophenone (32.0 mg, 0.193 mmol) in ethanol (4 mL) was refluxed for 20 hours, where TLC confirmed complete conversion. The solvents were removed *in vacuo.* and the resulting solid was purified by column chromatography (1-8 % MeOH in DCM) to yield **10** as a crystalline orange solid (63.3 mg, 84 %).

Melting point: 204.8 – 205.7 °C

^{1}H NMR (400 MHz, DMSO-d_6) δ ppm 13.60 (br s, 1 H), 11.52 (br s, 1 H), 8.28 (d, J = 7.7 Hz, 1 H), 8.20 (s, 1 H), 7.96 (br d, J = 7.7 Hz, 1 H), 7.85 (t, J = 7.7 Hz, 1 H), 7.57 (d, J = 8.7 Hz, 1 H), 6.44 - 6.54 (m, 2 H), 3.79 (m, 3 H), 3.61 - 3.68 (m, 4 H), 2.88 - 2.95 (m, 4 H), 2.47 (s, 3 H).

^{13}C NMR (101 MHz, DMSO-d_6) δ ppm 163.4, 162.5, 161.3, 160.2, 135.3, 134.9, 133.3, 131.1, 130.4, 130.3, 127.5, 113.0, 106.3, 102.0, 65.7, 55.7, 46.4, 14.8.

IR (neat) / cm^{-1} 3140, 3077, 1660, 1348, 1170.

HRMS (ES+) $C_{20}H_{24}N_3O_6S$ requires 434.1386 found 434.1390.

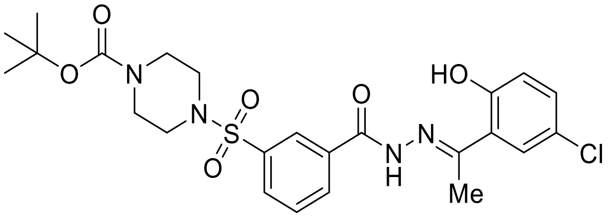

Tert-butyl 4-((3-(2-(1-(5-chloro-2-hydroxyphenyl)ethylidene)hydrazine-1-carbonyl)phenyl)sulfonyl)piperazine-1-carboxylate, 11: 6 (163 mg, 0.424 mmol) and 2′-hydroxy-5′-chloroacetophenone (79.5 mg, 0.466 mmol) in ethanol (6 mL) was refluxed for 48 hours, where TLC confirmed complete conversion. The solvents were removed *in vacuo.* and the resulting solid

was purified by column chromatography (0-5 % MeOH in DCM) to yield **11** as a crystalline beige solid (193 mg, 85%).

Melting point: 222 – 223.9 °C

^{1}H NMR (400 MHz, DMSO-d_6) δ ppm 13.34 (br s, 1 H), 11.68 (s, 1 H), 8.27 (d, J = 7.9 Hz, 1 H), 8.19 (s, 1 H), 7.96 (br d, J = 7.9 Hz, 1 H), 7.83 (t, J = 7.9 Hz, 1 H), 7.67 (d, J = 2.3 Hz, 1 H), 7.35 (dd, J = 8.6, 2.3 Hz, 1 H), 6.96 (d, J = 8.6 Hz, 1 H), 3.41 (m, 4 H), 2.91 (br t, J = 4.8 Hz, 4 H), 2.50 (m, 3 H)*, 1.33 (s, 9 H).

^{13}C NMR (101 MHz, DMSO-d_6) δ ppm 163.3, 157.6, 157.5, 153.3, 135.2, 134.2, 132.9, 131.0, 130.7, 129.8, 127.9, 127.0, 122.1, 120.8, 119.2, 79.4, 45.8, 42.7, 27.9, 14.4.

IR (neat) / cm^{-1} 3287, 1689, 1654, 1354, 1170.

HRMS (ES+) $C_{24}H_{30}N_4O_6S^{35}Cl$ requires 537.1575 found 537.1580, $C_{24}H_{30}N_4O_6S^{37}Cl$ requires 539.1545 found 539.1555; $C_{24}H_{29}N_4O_6S^{35}ClNa$ requires 559.1394 found 559.1391, $C_{24}H_{29}N_4O_6S^{37}ClNa$ requires 561.1365 found 561.1372.

* fully overlapping with ^{1}H signal, presence identified by cross-peak visible in HSQC-DEPT experiment.

1.2.3. Synthesis of acetophenone precursors to **12** and **13**

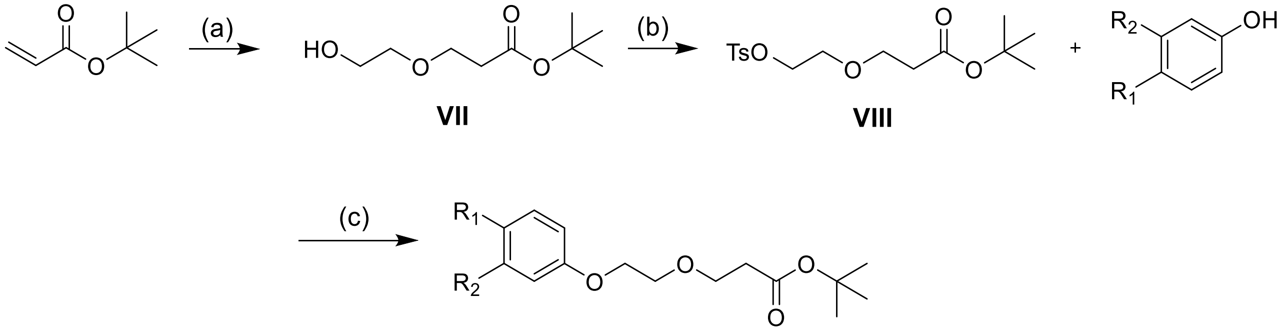

IXa R_1=OH, R_2=C(O)CH_3
IXb R_1=C(O)CH_3 R_2=OH

(a) Ethylene glycol, sodium (0.05 equiv.), THF, 40 hr, 31 %; (b) TsCl, NEt_3, DCM, 84 %; (c) K_2CO_3, MeCN, reflux, 20 hr, 42 % (**IXa**), 60 % (**IXb**).

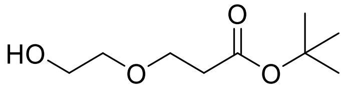

Tert-butyl 3-(2-hydroxyethoxy)propanoate, VII: Ethylene glycol (17 mL, 304 mmol) was added to dry THF (40 mL) and allowed to stir vigorously for ~5 mins. Sodium (70 mg, 3.05 mmol) was added and the flask was stirred for 2 hr, after which tert-butyl acrylate (7.5 mL, 51.2 mmol) was added. The flask was stirred at ambient temperature for 40 hours. The solution was neutralised to pH 7 with HCl (2 M, aq.) and extracted into EtOAc (3 x 30 mL). The organics were dried over sodium sulfate (anhyd.), filtered and solvents removed *in vacuo.* and purified by flash column chromatography (EtOAc) to give **VII** as a pale yellow oil (2.98 g, 31 %).

^{1}H NMR (400 MHz, $CDCl_3$) 3.62 - 3.69 (m, 4 H), 3.49 - 3.53 (m, 2 H), 2.44 (t, J = 6.1 Hz, 2 H), 2.37 (br s, 1 H), 1.39 (s, 9 H).

^{13}C NMR (101 MHz, $CDCl_3$) 171.3, 80.9, 71.9, 66.4, 61.6, 36.1, 28.1.

IR (neat) / cm^{-1} 3437, 2978, 2932, 2873, 1726, 1155, 1118, 1054.

HRMS (ES+) $C_9H_{18}O_4Na$ requires 213.1103 found 213.1102.

Spectroscopic data are consistent with previous literature.[6]

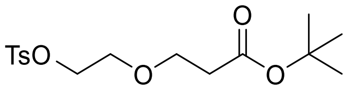

Tert-butyl 3-(2-(tosyloxy)ethoxy)propanoate, VIII: To a flask containing **VII** (498.7 mg, 2.616 mmol) in DCM (3 mL) was added triethylamine (0.5 mL, 3.587 mmol), which was stirred for 5 mins. TsCl (544.3 mg, 2.855 mmol) in DCM (10 mL) was added to the flask and stirred vigorously for 72 hours. Solvent removed *in vacuo.* and the resulting suspension was purified by flash column chromatography (15 – 40 % EtOAc in petroleum ether (40-60 °C)) to yield **VIII** as a crystalline white solid (0.7526 g, 84 %).

Melting point: 51.5 – 53.0 °C

^{1}H NMR (400 MHz, $CDCl_3$) 7.81 (d, J = 7.6 Hz, 2 H), 7.36 (d, J = 7.6 Hz, 2 H), 4.13 - 4.18 (m, 2 H), 3.62 - 3.68 (m, 4 H), 2.46 (s, 3 H), 2.41 - 2.45 (m, 2 H), 1.44 - 1.47 (m, 9 H).

^{13}C NMR (101 MHz, $CDCl_3$) 170.6, 144.8, 133.0, 129.8, 128.0, 80.7, 69.1, 68.4, 67.0, 36.1, 28.1, 21.6.

IR (neat) / cm^{-1} 2974, 2900, 1736, 1349, 1166, 1149, 1126, 918

HRMS (ES+) $C_{16}H_{24}O_6NaS$ requires 367.1191 found 367.1193.

Spectroscopic data are consistent with previous literature.[6]

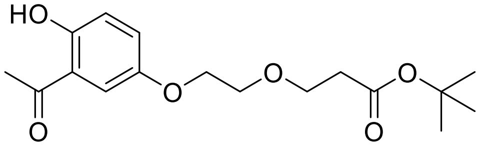

Tert-butyl 3-(2-(3-acetyl-4-hydroxyphenoxy)ethoxy)propanoate IXa:

2′,5′-Dihydroxyacetophenone (703 mg, 4.62 mmol) and K_2CO_3 (784 mg, 5.67 mmol) were backfilled with nitrogen and refluxed in acetonitrile (13 mL) for 30 mins, after which **VIII** (404 mg, 1.17 mmol) in acetonitrile (2 mL) was added, and the resulting mixture was heated at reflux for 20 hours. The solvent was removed, taken up in chloroform and filtered through celite. Chloroform solvent removed *in vacuo.*, leaving black oils. These oils were purified by flash column chromatography (15 – 30 % EtOAc in n-hexanes) to yield **IXa** as a yellow oil (162 mg, 42 %).

^{1}H NMR (400 MHz, $CDCl_3$) δ ppm 11.85 (s, 1 H), 7.24 (d, J = 3.0 Hz, 1 H), 7.13 (dd, J = 9.1, 3.0 Hz, 1 H), 6.91 (d, J = 9.1 Hz, 1 H), 4.07 - 4.12 (m, 2 H), 3.76 - 3.83 (m, 4 H), 2.61 (s, 3 H), 2.54 (t, J = 6.5 Hz, 2 H), 1.45 (s, 9 H).

^{13}C NMR (101 MHz, $CDCl_3$) δ ppm 204.0, 170.7, 156.8, 150.8, 124.8, 119.1, 119.0, 115.0, 80.5, 69.4, 68.4, 67.0, 36.1, 28.0, 26.6.

IR (neat) / cm^{-1} 2978, 2930, 2875, 1726, 1485, 1365, 1287, 1157, 1120, 1050;

HRMS (ES+) $C_{17}H_{24}O_6Na$ requires 347.1471 found 347.1472.

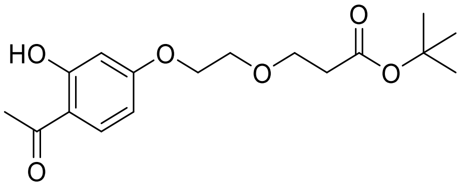

Tert-butyl 3-(2-(4-acetyl-3-hydroxyphenoxy)ethoxy)propanoate IXb: 2′,4′-Dihydroxyacetophenone (707 mg, 4.65 mmol) and K_2CO_3 (799 mg, 5.78 mmol) were backfilled with nitrogen and refluxed in acetonitrile (13 mL) for 30 mins, after which **VIII** (400 mg, 1.16 mmol) in acetonitrile (2 mL) was added, and the resulting mixture was heated at reflux for 20 hours. The solvent was removed, taken up in chloroform and filtered through celite. Chloroform solvent removed *in vacuo.*, leaving black oils. These oils were purified by flash column chromatography (20 % EtOAc in n-hexanes) to yield **IXb** as a yellow oil (225 mg, 60 %).

^{1}H NMR (400 MHz, $CDCl_3$) δ ppm 12.72 (s, 1 H), 7.63 (d, J = 8.9 Hz, 1 H), 6.47 (dd, J = 8.9, 2.5 Hz, 1 H), 6.42 (d, J = 2.5 Hz, 1 H), 4.12 - 4.16 (m, 2 H), 3.76 - 3.84 (m, 4 H), 2.56 (s, 3 H), 2.53 (t, J = 6.5 Hz, 2 H), 1.46 (s, 9 H).

^{13}C NMR (101 MHz, $CDCl_3$) δ ppm 202.6, 170.83, 165.3, 165.1, 132.3, 114.0, 108.0, 101.5, 80.7, 69.0, 67.6, 67.1, 36.2, 28.1, 26.2.

IR (neat) / cm^{-1} 2977, 2930, 2877, 1726, 1627, 1365, 1252, 1155, 1120.

HRMS (ES+) $C_{17}H_{24}O_6Na$ requires 347.1471 found 347.1476.

1.2.4. Synthesis of hydrazones **2** (SP2577) and **11-15**

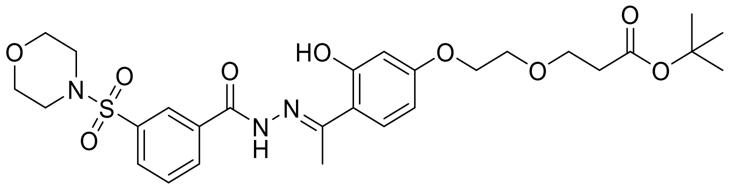

Tert-butyl 3-(2-(3-hydroxy-4-(1-(2-(3-(morpholinosulfonyl)benzoyl)hydrazineylidene)ethyl) phenoxy)ethoxy)propanoate, 12: 5 (96.7 mg, 0.339 mmol) and **IXb** (125 mg, 0.385 mmol) in ethanol (4.5 mL) was refluxed for 22 hours, where TLC confirmed complete conversion. The solvents were removed *in vacuo.* and the resulting solid was purified by column chromatography (30 – 100 % EtOAc in n-hexanes) to yield **12** as a yellow oil (129 mg, 63 %).

^{1}H NMR (400 MHz, $CDCl_3$) δ ppm 12.91 (br s, 1 H), 9.10 (br s, 1 H), 8.01 - 8.29 (m, 2 H), 7.93 (d, J = 8.0 Hz, 1 H), 7.71 (t, J = 8.0 Hz, 1 H), 7.39 (d, J = 8.9 Hz, 1 H), 6.55 (br s, 1 H), 6.50 (dd, J = 8.9, 2.4 Hz, 1 H), 4.08 - 4.21 (m, 2 H), 3.68 - 3.88 (m, 8 H), 3.03 - 3.09 (m, 4 H), 2.55 (t, J = 6.5 Hz, 2 H), 2.41 (s, 3 H), 1.47 (s, 9 H).

^{13}C NMR (101 MHz, $CDCl_3$) δ ppm 170.9, 162.2, 161.9, 161.5, 159.0, 136.0, 134.4, 132.2, 130.7, 129.9, 129.2, 126.5, 112.4, 107.0, 102.5, 80.7, 69.3, 67.4, 67.1, 66.0, 46.0, 36.3, 28.1, 13.3.

IR (neat) / cm^{-1} 3256, 1724, 1668, 1604, 1349, 1155, 1110.

HRMS (ES+) $C_{28}H_{38}N_3O_9S$ requires 592.2329 found 592.2330, $C_{28}H_{37}N_3O_9NaS$ requires 614.2148 found 614.2152.

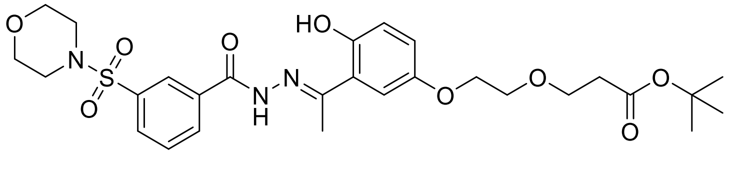

Tert-butyl 3-(2-(4-hydroxy-3-(1-(2-(3-(morpholinosulfonyl)benzoyl)hydrazineylidene)ethyl) phenoxy)ethoxy)propanoate, 13: 5 (120 mg, 0.421 mmol) and **IXa** (151 mg, 0.465 mmol) in ethanol (4.5 mL) was refluxed for 22 hours, where TLC confirmed complete conversion. The solvents were removed *in vacuo.* and the resulting solid was purified by column chromatography (50 – 100 % EtOAc in n-hexanes) to yield **13** as an amorphous pale yellow solid (156 mg, 64 %).

^{1}H NMR (400 MHz, $CDCl_3$) δ ppm 12.13 (br s, 1 H), 9.12 (br s, 1 H), 7.03 - 8.24 (m, 2 H), 7.94 (dt, J = 7.98, 1.3 Hz, 1 H), 7.71 (t, J = 7.79 Hz, 1 H), 7.07 (s, 1 H), 6.86 - 7.03 (m, 2 H), 4.02 (m, 2 H), 3.70 - 3.77 (m, 4 H), 3.62 - 3.70 (m, 4 H), 2.93 - 3.01 (m, 4 H), 2.47 (t, J = 6.5 Hz, 2 H), 2.33 (s, 3 H), 1.38 (s, 9 H).

^{13}C NMR (101 MHz, $CDCl_3$) δ ppm 170.9, 162.5, 158.0, 153.6, 151.1, 135.8, 134.3, 132.4, 130.7, 129.8, 126.6, 119.0, 118.8, 118.5, 114.3, 80.7, 69.6, 68.4, 67.1, 66.0, 46.0, 36.3, 28.1, 13.4.

IR (neat) / cm^{-1} 3266, 1723, 1676, 1349, 1155, 1112.

HRMS (ES+) $C_{28}H_{38}N_3O_9S$ requires 592.2329 found 592.2327, $C_{28}H_{37}N_3O_9NaS$ requires 614.2148 found 614.2149.

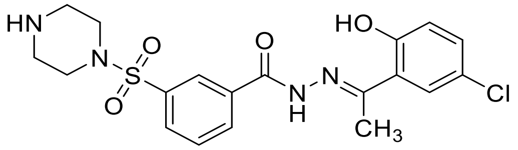

***N'*-(1-(5-chloro-2-hydroxyphenyl)ethylidene)-3-(piperazin-1-ylsulfonyl)benzohydrazide**, **14:** Compound **11** (777 mg, 1.45 mmol) was suspended in bench DCM (25 mL). TFA (2.5 mL) was added and the mixture stirred for 1 hr. The solvent was removed *in vacuo.* and then triturated with acetonitrile. The crude oil was purified by column chromatography (0.1 % NH_3, 9.9 % MeOH in DCM) to yield **14** as a crystalline green solid (518 mg, 81 %).

^{1}H NMR (400 MHz, DMSO-d_6) δ ppm 13.40 (br s, 1 H), 8.29 (br d, J = 7.8 Hz, 1 H), 8.21 (s, 1 H), 7.97 (br d, J = 7.8 Hz, 1 H), 7.85 (t, J = 7.8 Hz, 1 H), 7.67 (d, J = 2.4 Hz, 1 H), 7.36 (dd, J = 8.7, 2.4 Hz, 1 H), 6.96 (d, J = 8.7 Hz, 1 H), 2.90 (app. br s, 4 H), 2.82 (app. br s, 4 H), 2.51 (m, 3 H). Note: 1 exchangeable proton not observed.

^{13}C NMR (101 MHz, DMSO-d_6) δ ppm 163.8, 158.2, 158.1, 135.6, 134.6, 133.3, 131.4, 131.2, 130.3, 128.4, 127.5, 122.5, 121.2, 119.7, 46.1, 44.5, 14.9.

IR (neat) / cm^{-1} 2980, 2840, 1668, 1474, 1283, 1260, 1166 (Note: very wide, broad region seen from 3600 – 2400 cm^{-1});

HRMS (ES+) $C_{19}H_{22}N_4O_4S^{35}Cl$ requires 437.1050 found 437.1049, $C_{19}H_{22}N_4O_4S^{37}Cl$ requires 439.1021 found 439.1025.

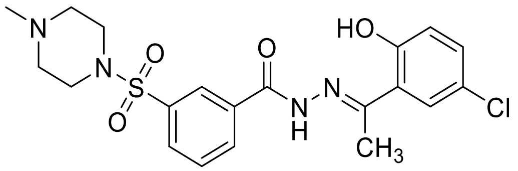

***N'*-(1-(5-chloro-2-hydroxyphenyl)ethylidene)-3-((4-methylpiperazin-1-yl)sulfonyl) benzohydrazide, 2 (SP2577):** To a flask containing **14** (50 mg, 0.114 mmol) in acetonitrile (4 mL) was added triethylamine (25 µL, 0.179 mmol). The flask was cooled to 0 °C and stirred for 10 mins, after which formaldehyde (37 % solution in H_2O/MeOH, 150 µL, 1.507 mmol) was added and stirred for a further 10 mins. $NaCNBH_3$ (12.0 mg, 0.1910 mmol) was added and the reaction was stirred at 0 °C for 1 hr. The solution was neutralised to pH 7 with acetic acid, and solvent removed *in vacuo.* The crude mixture was purified by column chromatography (5 % MeOH in DCM) and then by RP HPLC (40 – 100 % MeCN in H_2O, 30 min gradient, t_R = 20-25 mins) to yield **2** as a green paste (8.2 mg, 16 %); silica gel TLC R_f 0.14 (5 % MeOH in DCM);

^{1}H NMR (400 MHz, DMSO-d_6) δ ppm 8.35 - 8.41 (m, 2 H), 7.68 (d, J = 7.6 Hz, 1 H), 7.60 (t, J = 7.6 Hz, 1 H), 7.31 (d, J = 2.2 Hz, 1 H), 7.00 (dd, J = 8.7, 2.2 Hz, 1 H), 6.61 (d, J = 8.7 Hz, 1 H), 2.90 (br s, 4 H), 2.36 (br t, J = 4.5 Hz, 4 H), and 2.13 (s, 3 H).Note: 1 exchangeable proton not observed, also an expected CH_3 peak was directly overlapping with the residual DMSO-d_6 peak and was therefore not observed.

^{13}C NMR (101 MHz, DMSO-d_6) δ ppm 166.1, 164.6, 153.0, 134.6, 132.7, 129.6, 129.0, 128.0, 127.1, 126.5, 124.7, 121.5, 120.0, 116.9, 54.0, 46.3, 45.8, 12.5.

HRMS (ES+) $C_{19}H_{22}N_4O_4S^{35}Cl$ requires 451.1207 found 451.1210, $C_{19}H_{22}N_4O_4S^{37}Cl$ requires 453.1177 found 453.1192.

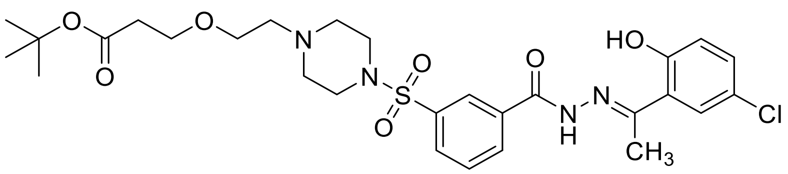

Tert-butyl 3-(2-(4-((3-(2-(1-(5-chloro-2-hydroxyphenyl)ethylidene)hydrazine-1-carbonyl) phenyl)sulfonyl)piperazin-1-yl)ethoxy)propanoate, 15: To oven dried glassware was added oxalyl chloride (2 M in DCM, 1.42 mL, 2.84 mmol) which was cooled to -78 °C. DMSO (0.5 mL, 7.04

mmol) in DCM (2 mL) was added which was stirred for 15 mins. Compound **VII** (295 mg, 1.55 mmol) in DCM (2 mL) was added dropwise to the flask over 20 mins, which was then stirred at -78 °C for 1 hr. Triethylamine (1.2 mL, 8.61 mmol) was added, the flask warmed to room temperature and then stirred for 30 mins. The mixture was washed with brine (3 x 10 mL) and then extracted into DCM (2 x 10 mL). The organics were combined and dried over anhyd. Na_2SO_4, filtered and solvent removed *in vacuo.* to yield the corresponding aldehyde as a crude orange oil (285 mg), which was immediately used as received without further characterisation.

In a clean flask containing **14** (102 mg, 0.232 mmol) in acetonitrile (8 mL) was added triethylamine (48 μL, 0.344 mmol). The flask was cooled to 0 °C and stirred for 10 mins, after which the crude aldehyde (39 mg, 0.207 mmol) was added and stirred for a further 10 mins. $NaCNBH_3$ (29.0 mg, 0.462 mmol) was added and the reaction was stirred at 0 °C for 4 hr. The solvent removed *in vacuo.* and purified by column chromatography (2 % MeOH in DCM) to yield **15** as an orange oil (38.0 mg, 30 %). A portion of this was further purified by RP HPLC (5 – 100 % MeCN in H_2O, 60 min gradient, t_R = 54.2 mins) to yield an amorphous beige solid (7.8 mg); silica gel TLC R_f 0.54 (10 % MeOH in DCM)

^{1}H NMR (400 MHz, Methanol-d_4) δ ppm 8.32 (s, 1 H), 8.26 (br d, J = 7.8 Hz, 1 H), 8.03 (br d, J = 7.8 Hz, 1 H), 7.83 (t, J = 7.8 Hz, 1 H), 7.65 (d, J = 2.3 Hz, 1 H), 7.31 (dd, J = 8.7, 2.3 Hz, 1 H), 6.96 (d, J = 8.7 Hz, 1 H), 3.64 (t, J = 5.9 Hz, 2 H), 3.54 - 3.60 (m, 2 H), 3.06 - 3.15 (m, 4 H), 2.56 - 2.84 (m, 6 H), 2.51 - 2.55 (m, 3 H), 2.43 - 2.47 (m, 2 H), 1.43 (s, 9 H).

^{13}C NMR (101 MHz, Methanol-d_4) δ ppm 171.4, 164.2, 158.4, 157.8, 136.3, 134.1, 132.1, 130.9, 130.8, 129.5, 127.5, 127.1, 123.1, 120.6, 118.9, 80.4, 68.1, 66.3, 56.8, 52.2, 45.7, 35.8, 27.0, 12.9.

IR (neat) / cm^{-1} 1725, 1676, 1353, 1170, 1157.

HRMS (ES+) $C_{28}H_{38}N_4O_7S^{35}Cl$ requires 609.2150 found 609.2150, $C_{28}H_{38}N_4O_7S^{37}Cl$ requires 611.2120 found 611.2133.

1.2.5. Preparation of compounds **16**, **19**, **20** and **21**

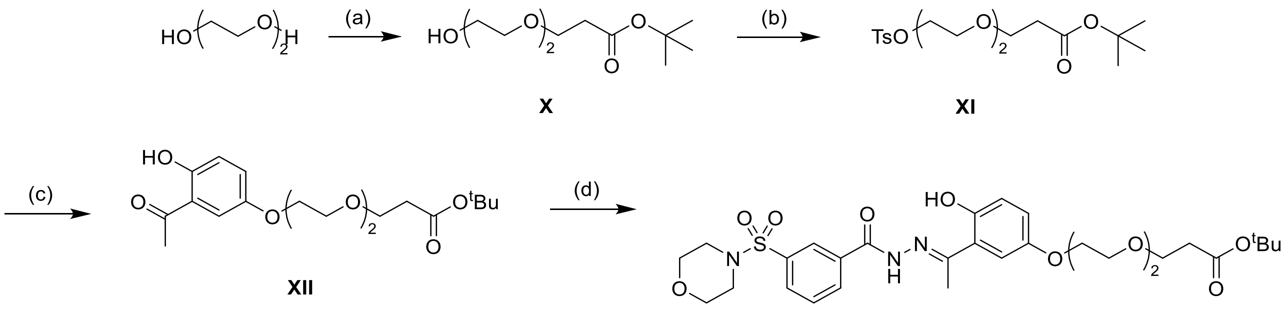

16

Reagents and conditions: (a) Tert-butyl acrylate, sodium (cat.) in dry THF, r.t., 70 hrs, 43 %; (b) Tosyl chloride, trimethylamine, DCM, r.t., 18 hrs, 82 %; (c) 2',5'-dihydroxyacetophenone, K_2CO_3, MeCN, reflux, 48 hrs, 35 %; (d) **5,** EtOH, reflux, 48 hrs, 59 %.

Tert-butyl 3-(2-(2-hydroxyethoxy)ethoxy)propanoate, X: Diethylene glycol (7.4 mL, 77.9 mmol) was added to dry THF (15 mL) and allowed to stir vigorously for ~5 mins. Sodium (20 mg, 0.87 mmol) was added and the flask was stirred for 2 hr, after which tert-butyl acrylate (2.8 mL, 19.1 mmol) was added. The flask was stirred at ambient temperature for 24 hours. The solution was neutralised to pH 7 with HCl (2 M, aq.) and extracted into EtOAc (3 x 10 mL). The organics were dried over sodium sulfate (anhyd.), filtered and solvents removed in vacuo. and purified by flash column chromatography (80 – 100 % EtOAc in n-hexanes) to give **X** as a pale yellow oil (1.97 g, 44 %).

^{1}H NMR (500 MHz, $CDCl_3$) δ ppm 3.70 - 3.76 (m, 4 H), 3.64 - 3.68 (m, 2 H), 3.59 - 3.64 (m, 4 H), 2.51 (t, J = 6.5 Hz, 2 H), 2.44 - 2.49 (m, 1 H), 1.44 (s, 9 H).

^{13}C NMR (125 MHz, $CDCl_3$) δ ppm 170.9, 80.7, 72.5, 70.4, 66.9, 61.8, 36.2, 28.1.

IR (neat) / cm^{-1} 3437, 2975, 2930, 2871, 1726, 1157, 1112, 1063.

HRMS (ES+) $C_9H_{18}O_4Na$ requires 257.1365 found 257.1376.

Spectroscopic data are consistent with previous literature.[6]

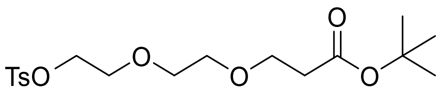

Tert-butyl 3-(2-(2-(tosyloxy)ethoxy)ethoxy)propanoate, XI: To a flask containing **X** (392 mg, 1.67 mmol) in DCM (3 mL) was added triethylamine (0.6 mL, 4.3 mmol), which was stirred for 5 mins. TsCl (384 mg, 2.01 mmol) in DCM (5 mL) was added to the flask and stirred vigorously for 66 hours. Solvent removed *in vacuo.* and the resulting suspension was purified by flash column chromatography (20 – 40 % EtOAc in petroleum ether (40-60 °C)) to yield **XI** as a colourless oil (0.423 g, 65 %).

^{1}H NMR (400 MHz, $CDCl_3$) δ ppm 7.78 - 7.82 (m, 2 H), 7.32 - 7.37 (m, 2 H), 4.14 - 4.17 (m, 2 H), 3.65 - 3.70 (m, 4 H), 3.52 - 3.59 (m, 4 H), 2.48 (t, J = 6.5 Hz, 2 H), 2.45 (s, 3 H), and 1.44 (s, 9 H).

^{13}C NMR (101 MHz, $CDCl_3$) δ ppm 170.9, 144.8, 133.0, 129.8, 128.0, 80.6, 70.7, 70.3, 69.2, 68.7, 66.9, 36.2, 28.1, 21.6.

IR (neat) / cm^{-1} 2978, 2873, 1726, 1357, 1174, 1157, 1116, 1097, 918

HRMS (ES+) $C_{18}H_{28}O_7NaS$ requires 411.1453 found 411.1449.

Spectroscopic data are consistent with previous literature.[6]

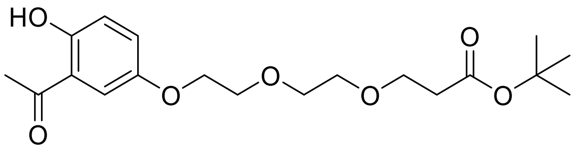

Tert-butyl 3-(2-(2-(3-acetyl-4-hydroxyphenoxy)ethoxy)ethoxy)propanoate, XII: 2′,5′-Dihydroxyacetophenone (324 mg, 2.13 mmol) and K_2CO_3 (406 mg, 2.93 mmol) were backfilled with nitrogen and refluxed in acetonitrile (10 mL) for 30 mins, after which **XI** (261 mg, 0.672 mmol) in acetonitrile (2 mL) was added, and the resulting mixture was heated at reflux for 24 hours. The solvent was removed, taken up in chloroform and filtered through celite. Chloroform solvent removed in vacuo., leaving black oils. These oils were purified by flash column chromatography (15 - 30 % EtOAc in n-hexanes) to yield **XII** as a yellow oil (110 mg, 45 %).

^{1}H NMR (400 MHz, $CDCl_3$) δ ppm 11.86 (s, 1 H), 7.25 (d, J=3.0 Hz, 1 H), 7.14 (dd, J=9.1, 3.0 Hz, 1 H), 6.92 (d, J=9.1 Hz, 1 H), 4.09 - 4.13 (m, 2 H), 3.84 - 3.88 (m, 2 H), 3.70 - 3.75 (m, 4 H), 3.63 - 3.67 (m, 2 H), 2.62 (s, 3 H), 2.49 - 2.54 (m, 2 H), 1.45 (s, 9 H).

^{13}C NMR (101 MHz, $CDCl_3$) δ ppm 204.1, 170.9, 156.9, 150.9, 124.9, 119.2, 119.1, 115.0, 80.5, 70.7, 70.4, 69.8, 68.5, 66.9, 36.3, 28.1, 26.8.

IR (neat) / cm^{-1} 2976, 2930, 2875, 1726, 1485, 1367, 1287, 1207, 1157, 1114, 1056;

HRMS (ES+) $C_{19}H_{28}O_7Na$ requires 391.1733 found 391.1733.

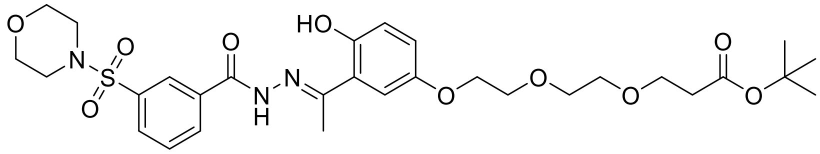

Tert-butyl 3-(2-(2-(4-hydroxy-3-(1-(2-(3-(morpholinosulfonyl)benzoyl)hydrazineylidene)ethyl) phenoxy)ethoxy)ethoxy)propanoate, 16: 5 (120 mg, 0.421 mmol) and **XII** (151 mg, 0.465 mmol) in ethanol (4.5 mL) was refluxed for 22 hours, where TLC confirmed complete conversion. The solvents were removed *in vacuo.* and the resulting solid was purified by column chromatography (50 - 100 % EtOAc in n-hexanes) to yield **16** as an amorphous pale yellow solid (156 mg, 63 %).

^{1}H NMR (400 MHz, $CDCl_3$) δ ppm 12.13 (br s, 1 H), 9.12 (br s, 1 H), 7.03 - 8.24 (m, 2 H), 7.94 (dt, J=7.98, 1.3 Hz, 1 H), 7.71 (t, J=7.79 Hz, 1 H), 7.07 (s, 1 H), 6.86 - 7.03 (m, 2 H), 4.02 (m, 2 H), 3.70 - 3.77 (m, 4 H), 3.62 - 3.70 (m, 4 H), 2.93 - 3.01 (m, 4 H), 2.47 (t, J=6.5 Hz, 2 H), 2.33 (s, 3 H), 1.38 (s, 9 H).

^{13}C NMR (101 MHz, $CDCl_3$) δ ppm 170.9, 162.5, 158.0, 153.6, 151.1, 135.8, 134.3, 132.4, 130.7, 129.8, 126.6, 119.0, 118.8, 118.5, 114.3, 80.7, 69.6, 68.4, 67.1, 66.0, 46.0, 36.3, 28.1, 13.4.

IR (neat) / cm^{-1} 3266, 1723, 1676, 1349, 1155, 1112.

HRMS (ES+) $C_{28}H_{38}N_3O_9S$ requires 592.2329 found 592.2327, $C_{28}H_{37}N_3O_9NaS$ requires 614.2148 found 614.2149.

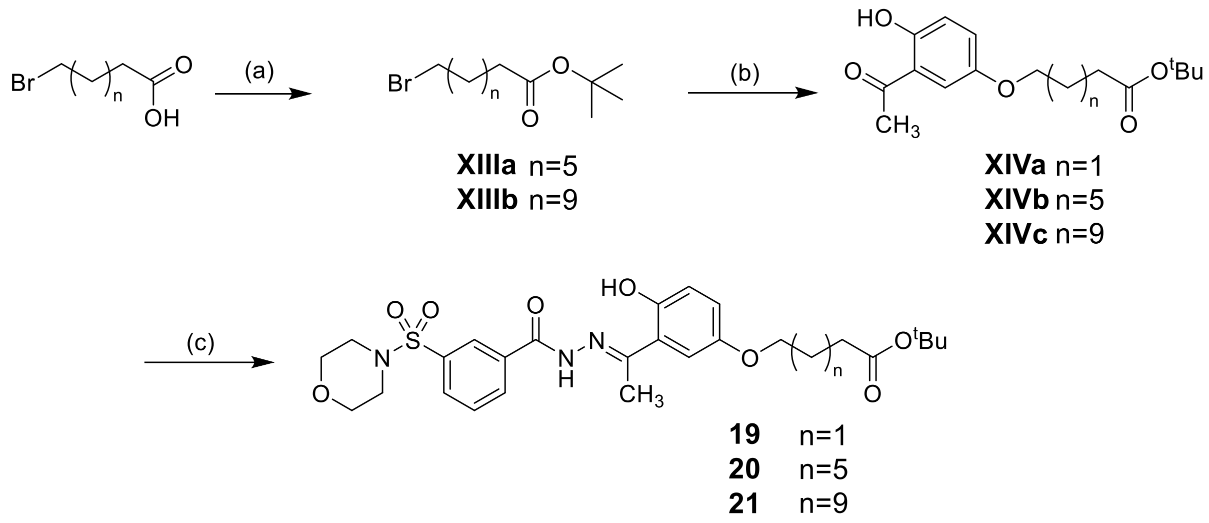

Reagents and conditions: (a) Tert-butanol, trifluoro acetic anhydride (TFAA) in dry DCM, total reaction time 6.5 hr – 24 hrs, 61 % (**XIIIa**), 63 % (**XIIIb**); (b) 2',5'-dihydroxyacetophenone, K_2CO_3, MeCN, reflux for 48 hrs, 60 % (**XIVa**), 69 % (**XIVb**), 58 % (**XIVc**); (c) **4** in EtOH, reflux for 48 hrs, 31 % (**19**), 39 % (**20**), 33 % (**21**).

General Method A for the synthesis of tert-butyl esters **XIIIa-b** from carboxylic acids

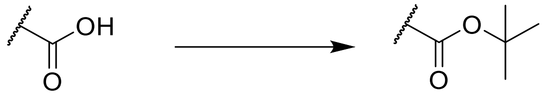

The carboxylic acid was dissolved in dry DCM and cooled in an ice bath. TFAA (~3.5 equiv.) was added and solution stirred for 3 hrs. Tert-butanol (~5 equiv.) was added and the solution stirred in an ice bath for 2 hr, then warmed and stirred at r.t. for 3 hrs. The reaction was quenched with water and extracted into EtOAc (3 times). The organic layer was dried over Na_2SO_4 (anhyd.), filtered and solvent removed *in vacuo*. The resulting crude products were purified by flash column chromatography (10 % EtOAc in n-hexanes) to yield the desired product.

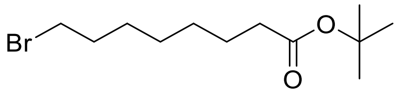

Tert-butyl 8-bromooctanoate, XIIIa: General method **A** was followed using 8-bromooctanoic acid (557 mg, 2.50 mmol), TFAA (1.2 mL, 8.63 mmol) and tert-butanol (1.2 mL, 12.6 mmol) were reacted

in DCM (5.5 mL). Work up involved quench with water (6 ml) followed by extraction into diethyl ether (3 x 15 mL). After purification, **XIIIa** was obtained as a light-brown oil (428 mg, 61 %).

^{1}H NMR (400 MHz, $CDCl_3$) δ ppm 3.41 (t, J=7.1 Hz, 2 H), 2.21 (t, J=7.1 Hz, 2 H), 1.86 (quin, J=7.1 Hz, 2 H), 1.55 - 1.63 (m, 2 H), 1.40 - 1.49 (m, 11 H), 1.28 - 1.36 (m, 4 H).

^{13}C NMR (126 MHz, $CDCl_3$) δ ppm 172.1, 78.9, 34.5, 32.8, 31.7, 27.8, 27.4, 27.1, 27.0, 23.9.

IR (neat) / cm^{-1} 2976, 2930, 2856, 1726, 1149

HRMS (ES+) mass not observed.

Spectroscopic data in line with literature.[7]

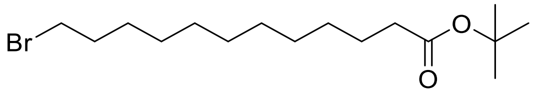

Tert-butyl 12-bromododecanoate, XIIIb: General method **A** was followed using 12-bromododecanoic acid (700 mg, 2.51 mmol), TFAA (1.06 mL, 7.52 mmol) and tert-butanol (1.15 mL, 12.5 mmol) were reacted in DCM (9 mL). Work up involved quench with water (10 mL) followed by extraction into diethyl ether (3 x 20 mL). After purification, **XIIIb** was obtained as a pale brown oil (531 mg, 63 %).

^{1}H NMR (400 MHz, $CDCl_3$) δ ppm 3.33 (t, J=7.2 Hz, 2 H), 2.12 (t, J=7.2 Hz, 1 H), 1.73 - 1.82 (m, 2 H), 1.45 - 1.55 (m, 2 H), 1.30 - 1.40 (m, 11 H), 1.17 - 1.25 (m, 12 H).

^{13}C NMR (101 MHz, $CDCl_3$) δ ppm 173.3, 79.9, 35.6, 34.0, 32.8, 29.5, 29.4, 29.4, 29.3, 29.1, 28.8, 28.2, 28.1, 25.1.

HRMS (ES+) mass not observed.

Spectroscopic data in line with literature. [8]

<u>**General Method B** for the synthesis of alkylated 2'hydroxyacetophenone derivatives **XIVa-c**</u>

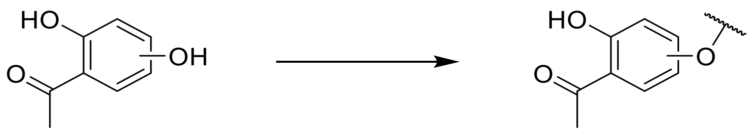

2′,5’-Dihydroxyacetophenone (~3-4 equiv.) and K_2CO_3 (1.4 equiv. relative to dihydroxyacetophenone) were backfilled with nitrogen and refluxed in acetonitrile for 30 mins, after which a terminal tert-butyl ester linker (1 equiv.) in acetonitrile (2 mL) was added, followed by reflux for the stated time. The solvent was removed, taken up in chloroform and filtered through celite. Chloroform solvent removed *in vacuo.*, leaving black oils. These oils were purified by flash column chromatography (EtOAc in n-hexanes).

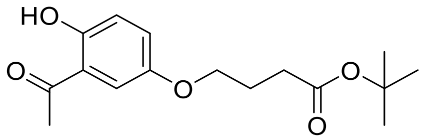

Tert-butyl 4-(3-acetyl-4-hydroxyphenoxy)butanoate, XIVa: General procedure **B** was followed using 2′,5′-Dihydroxyacetophenone (1.77 g, 11.7 mmol) and K_2CO_3 (1.93 g, 14.0 mmol) refluxed in acetonitrile (25 mL). Tert-butyl 4-bromobutanoate (1.00 mL, 5.64 mmol) added. Refluxed for 40 hrs. After workup, purified by column chromatography (10 % EtOAc in n-hexanes) to yield **XIVa** as an amorphous yellow-orange solid (989 mg, 60 %).

^{1}H NMR (400 MHz, $CDCl_3$) δ ppm 11.86 (s, 1 H), 7.20 (d, J=3.0 Hz, 1 H), 7.11 (dd, J=9.0, 3.0 Hz, 1 H), 6.92 (d, J=9.0 Hz, 1 H), 3.98 (t, J=6.7 Hz, 2 H), 2.62 (s, 3 H), 2.44 (t, J=6.7 Hz, 2 H), 2.03 - 2.11 (m, 2 H), 1.46 (s, 9 H).

^{13}C NMR (101 MHz, $CDCl_3$) δ ppm 204.1, 172.5, 156.8, 151.0, 124.8, 119.3, 119.2, 114.6, 80.5, 67.9, 31.9, 28.1, 26.8, 24.7.

IR (neat) / cm^{-1} 2972, 2924, 2885, 1724, 1365, 1297, 1205, 1153, 1037;

HRMS (ES-) $C_{16}H_{21}O_5$ requires 293.1389 found 293.1389.

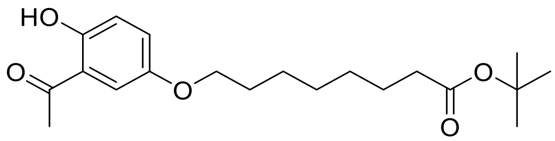

Tert-butyl 8-(3-acetyl-4-hydroxyphenoxy)octanoate, XIVb: General procedure **B** was followed using 2′,5′-Dihydroxyacetophenone (495 mg, 3.26 mmol) and K_2CO_3 (630 mg, 4.56 mmol) refluxed in acetonitrile (10 mL). Compound **XIIIa** (620 mg, 2.22 mmol) in acetonitrile (5 mL) added. Refluxed

for 40 hrs. After workup, purified by column chromatography (3 – 10 % EtOAc in n-hexanes) to yield **XIVb** as an amorphous green solid (540 mg, 69 %).

^{1}H NMR (400 MHz, $CDCl_3$) δ ppm 11.84 (s, 1 H), 7.18 (d, J=2.9 Hz, 1 H), 7.11 (dd, J=9.1, 3.0 Hz, 1 H), 6.92 (d, J=9.1 Hz, 1 H), 3.93 (t, J=6.5 Hz, 2 H), 2.62 (s, 3 H), 2.22 (t, J=7.5 Hz, 2 H), 1.73 - 1.82 (m, 2 H), 1.56 - 1.65 (m, 2 H), 1.43 - 1.51 (m, 11 H), 1.32 - 1.42 (m, 4 H).

^{13}C NMR (101 MHz, $CDCl_3$) δ ppm 204.1, 173.2, 156.7, 151.2, 124.7, 119.3, 119.1, 114.6, 29.3, 29.1, 29.0, 28.1, 26.8, 25.9, 25.0.

IR (neat) / cm^{-1} 2976, 2932, 2858, 1726, 1486, 1365, 1287, 1209, 1147.

HRMS (ES+) $C_{20}H_{31}O_5$ requires 351.2171 found 351.2169, $C_{20}H_{30}O_5Na$ requires 373.1992 found 373.1992.

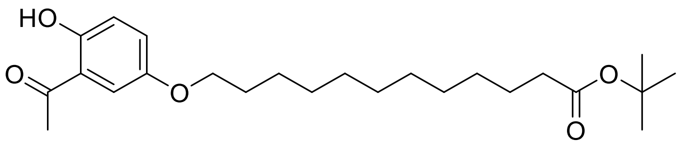

Tert-butyl 12-(3-acetyl-4-hydroxyphenoxy)dodecanoate, XIVc: General procedure **B** was followed using 2′,5′-Dihydroxyacetophenone (945 mg, 6.22 mmol) and K_2CO_3 (1.08 g, 7.82 mmol) refluxed in acetonitrile (9 mL). Compound **XIIIb** (825 mg, 2.47 mmol) in acetonitrile (13 mL) added. Refluxed for 48 hrs. After workup, purified by column chromatography (5 – 8 % EtOAc in n-hexanes) to yield **XIVc** as an orange oil (584 mg, 58 %).

^{1}H NMR (400 MHz, $CDCl_3$) δ ppm 11.85 (s, 1 H), 7.19 (d, J=3.0 Hz, 1 H), 7.12 (dd, J=9.1, 3.0 Hz, 1 H), 6.92 (d, J=9.1 Hz, 1 H), 3.93 (t, J=6.9 Hz, 2 H), 2.62 (s, 3 H), 2.21 (t, J=6.9 Hz, 2 H), 1.78 (quin, J=6.9 Hz, 2 H), 1.54 - 1.66 (m, 2 H), 1.44 - 1.46 (m, 11 H), 1.26 - 1.36 (m, 12 H).

^{13}C NMR (101 MHz, $CDCl_3$) δ ppm 204.1, 173.3, 156.6, 151.2, 124.7, 119.3, 119.1, 114.6, 79.9, 69.0, 35.6, 29.53, 29.52, 29.45, 29.39, 29.34, 29.30, 29.1, 28.1, 26.8, 26.0, 25.1.

IR (neat) / cm^{-1} 2976, 2924, 2854, 1728, 1487, 1365, 1287, 1209, 1149.

HRMS (ES- Direct Infusion) $C_{24}H_{37}O_5$ requires 405.2641 found 405.2641.

General Method C for the synthesis of hydrazones **21-23**

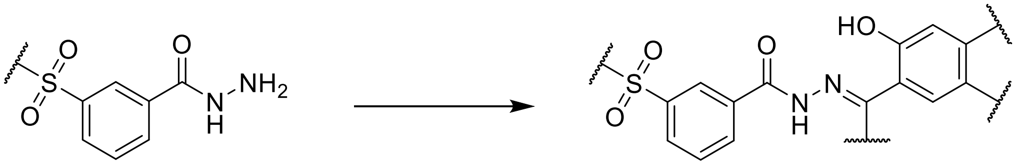

Compound **4** (1 equiv.) and ketone (1.1 equiv.) were dissolved in ethanol (3-6 mL) and refluxed for 20-48 hr, where TLC confirmed complete conversion. The solvents were removed *in vacuo.* and the resulting solid was purified by column chromatography to yield the desired hydrazones.

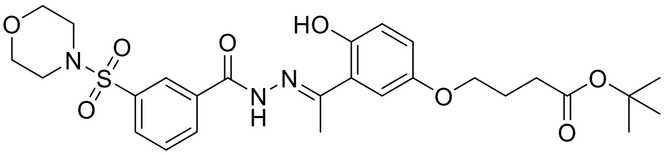

Tert-butyl 4-(4-hydroxy-3-(1-(2-(3-(morpholinosulfonyl)benzoyl)hydrazineylidene)ethyl) phenoxy)butanoate, 19: General method C was followed using compound **5** (236 mg, 0.826 mmol) and **XIVa** (270 mg, 0.917 mmol) in ethanol (3 mL), refluxed for 48 hr, purified by column chromatography (30 – 100 % EtOAc in n-hexanes) to yield **19** as an amorphous brown solid (171 mg, 36 %).

^{1}H NMR (400 MHz, $CDCl_3$) δ ppm 12.09 (br s, 1 H), 9.05 (br s, 1 H), 8.00 - 8.23 (m, 2 H), 7.94 (d, J=7.9 Hz, 1 H), 7.71 (t, J=7.9 Hz, 1 H), 6.81 - 7.07 (m, 3 H), 3.97 (br t, J=6.4 Hz, 2 H), 3.74 (app. br s, 4 H), 2.98 - 3.12 (m, 4 H), 2.37 - 2.47 (m, 5 H), 2.05 (quin, J=6.4 Hz, 2 H), 1.46 (s, 9 H).

^{13}C NMR (101 MHz, $CDCl_3$) δ ppm 172.6, 162.3, 157.8, 153.5, 151.2, 136.0, 134.3, 132.3, 130.8, 129.9, 126.5, 118.9, 118.8, 118.7, 113.9, 80.4, 67.9, 66.0, 46.0, 32.0, 28.1, 24.9, 13.2.

IR (neat) / cm^{-1} 3330, 1717, 1678, 1407, 1344, 1256, 1166, 1149, 1112.

HRMS (ES+) $C_{27}H_{36}N_3O_8S$ requires 562.2223 found 562.2224.

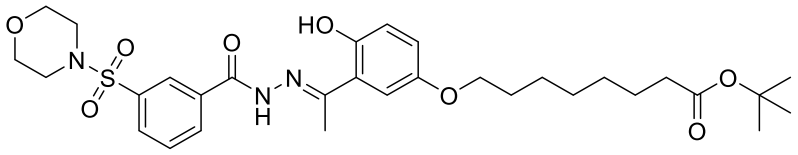

Tert-butyl 8-(4-hydroxy-3-(1-(2-(3-(morpholinosulfonyl)benzoyl)hydrazineylidene)ethyl) phenoxy)octanoate, 20: General method **C** was followed using compound **5** (200 mg, 0.701 mmol) and **XIVb** (223 mg, 0.637 mmol) in ethanol (9 mL), refluxed for 48 hr, purified by column chromatography (50 – 100 % EtOAc in n-hexanes) to yield **20** as an amorphous beige solid (171 mg, 39 %).

^{1}H NMR (400 MHz, $CDCl_3$) δ ppm 12.08 (br s, 1 H), 9.08 (br s, 1 H), 7.97 - 8.25 (m, 2 H), 7.93 (br d, J=7.7 Hz, 1 H), 7.71 (t, J=7.7 Hz, 1 H), 6.76 - 7.05 (m, 3 H), 3.92 (br t, J=6.5 Hz, 2 H), 3.74 (br s, 4 H), 3.04 (br s, 4 H), 2.41 (s, 3 H), 2.21 (t, J=7.5 Hz, 2 H), 1.72 - 1.81 (m, 2 H), 1.55 - 1.64 (m, 2 H), 1.44 (s, 11 H), 1.31 - 1.40 (m, 4 H).

^{13}C NMR (101 MHz, $CDCl_3$) δ ppm 173.3, 162.2, 157.8, 153.4, 151.5, 136.1, 134.3, 132.3, 130.8, 129.9, 126.5, 118.9, 118.4 - 118.8, 113.9, 80.0, 68.9, 66.0, 46.0, 35.6, 29.4, 29.1, 29.0, 28.1, 25.9, 25.0, 13.2.

IR (neat) / cm^{-1} 3311, 1728, 1678, 1493, 1234, 1219, 1149, 1110.

HRMS (ES+) $C_{31}H_{44}N_3O_8S$ requires 618.2849 found 618.2850, $C_{31}H_{43}N_3O_8SNa$ requires 640.2669 found 640.2674.

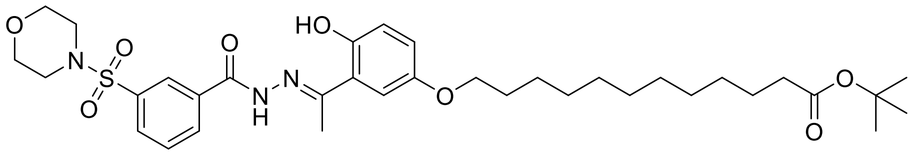

Tert-butyl 12-(4-hydroxy-3-(1-(2-(3-(morpholinosulfonyl)benzoyl)hydrazineylidene)ethyl) phenoxy)dodecanoate, 21: General method **C** was followed using compound **5** (250 mg, 0.876 mmol) and **XIVc** (404 mg, 0.994 mmol) in ethanol (8 mL), refluxed for 48 hr, purified by column chromatography (15 – 30 % EtOAc in n-hexanes) to yield **21** as an amorphous yellow solid (191 mg, 33 %).

^{1}H NMR (400 MHz, $CDCl_3$) δ ppm 12.17 (br s, 1 H), 9.32 (br s, 1 H), 7.95 - 8.23 (m, 2 H), 7.90 (br d, J=7.6 Hz, 1 H), 7.68 (br t, J=7.6 Hz, 1 H), 6.66 - 7.05 (m, 3 H), 3.92 (br t, J=6.4 Hz, 2 H), 3.64 - 3.82 (m, 4 H), 2.97 - 3.09 (m, 4 H), 2.42 (s, 3 H), 2.20 (t, J=7.5 Hz, 2 H), 1.77 (quin, J=7.0 Hz, 2 H), 1.53 - 1.61 (m, 2 H), 1.40 - 1.52 (m, 11 H), 1.26 - 1.39 (m, 12 H).

^{13}C NMR (101 MHz, $CDCl_3$) δ ppm 173.4, 162.6, 158.1, 153.3, 151.5, 135.8, 134.3, 132.4, 130.7, 129.8, 126.6, 119.0, 118.6, 118.5, 113.9, 80.0, 69.0, 66.0, 46.0, 35.6, 29.55, 29.53, 29.46, 29.43, 29.3, 29.1, 28.1, 26.1, 25.1, 13.4.

IR (neat) / cm^{-1} 3272, 1728, 1678, 1490, 1351, 1260, 1168, 1151, 1112.

HRMS (ES+) $C_{35}H_{52}N_3O_8S$ requires 674.3475 found 674.3480.

1.2.6 Synthesis of **25-33**

General Method D for the synthesis of **25-33**

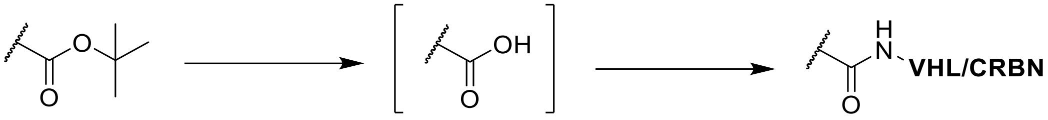

A solution of NaOH in bench MeOH (4 M) was added to tert-butyl protected inhibitors (~0.3 mmol) dissolved in bench DCM, making a final 0.4 M NaOH in DCM/MeOH (9:1, v/v). The flasks were flushed with nitrogen and reacted at r.t. for 24 hr, after which the solvent was removed *in vacuo*. The mixture was then taken up in water and HCl (2 M, aq.) was added dropwise until pH paper indicated pH 2 had been reached; creamy suspensions were observed. The product was extracted into ethyl acetate 3 times and washed with brine. The organics were dried over sodium sulfate (anhyd.), filtered and solvent removed *in vacuo.* to leave a brown oil. These oils were purified on silica through column chromatography to obtain the desired products, which were further dried by crashing out from methanol to obtain beige solids.

These carboxylic acids were then dissolved in DMF and DIPEA (5 equiv.), cooled with an ice bath. To these mixtures was added a solution of HATU (1.1-2 equiv.) in DMF. After 15 mins, a solution of E3 ligand (1.2-1.4 equiv.) in DMF was added and the solution was stirred at r.t. for 24 hrs. The solution was diluted with ethyl acetate (5 mL) and washed with brine (3 x 5 mL). The aqueous layer was further extracted into ethyl acetate (5-10 mL) and then the organics were combined and dried over sodium sulfate (anhyd.), filtered and solvent removed *in vacuo.*, to leave a brown oil. The brown oils were purified on silica (MeOH in EtOAc eluents) and then further purified by semi-prep RP-HPLC (30 minute gradients) to obtain the desired compounds.

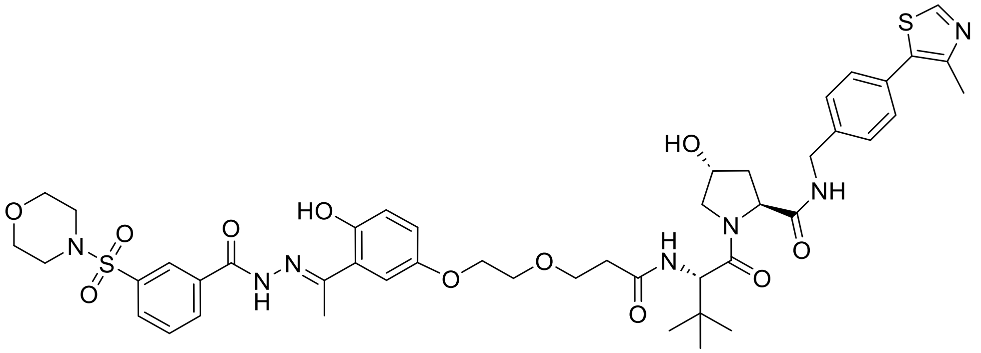

(2S,4R)-4-hydroxy-1-((S)-2-(3-(2-(4-hydroxy-3-(1-(2-(3-(morpholinosulfonyl)benzoyl)hydrazineylidene)ethyl)phenoxy)ethoxy)propanamido)-3,3-dimethylbutanoyl)-N-(4-(4-methylthiazol-5-yl)benzyl)pyrrolidine-2-carboxamide, 25: General method **D** was followed using **13** (162 mg, 0.274 mmol) was reacted in 0.4 M NaOH solution (8 mL). Workup involved dissolution in water (10 mL), extraction into EtOAc (3 x 10 mL). Organics washed with brine (10 mL), dried over sodium sulfate (anhyd.), filtered and the solvent removed *in vacuo*. The resulting oil purified by column chromatography (4 – 10 % MeOH in DCM) to yield **17** as an amorphous beige solid (89.2 mg, 61 %).

17 (40.0 mg, 74.7 µmol), DIPEA (67.0 µL, 383 µmol), HATU (58.4 mg, 154 µmol) and **VHL-NH$_2$** (44.0 mg, 87.4 µmol) were then directly reacted in DMF (1.0 mL). After workup, the oil was purified on silica gel by column chromatography (3-10 % MeOH in EtOAc) followed by semi-prep RP-HPLC (45 – 65 % MeCN in H_2O, t_R = 14.6 mins). After lyophilising, **25** was yielded as a white solid (4.5 mg, 6 %).

^{1}H NMR (400 MHz, Methanol-*d*$_4$) δ ppm 8.77 (br s, 1 H), 8.21 (s, 1 H), 8.15 (br d, J=7.7 Hz, 1 H), 7.90 (br d, J=7.7 Hz, 1 H), 7.70 (t, J=7.7 Hz, 1 H), 7.34 - 7.49 (m, 4 H), 7.09 (br d, J=2.4 Hz, 1 H), 6.88 (br dd, J=8.8, 2.4 Hz, 1 H), 6.76 (d, J=8.8 Hz, 1 H), 4.56 (s, 1 H), 4.35 - 4.50 (m, 3 H), 4.25 (d, J=15.5 Hz, 1 H), 4.03 (br t, J=4.5 Hz, 2 H), 3.67 - 3.81 (m, 6 H), 3.59 - 3.66 (m, 4 H), 2.88 - 2.98 (m, 4 H), 2.34 - 2.56 (m, 8 H), 2.07 - 2.15 (m, 1 H), 1.93 - 2.02 (m, 1 H), 0.93 (s, 9 H). 5 exchangeable protons not observed.

HRMS (ES+) $C_{46}H_{58}N_7O_{11}S_2$ requires 948.3636 found 948.3646, $C_{46}H_{57}N_7O_{11}S_2Na$ requires 970.3455 found 970.3444.

HPLC t_R = 27.8 mins (5 – 100 % MeCN in H_2O, 0.1 % TFA, 30 min gradient), 95 % purity.

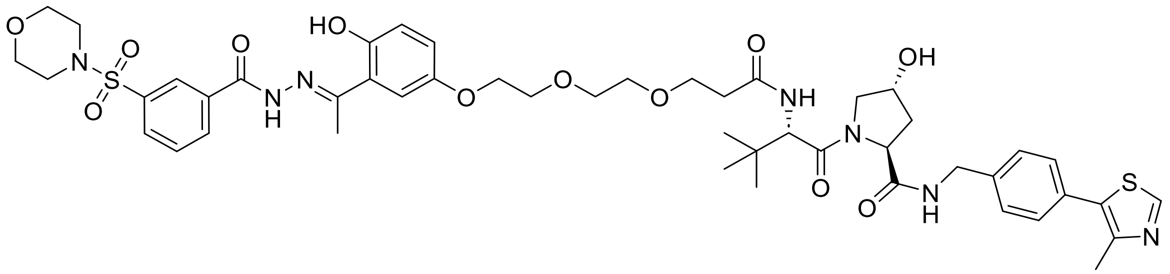

(2S,4R)-4-hydroxy-1-((S)-2-(3-(2-(2-(4-hydroxy-3-(-1-(2-(3-(morpholinosulfonyl)benzoyl)hydrazineylidene)ethyl)phenoxy)ethoxy)ethoxy)propanamido)-3,3-dimethylbutanoyl)-N-(4-(4-methylthiazol-5-yl)benzyl)pyrrolidine-2-carboxamide, 26: General method **D** was followed using **16** (170 mg, 0.267 mmol) was reacted in 0.4 M NaOH solution (10 mL). Workup involved dissolution in water (10 mL), extraction into EtOAc (3 x 10 mL). Organics washed with brine (10 mL), dried over sodium sulfate (anhyd.), filtered and the solvent removed *in vacuo*. The resulting oil purified by column chromatography (4 – 10 % MeOH in DCM) to yield **18** as an amorphous beige solid (97.6 mg, 63 %).

18 (41.0 mg, 70.7 µmol), DIPEA (61.6 µL, 354 µmol), HATU (29.6 mg, 77.8 µmol) and **VHL-NH$_2$** (42.7 mg, 84.9 µmol) were then reacted in DMF (0.85 mL). After workup, the oil was purified on silica gel by column chromatography (10 % MeOH in EtOAc) followed by semi-prep RP-HPLC (50 – 58 % MeCN in H_2O, t_R = 12.8 mins). After lyophilising, **26** was yielded as a white solid (6.3 mg, 9 %).

^{1}H NMR (400 MHz, Methanol-*d*$_4$) δ ppm 8.86 (s, 1 H), 8.60 (t, J=5.2 Hz, 1 H), 8.30 (s, 1 H), 8.24 (br d, J=7.7 Hz, 1 H), 8.01 (br d, J=7.7 Hz, 1 H), 7.89 (br d, J=9.1 Hz, 1 H), 7.80 (t, J=7.7 Hz, 1 H), 7.36 - 7.48 (m, 4 H), 7.18 (d, J=2.6 Hz, 1 H), 6.97 (dd, J=8.8, 2.6 Hz, 1 H), 6.87 (d, J=8.8 Hz, 1 H), 4.63 - 4.67 (m, 1 H), 4.44 - 4.59 (m, 3 H), 4.34 (dd, J=15.4, 5.2 Hz, 1 H), 4.11 (t, J=4.7 Hz, 2 H), 3.88 (d, J=10.9 Hz, 1 H), 3.61 - 3.83 (m, 13 H), 2.96 - 3.09 (m, 4 H), 2.42 - 2.64 (m, 8 H), 2.16 - 2.25 (m, 1 H), 2.04 - 2.12 (m, 1 H), 1.03 (s, 9 H). 3 exchangeable protons not observed.

HRMS (ES+) $C_{46}H_{62}N_7O_{12}S_2$ requires 992.3898 found 992.3890, $C_{48}H_{61}N_7O_{12}S_2Na$ requires 1014.3717 found 1014.3704.

HPLC t_R = 28.2 mins (5 – 100 % MeCN in H_2O, 0.1 % TFA, 30 min gradient), 95 % purity.

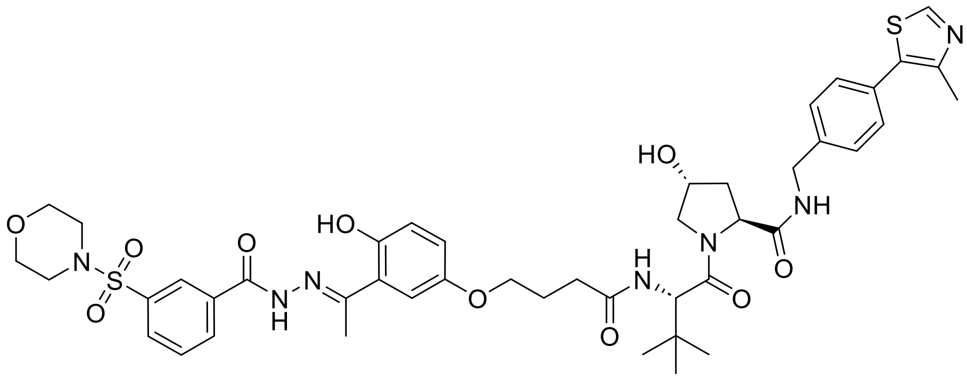

(2S,4R)-4-hydroxy-1-((S)-2-(4-(4-hydroxy-3-(1-(2-(3-(morpholinosulfonyl)benzoyl)hydrazineylidene)ethyl)phenoxy)butanamido)-3,3-dimethylbutanoyl)-N-(4-(4-methylthiazol-5-yl)benzyl)pyrrolidine-2-carboxamide, 27: General method **D** was followed using **19** (131 mg, 0.232 mmol) was reacted in 0.4 M NaOH solution (7 mL). Workup involved dissolution in water (10 mL), extraction into EtOAc (3 x 20 mL). Organics washed with brine (10 mL), dried over sodium sulfate (anhyd.), filtered and the solvent removed *in vacuo*. The resulting oil purified by column chromatography (3 – 5 % MeOH in DCM) to yield **22** as an amorphous beige solid (41.0 mg, 35 %).

22 (40.0 mg, 79.1 µmol), DIPEA (69.0 µL, 395 µmol), HATU (33.1 mg, 87.1 µmol) and **VHL-NH$_2$** (51.8 mg, 103 µmol) were then reacted in DMF (0.85 mL). After workup, the oil was purified on silica gel by column chromatography (7 % MeOH in EtOAc) followed by semi-prep RP-HPLC (45 – 60 % MeCN in H_2O, t_R = 17.5 mins). After lyophilising, **27** was yielded as a white solid (4.2 mg, 6 %).

^{1}H NMR (400 MHz, Methanol-d_4) δ ppm 8.87 (br s, 1 H), 8.31 (s, 1 H), 8.25 (br d, J=7.8 Hz, 1 H), 7.98 (br d, J=7.8 Hz, 1 H), 7.79 (t, J=7.75 Hz, 1 H), 7.36 - 7.49 (m, 4 H), 7.17 (br d, J=2.4 Hz, 1 H), 6.96 (br dd, J=8.9, 2.4 Hz, 1 H), 6.86 (br d, J=8.9 Hz, 1 H), 4.65 (s, 1 H), 4.46 - 4.60 (m, 3 H), 4.36 (d, J=15.4 Hz, 1 H), 3.99 (br t, J=6.2 Hz, 2 H), 3.91 (d, J=10.9 Hz, 1 H), 3.81 (dd, J=10.9, 3.9 Hz, 1 H), 3.69 - 3.74 (m, 4 H), 2.99 - 3.05 (m, 4 H), 2.44 - 2.54 (m, 8 H), 2.17 - 2.25 (m, 1 H), 2.00 - 2.14 (m, 3 H), 1.03 (s, 9 H). 5 exchangeable protons not observed.

HRMS (ES+) $C_{45}H_{56}N_7O_{10}S_2$ requires 918.3530 found 918.3515, $C_{45}H_{55}N_7O_{10}S_2Na$ requires 940.3350 found 940.3333.

HPLC t_R = 28.4 mins (5 – 100 % MeCN in H_2O, 0.1 % TFA, 30 min gradient), 95 % purity.

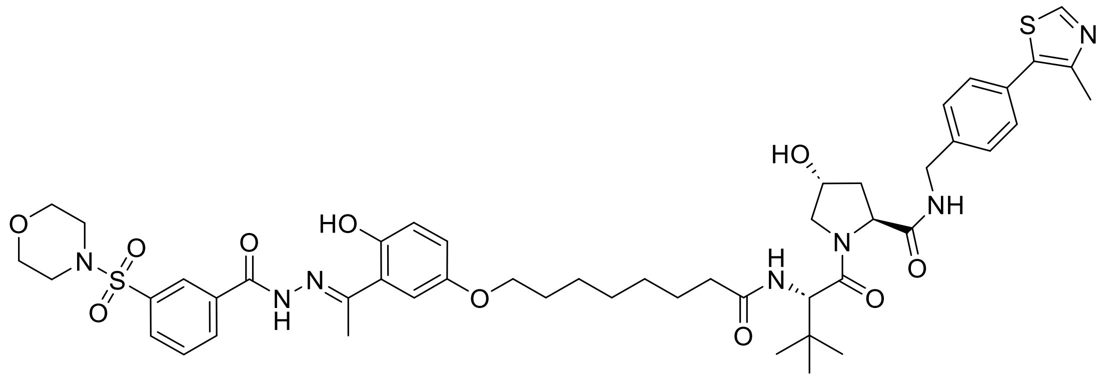

(2S,4R)-4-hydroxy-1-((S)-2-(8-(4-hydroxy-3-(1-(2-(3-(morpholinosulfonyl)benzoyl)hydrazineylidene)ethyl)phenoxy)octanamido)-3,3-dimethylbutanoyl)-N-(4-(4-methylthiazol-5-yl)benzyl)pyrrolidine-2-carboxamide, 28: General method **D** was followed using **20** (171 mg, 0.277 mmol) was reacted in 0.4 M NaOH solution (10 mL). Workup involved dissolution in water (14 mL), extraction into EtOAc (3 x 20 mL). Organics washed with brine (10 mL), dried over sodium sulfate (anhyd.), filtered and the solvent removed *in vacuo*. The resulting oil purified by column chromatography (2 – 6 % MeOH in DCM) to yield **23** as an amorphous beige solid (108 mg, 69 %), silica gel TLC R_f 0.36 (10 % MeOH in DCM).

23 (42.0 mg, 74.8 µmol), DIPEA (67 µL, 383 µmol), HATU (34.0 mg, 89.7 µmol) and **VHL-NH$_2$** (52.7 mg, 105 µmol) were then reacted in DMF (1.2 mL). After workup, the oil was purified on silica gel by column chromatography (6 % MeOH in EtOAc) followed by semi-prep RP-HPLC (55 – 75 % MeCN in H_2O, t_R = 15.5 mins). After lyophilising, **28** was yielded as a white solid (4.8 mg, 7 %).

^{1}H NMR (400 MHz, Methanol-d_4) δ ppm 8.87 (s, 1 H), 8.31 (s, 1 H), 8.25 (br d, J=7.6 Hz, 1 H), 7.99 (br d, J=7.6 Hz, 1 H), 7.79 (t, J=7.6 Hz, 1 H), 7.37 - 7.49 (m, 4 H), 7.13 (dd, J=2.5 Hz, 1 H), 6.94 (dd, J=8.9, 2.5 Hz, 1 H), 6.86 (d, J=8.9 Hz, 1 H), 4.64 (s, 1 H), 4.46 - 4.61 (m, 3 H), 4.35 (d, J=15.5 Hz, 1 H), 3.86 - 4.03 (m, 3 H), 3.80 (dd, J=11.0, 3.9 Hz, 1 H), 3.67 - 3.75 (m, 4 H), 2.99 - 3.07 (m, 4 H), 2.43 - 2.53 (m, 6 H), 2.18 - 2.36 (m, 2 H), 2.04 - 2.11 (m, 1 H), 1.71 - 1.81 (m, 2 H), 1.57 - 1.70 (m, 2 H), 1.45 - 1.54 (m, 2 H), 1.34 - 1.43 (m, 4 H), 1.04 (s, 9 H). 5 exchangeable protons not observed.

HRMS (ES+) $C_{49}H_{64}N_7O_{10}S_2$ requires 974.4156 found 974.4132, $C_{49}H_{63}N_7O_{10}S_2Na$ requires 996.3976 found 996.3931.

HPLC t_R = 31.3 mins (5 – 100 % MeCN in H_2O, 0.1 % TFA, 30 min gradient), 99 % purity.

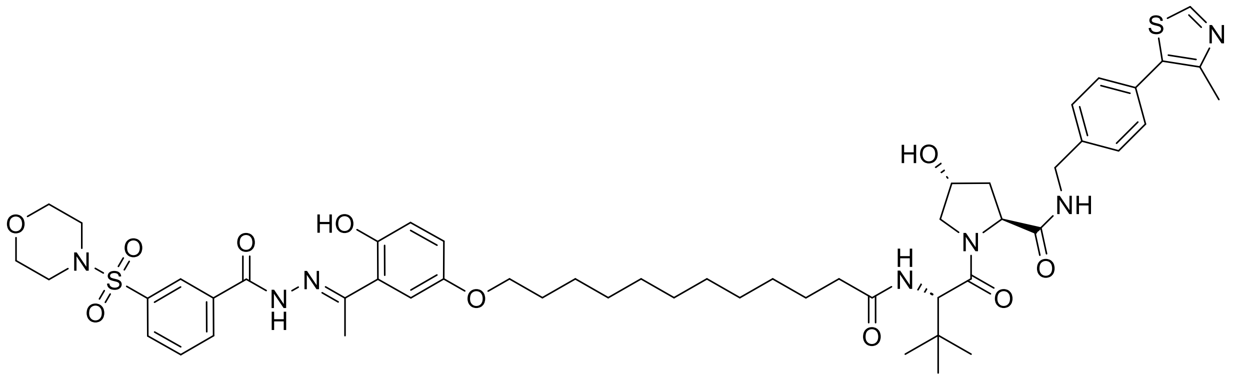

(2S,4R)-4-hydroxy-1-((S)-2-(12-(4-hydroxy-3-(1-(2-(3-(morpholinosulfonyl)benzoyl)hydrazineylidene)ethyl)phenoxy)dodecanamido)-3,3-dimethylbutanoyl)-N-(4-(4-methylthiazol-5-yl)benzyl)pyrrolidine-2-carboxamide, 29: General method **D** was followed using **21** (190 mg, 0.232 mmol) was reacted in 0.4 M NaOH solution (10 mL). Workup involved dissolution in water (15 mL), extraction into EtOAc (3 x 15 mL). Organics washed with brine (10 mL), dried over sodium sulfate (anhyd.), filtered and the solvent removed *in vacuo*. The resulting oil purified by column chromatography (3 – 4 % MeOH in DCM) to yield **24** as an amorphous beige solid (71.1 mg, 41 %).

24 (38.0 mg, 61.5 µmol), DIPEA (55.0 µL, 307 µmol), HATU (46.6 mg, 123 µmol) and **VHL-NH$_2$** (38.4 mg, 76.2 µmol) were then reacted in DMF (1.0 mL). After workup, the oil was purified on silica gel by column chromatography (3 % MeOH in EtOAc) followed by semi-prep RP-HPLC (45 – 70 % MeCN in H_2O, t_R = 33.3 mins). After lyophilising, **29** was yielded as a white solid (2.0 mg, 3 %).

^{1}H NMR (400 MHz, Methanol-d$_4$) δ ppm 8.87 (s, 1 H), 8.30 (s, 1 H), 8.24 (br d, J=7.8 Hz, 1 H), 8.01 (br d, J=7.8 Hz, 1 H), 7.78 - 7.83 (m, 2 H), 7.38 - 7.51 (m, 4 H), 7.13 (br d, J=2.2 Hz, 1 H), 6.95 (dd, J=8.8, 2.2 Hz, 1 H), 6.87 (d, J=8.8 Hz, 1 H), 4.63 (br d, J=9.1 Hz, 1 H), 4.47 - 4.56 (m, 3 H), 4.35 (d, J=15.5 Hz, 1 H), 3.96 (br t, J=6.3 Hz, 2 H), 3.90 (br d, J=11.0 Hz, 1 H), 3.80 (dd, J=11.0, 3.9 Hz, 1 H), 3.69 - 3.75 (m, 4 H), 2.98 - 3.06 (m, 4 H), 2.44 - 2.52 (m, 6 H), 2.17 - 2.34 (m, 3 H), 2.03 - 2.13 (m, 1 H), 1.71 - 1.81 (m, 2 H), 1.55 - 1.66 (m, 2 H), 1.44 - 1.53 (m, 2 H), 1.29 - 1.41 (m, 12 H), 1.03 (s, 9 H). 4 exchangeable protons not observed.

HRMS (ES+) $C_{53}H_{72}N_7O_{10}S_2$ $[M+H]^+$ requires 1030.4782 found 1030.4751.

HPLC t_R = 35.3 mins (5 – 100 % MeCN in H_2O, 0.1 % TFA, 30 min gradient), 97 % purity.

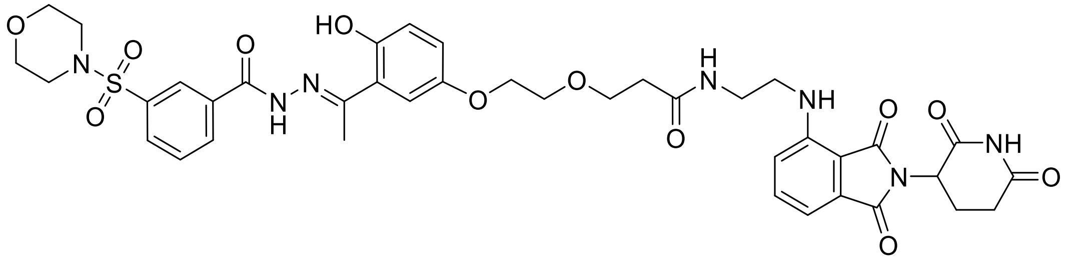

***N*-(2-((2-(2,6-dioxopiperidin-3-yl)-1,3-dioxoisoindolin-4-yl)amino)ethyl)-3-(2-(4-hydroxy-3-(1-(2-(3-(morpholinosulfonyl)benzoyl)hydrazineylidene)ethyl)phenoxy)ethoxy)propanamide, 30:** The amide coupling portion of general procedure **D** was followed using **17** (45.0 mg, 84.0 µmol), DIPEA (73.0 µL, 418 µmol), HATU (38.6 mg, 102 µmol) and **CRBN-NH$_2$** (42.2 mg, 120 µmol) were reacted in DMF (1.05 mL). After workup, the oil was purified on silica gel by column chromatography (8 % MeOH in EtOAc) followed by RP HPLC (50 – 58 % MeCN in H_2O, t_R = 10.8 mins). After lyophilising, **30** was yielded as a green solid (6.5 mg, 9 %).

^{1}H NMR (400 MHz, Methanol-*d*$_4$) δ ppm 8.29 (s, 1 H), 8.22 (br d, J=7.8 Hz, 1 H), 7.99 (br d, J=7.8 Hz, 1 H), 7.79 (t, J=7.8 Hz, 1 H), 7.46 (dd, J=8.5, 7.2 Hz, 1 H), 7.08 (d, J=2.8 Hz, 1 H), 6.95 - 7.02 (m, 2 H), 6.92 (dd, J=8.8, 2.8 Hz, 1 H), 6.81 (d, J=8.8 Hz, 1 H), 4.99 (dd, J=12.4, 5.5 Hz, 1 H), 4.01 - 4.11 (m, 2 H), 3.75 - 3.83 (m, 4 H), 3.66 - 3.75 (m, 4 H), 3.34 - 3.43 (m, 4 H), 2.96 - 3.11 (m, 4 H), 2.63 - 2.87 (m, 3 H), 2.47 (t, J=5.9 Hz, 2 H), 2.39 (s, 3 H), 2.04 - 2.13 (m, 1 H). 5 exchangeable protons not observed.

HRMS (ES+) $C_{39}H_{44}N_7O_{12}S$ requires 834.2769 found 834.2766, $C_{39}H_{43}N_7O_{12}SNa$ requires 856.2588found 856.2587.

HPLC t_R = 28.1 mins (5 – 100 % MeCN in H_2O, 0.1 % TFA, 30 min gradient), 97 % purity.

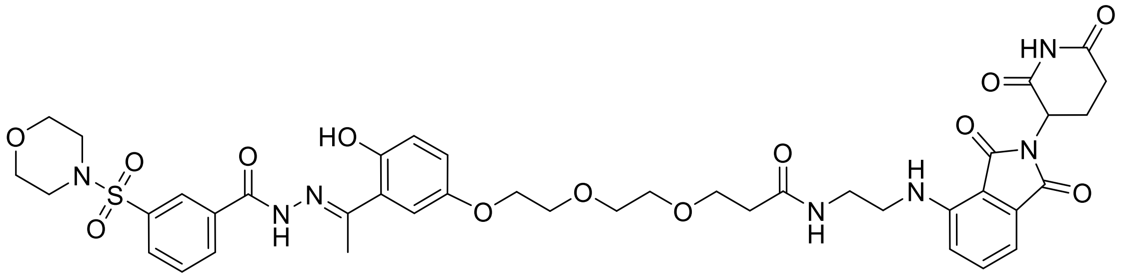

***N*-(2-((2-(2,6-dioxopiperidin-3-yl)-1,3-dioxoisoindolin-4-yl)amino)ethyl)-3-(2-(2-(4-hydroxy-3-(1-(2-(3-(morpholinosulfonyl)benzoyl)hydrazineylidene)ethyl)phenoxy)ethoxy)ethoxy) propanamide, 31:** The amide coupling portion of general procedure **D** was followed using **18** (49.8 mg, 85.9 µmol), DIPEA (75.0 µL, 431 µmol), HATU (53.1 mg, 140 µmol) and **CRBN-NH$_2$** (45.7 mg, 130 µmol) were reacted in DMF (1.08 mL). After workup, the oil was purified on silica gel by column chromatography (10 % MeOH in EtOAc) followed by RP HPLC (50 – 60 % MeCN in H_2O, t_R = 10.4 mins). After lyophilising, **31** was yielded as a green solid (4.5 mg, 6 %).

^{1}H NMR (400 MHz, Methanol-*d*$_4$) δ ppm 8.29 (s, 1 H), 8.23 (br d, J=7.9 Hz, 1 H), 8.00 (br d, J=7.9 Hz, 1 H), 7.80 (t, J=7.9 Hz, 1 H), 7.48 (dd, J=8.5, 7.2 Hz, 1 H), 7.08 (d, J=2.8 Hz, 1 H), 6.97 - 7.03 (m, 2 H), 6.93 (dd, J=8.9, 2.8 Hz, 1 H), 6.82 (d, J=8.9 Hz, 1 H), 5.01 (dd, J=12.5, 5.4 Hz, 1 H), 4.02 - 4.10 (m, 2 H), 3.77 - 3.82 (m, 2 H), 3.61 - 3.75 (m, 10 H), 3.34 - 3.40 (m, 4 H), 3.00 - 3.08 (m, 4 H), 2.61 - 2.88 (m, 3 H), 2.36 - 2.48 (m, 5 H), 2.08 - 2.17 (m, 1 H). 5 exchangeable protons not observed.

HRMS (ES+) $C_{41}H_{48}N_7O_{13}S$ requires 878.3031 found 878.3029, $C_{41}H_{47}N_7O_{13}SNa$ requires 900.2850 found 900.2853.

HPLC t_R = 28.2 mins (5 – 100 % MeCN in H_2O, 0.1 % TFA, 30 min gradient), 96 % purity.

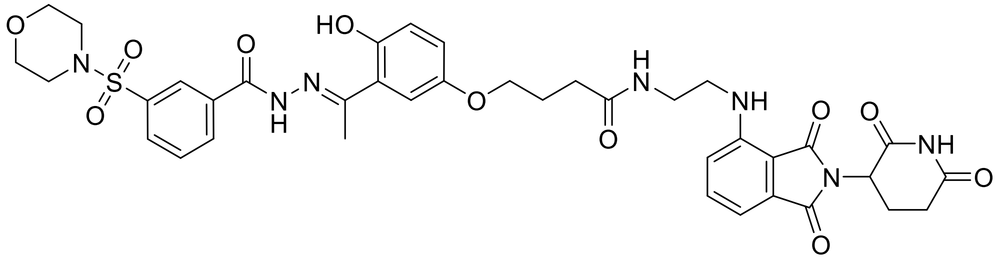

***N*-(2-((2-(2,6-dioxopiperidin-3-yl)-1,3-dioxoisoindolin-4-yl)amino)ethyl)-4-(4-hydroxy-3-(1-(2-(3-(morpholinosulfonyl)benzoyl)hydrazineylidene)ethyl)phenoxy)butanamide, 32:** The amide

coupling portion of general procedure **D** was followed using **22** (39.0 mg, 77.1 µmol), DIPEA (67.0 µL, 384 µmol), HATU (59.0 mg, 155 µmol) and **CRBN-NH$_2$** (32.7 mg, 92.6 µmol) were reacted in DMF (1.1 mL). After workup, the oil was purified on silica gel by column chromatography (0-10 % MeOH in EtOAc) followed by RP HPLC (45 – 80 % MeCN in H_2O, t_R = 13.3 mins). After lyophilising, **32** was yielded as a green solid (7.0 mg, 11 %).

^{1}H NMR (400 MHz, Methanol-*d*$_4$) δ ppm 8.20 - 8.36 (m, 2 H), 8.02 (br d, J=7.6 Hz, 1 H), 7.82 (t, J=7.6 Hz, 1 H), 7.52 (dd, J=8.5, 7.2 Hz, 1 H), 6.78 - 7.22 (m, 5 H), 6.57 (br t, J=5.2 Hz, 1 H), 5.02 (dd, J=12.6, 5.6 Hz, 1 H), 3.97 (br t, J=6.1 Hz, 2 H), 3.70 - 3.77 (m, 4 H), 3.40 - 3.52 (m, 4 H), 3.02 - 3.08 (m, 4 H), 2.56 - 2.91 (m, 4 H), 2.36 - 2.49 (m, 4 H), 2.02 - 2.13 (m, 3 H). 3 exchangeable protons not observed.

HRMS (ES+) $C_{38}H_{42}N_7O_{11}S$ requires 804.2663 found 804.2655, $C_{38}H_{41}N_7O_{11}SNa$ requires 826.2482 found 826.2462.

HPLC t_R = 28.4 mins (5 – 100 % MeCN in H_2O, 0.1 % TFA, 30 min gradient), 95 % purity.

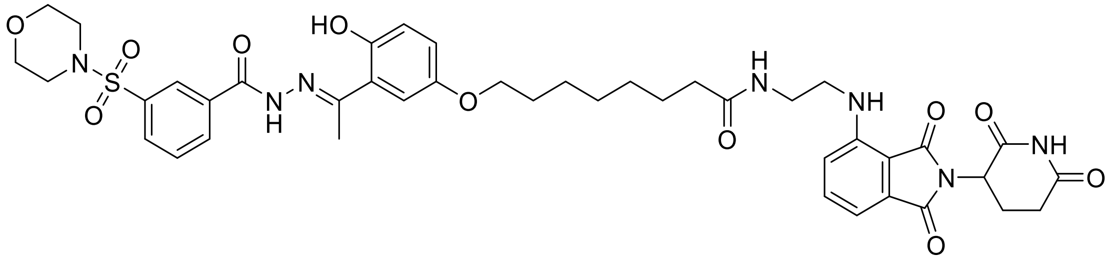

***N*-(2-((2-(2,6-dioxopiperidin-3-yl)-1,3-dioxoisoindolin-4-yl)amino)ethyl)-8-(4-hydroxy-3-(1-(2-(3-(morpholinosulfonyl)benzoyl)hydrazineylidene)ethyl)phenoxy)octanamide, 33:** The amide coupling portion of general method **D** was followed using **23** (37.4 mg, 66.5 µmol), DIPEA (58.0 µL, 333 µmol), HATU (45.6 mg, 120 µmol) and **CRBN-NH$_2$** (31.0 mg, 87.9 µmol) were reacted in DMF (0.9 mL). After workup, the oil was purified on silica gel by column chromatography (0-6 % MeOH in EtOAc) followed by RP HPLC (45 – 80 % MeCN in H_2O, t_R = 19.2 mins). After lyophilising, **33** was yielded as a green solid (3.4 mg, 11 %).

^{1}H NMR (400 MHz, Methanol-*d*$_4$) δ ppm 8.32 (s, 1 H), 8.26 (br d, J=7.9 Hz, 1 H), 8.02 (br d, J=7.9 Hz, 1 H), 7.82 (t, J=7.9 Hz, 1 H), 7.55 (dd, J=8.5, 7.1 Hz, 1 H), 7.03 - 7.17 (m, 3 H), 6.95 (dd, J=8.9, 3.1 Hz, 1 H), 6.88 (d, J=8.9 Hz, 1 H), 5.05 (dd, J=12.4, 5.5 Hz, 1 H), 3.95 (t, J=6.3 Hz, 2 H), 3.71 -

3.78 (m, 4 H), 3.40 - 3.52 (m, 4 H), 3.00 - 3.10 (m, 4 H), 2.62 - 2.91 (m, 3 H), 2.50 (s, 3 H), 2.16 - 2.24 (m, 2 H), 2.05 - 2.15 (m, 1 H), 1.68 - 1.79 (m, 2 H), 1.62 (quin, J=7.2 Hz, 2 H), 1.30 - 1.50 (m, 6 H). 5 exchangeable protons not observed.

HRMS (ES+) $C_{42}H_{50}N_7O_{11}S$ requires 860.3289 found 860.3278, $C_{42}H_{49}N_7O_{11}SNa$ requires 882.3108 found 882.3098.

HPLC t_R = 31.5 mins (5 – 100 % MeCN in H_2O, 0.1 % TFA, 30 min gradient), 97 % purity.

1.3. Synthesis of TCP related compounds

1.3.1. Synthesis of TCP-amide analogues **38-41**

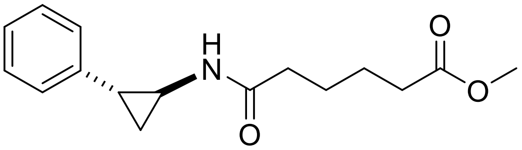

Trans-methyl 6-oxo-6-((2-phenylcyclopropyl)amino)hexanoate, 34: To a solution of mono-methyl adipate (0.113 g, 0.707 mmol, 1.2 eq.) in dry DMF (4 mL) at 0 °C under nitrogen, DIPEA (0.308 mL, 1.768 mmol, 3.0 eq.) and HATU (0.336 g, 0.884 mmol, 1.5 eq.) were added. The resulting solution stirred for 15 mins, after which a solution of tranylcypromine hydrochloride (0.100 g, 0.589 mmol, 1.0 eq.) in dry DMF (4 mL) was added slowly. The reaction was allowed to warm to RT and was stirred for 18 h and monitored by TLC to completion. The reaction mixture was diluted with EtOAc (20 mL) and the organic phase was washed with saturated sodium hydrogen carbonate solution (2 x 10 mL) and brine (2 x 10 mL). The organic phase was dried over anhydrous sodium sulfate, filtered and concentrated *in vacuo* to yield the crude product as a brown oil. This was purified by silica column chromatography (50 % EtOAc in hexane) to yield **34** as an off-white solid (0.101 g, 62 %).

^{1}H NMR (400 MHz, Methanol-d$_4$) δ ppm 7.21–7.28 (m, 2 H), 7.09–7.19 (m, 3 H), 3.64–3.68 (m, 3 H), 2.85 (ddd, J=7.7, 4.4, 3.7 Hz, 1 H), 2.32–2.39 (m, 2 H), 2.16–2.23 (m, 2 H), 2.01 (ddd, J=9.6, 6.3, 3.4 Hz, 1 H), 1.59–1.67 (m, 4 H), 1.12–1.23 (m, 2 H).

^{13}C NMR (101 MHz, Methanol-d$_4$) δ ppm 177.2, 175.8, 142.4, 129.5, 127.4, 127.1, 52.2, 36.6, 34.6, 33.4, 26.5, 25.7, 25.3, 16.2.

HRMS (ESI): [M+H]+ m/z calculated for $C_{16}H_{22}NO_3$: 276.1600, found 276.1609; [M+Na]+ m/z calculated for $C_{16}H_{21}NO_3Na$: 298.1419, found 298.1433.

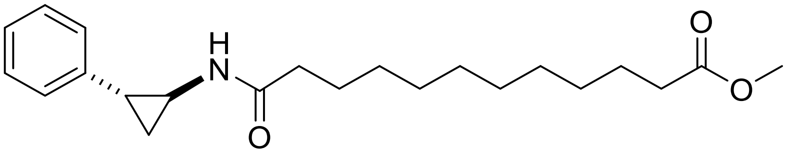

Trans-methyl 12-oxo-12-((2-phenylcyclopropyl)amino)dodecanoate, 35: To a solution of 12-(methyloxy)-12-oxododecanoic acid (0.1299 g, 0.532 mmol, 1.2 eq.) in dry DMF (3 mL) at 0 °C under nitrogen, DIPEA (0.231 mL, 1.323 mmol, 3.0 eq.) and HATU (0.2524 g, 0.664 mmol, 1.5 eq.) were added. The resulting solution stirred for 15 mins, after which a solution of tranylcypromine hydrochloride (0.0752 g, 0.443 mmol, 1.0 eq.) in dry DMF (3 mL) was added slowly. The reaction was allowed to warm to RT and was stirred for 18 h and monitored by TLC to completion. The reaction mixture concentrated *in vacuo*, redissolved in EtOAc (20 mL) and the organic phase was washed with saturated sodium hydrogen carbonate solution (2 x 10 mL) and brine (2 x 10 mL). The organic phase was dried over anhydrous sodium sulfate, filtered and concentrated *in vacuo* to yield the crude product as an off-white oil (0.286 g). This was purified by silica column chromatography (25 % EtOAc in hexane) to yield **35** as an off-white solid (0.118 g, 74 %).

^{1}H NMR (400 MHz, $CDCl_3$) δ ppm 7.12–7.34 (m, 5 H), 5.72 (br s, 1 H), 3.66 (s, 3 H), 2.85–2.92 (m, 1 H), 2.30 (t, J=7.5 Hz, 2 H), 2.15 (t, J=7.5 Hz, 2 H), 2.03 (ddd, J=9.6, 6.2, 3.4 Hz, 1 H), 1.55–1.69 (m, 4 H), 1.19–1.39 (m, 14 H), 1.14 (ddd, J=9.8, 5.6, 4.5 Hz, 1 H).

^{13}C NMR (101 MHz, Methanol-d_4) δ ppm 174.4, 174.2, 140.5, 128.4, 126.6, 126.1, 51.5, 36.6, 34.1, 32.0, 29.34, 29.28, 29.26, 29.2, 29.1, 25.6, 24.9, 24.8, 16.3.

HRMS (ESI): [M+H]+ m/z calculated for $C_{22}H_{34}NO_3$: 360.2539, found 360.2539; [M+Na]+ m/z calculated for $C_{22}H_{33}NO_3Na$: 382.2358, found 382.2355.

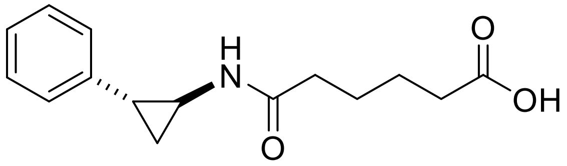

Trans-6-oxo-6-((2-phenylcyclopropyl)amino)hexanoic acid, 36: 34 (0.101 g, 0.376 mmol, 1.0 eq.) was dissolved in THF (3 mL) and LiOH (1 M aq., 1.5 mL, xs) was added. The solution was stirred overnight at RT and the reaction monitored by TLC. A further 0.5 mL of 1 M LiOH solution was added, then the reaction was stirred for a further 2 h. The solution was concentrated *in vacuo*, then deionised water (10 mL) was added. The solution was acidified with HCl solution (1 M aq.) to ~pH 2, then ethyl acetate (10 mL) was added. The aqueous phase was extracted with ethyl acetate (3 x 10

mL) and the organic phases were combined, dried over anhydrous sodium sulfate, filtered and concentrated *in vacuo* to yield **36** as a colourless oil (0.080 g, 83 %).

^{1}H NMR (400 MHz, Methanol-d$_4$) δ ppm 7.19 - 7.29 (m, 2 H), 7.08 - 7.18 (m, 3 H), 2.84 (ddd, J=7.7, 4.4, 3.6 Hz, 1 H), 2.31 (t, J=7.0 Hz, 2 H), 2.20 (t, J=7.2 Hz, 2 H), 2.01 (ddd, J=9.6, 6.3, 3.4 Hz, 1 H), 1.57 - 1.70 (m, 4 H), 1.12 - 1.22 (m, 2 H).

^{13}C NMR (101 MHz, Methanol-d$_4$) δ ppm 177.4, 177.3, 142.5, 129.5, 127.4, 127.1, 36.7, 34.7, 33.4, 26.5, 25.7, 25.3, 16.2.

HRMS (ESI): [M+H]+ m/z calculated for $C_{15}H_{20}NO_3$: 262.1443, found 262.1435; [M+Na]+ m/z calculated for $C_{15}H_{19}NO_3Na$: 284.1263, found 284.1252.

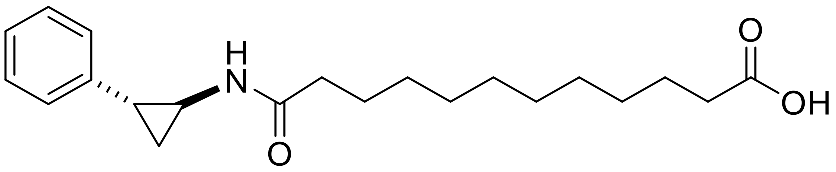

Trans-12-oxo-12-((2-phenylcyclopropyl)amino)dodecanoic acid, 37: 35 (0.118 g, 0.328 mmol, 1.0 eq.) was dissolved in THF (4 mL) and LiOH (1 M aq., 2 mL, xs) was added. The solution was stirred overnight at RT and the reaction monitored by TLC. 0.5 mL of further LiOH solution was added, then stirred for a further 3 h. The solution was concentrated *in vacuo*, then deionised water (10 mL) was added. The solution was acidified with HCl solution (1 M aq.) to ~pH 2, then ethyl acetate (10 mL) was added. The aqueous phase was extracted with ethyl acetate (3 x 10 mL) and the organic phases were combined, dried over anhydrous sodium sulfate, filtered and concentrated *in vacuo* to yield **37** as a white solid (0.079 g, 70 %).

^{1}H NMR (400 MHz, Methanol-d$_4$) δ ppm 7.20 - 7.28 (m, 2 H), 7.08 - 7.19 (m, 3 H), 2.84 (ddd, J=7.7, 4.4, 3.5 Hz, 1 H), 2.27 (t, J=7.4 Hz, 2 H), 2.17 (t, J=7.5 Hz, 2 H), 2.00 (ddd, J=9.5, 6.3, 3.5 Hz, 1 H), 1.53 - 1.65 (m, 4 H), 1.32 (s, 12 H), 1.10 - 1.23 (m, 2 H).

^{13}C NMR (101 MHz, Methanol-d_4) δ ppm 177.9, 177.8, 142.5, 129.5, 127.4, 127.1, 37.1, 35.2, 33.4, 30.67, 30.53, 30.52, 30.41, 30.38, 27.1, 26.3, 25.3, 16.3.

HRMS (ESI): [M+H]+ m/z calculated for $C_{21}H_{32}NO_3$: 346.2382, found 346.2374; [M+Na]+ m/z calculated for $C_{21}H_{32}NO_3Na$: 368.2202, found 368.2193.

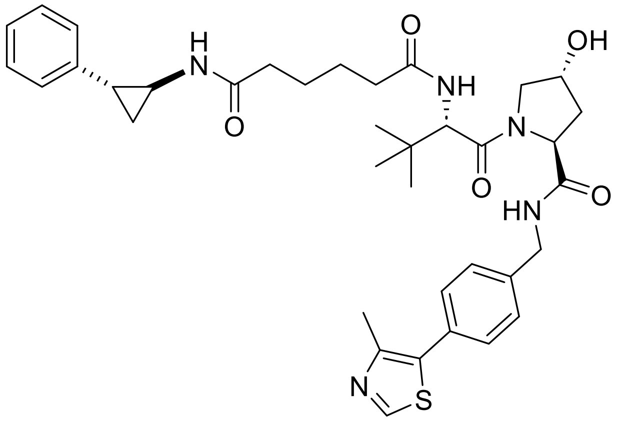

N^1-((S)-1-((2S,4R)-4-hydroxy-2-((4-(4-methylthiazol-5-yl)benzyl)carbamoyl)pyrrolidin-1-yl)-3,3-dimethyl-1-oxobutan-2-yl)-N^6-(2-phenylcyclopropyl)adipamide, 38: To a solution of **36** (0.015 g, 0.059 mmol, 1.0 eq.) in dry DMF (1 mL) at 0 °C under nitrogen, DIPEA (0.021 mL, 0.161 mmol, 3.0 eq) and HATU (0.021 g, 0.080 mmol, 1.5 eq) were added. The resulting solution stirred for 15 mins, after which a solution of **VHL-NH₂** (0.025 g, 0.054 mmol, 1.0 eq) in dry DMF (1 mL) was added slowly. The reaction was allowed to warm to RT and was stirred for 18 h and monitored by TLC to completion. The reaction mixture was diluted with EtOAc (10 mL) and the organic phase was washed with saturated sodium hydrogen carbonate solution (2 x 10 mL) and brine (2 x 10 mL). The organic phase was dried over anhydrous sodium sulfate, filtered and concentrated *in vacuo* to yield the crude product as a dark brown tar (0.030 g). This was purified by silica column chromatography (10 % MeOH in DCM) to yield **38** as a yellow solid (0.0117 g, 32 %).

^1H NMR (400 MHz, Methanol-d_4) δ ppm 8.84 - 8.90 (m, 1 H), 7.37 - 7.52 (m, 4 H), 7.18 (s, 2 H), 7.04 - 7.16 (m, 3 H). 4.47 - 4.66 (m, 4 H), 4.31 - 4.40 (m, 1 H), 3.85 - 3.93 (s, 1 H). 3.77 - 3.84 (m, 1 H), 2.80 - 2.88 (m, 1 H), 2.45 - 2.49 (m, 3 H), 1.96 - 2.37 (m, 7 H). 1.57 - 1.69 (m, 4 H), 1.11 - 1.22 (m, 2 H), 1.00 - 1.07 (m, 9 H).

^{13}C NMR (101 MHz, Methanol-d4) δ ppm 177.2, 175.8, 174.6, 172.5, 153.0, 149.2, 142.4, 140.4, 133.6, 131.7, 130.5, 129.5, 129.1, 127.4, 127.1, 71.2, 61.0, 59.2, 58.2, 43.9, 39.1, 36.7, 36.5, 33.4, 27.2, 26.7, 26.7, 25.4, 25.3, 16.3, 16.0.

HRMS (ESI): $[M+H]^+$ m/z calculated for $C_{37}H_{48}N_5O_5S$: 674.3376, found 674.3381; $[M+Na]^+$ m/z calculated for $C_{37}H_{47}N_5O_5SNa$: 696.3196, found 696.3209.

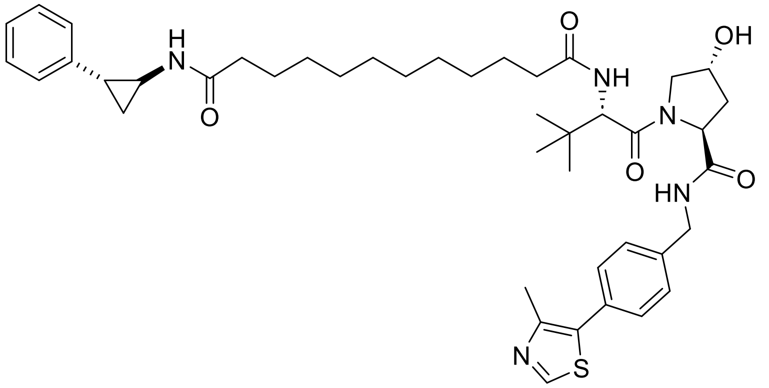

N^1-((S)-1-((2S,4R)-4-hydroxy-2-((4-(4-methylthiazol-5-yl)benzyl)carbamoyl)pyrrolidin-1-yl)-3,3-dimethyl-1-oxobutan-2-yl)-N^{12}-(2-phenylcyclopropyl)dodecanediamide, 39: To a solution of **37** (0.041 g, 0.119 mmol, 1.0 eq.) in dry DMF (2 mL) at 0 °C under nitrogen, DIPEA (0.052 mL, 0.298 mmol, 3.0 eq) and HATU (0.057 g, 0.149 mmol, 1.2 eq) were added. The resulting solution stirred for 15 mins, after which a solution of **VHL-NH_2** (0.050 g, 0.099 mmol, 1.0 eq) in dry DMF (2 mL) was added slowly. The reaction was allowed to warm to RT and was stirred for 18 h and monitored by TLC to completion. The reaction mixture was diluted with EtOAc (10 mL) and the organic phase was washed with saturated sodium hydrogen carbonate solution (2 x 10 mL) and brine (2 x 10 mL). The organic phase was dried over anhydrous sodium sulfate, filtered and concentrated *in vacuo* to yield the crude product as a brown oil (0.072 g). This was purified by silica column chromatography (7.5% MeOH in DCM) to yield **39** as a colourless tar (0.055 g, 73%).

^1H NMR (400 MHz, Methanol-d4) δ ppm 8.86 - 8.88 (m, 1 H), 7.39 - 7.49 (m, 4 H), 7.20 - 7.28 (m, 2 H), 7.09 - 7.17 (m, 3 H), 4.47 - 4.67 (m, 4 H), 4.35 (d, J=15.5 Hz, 1 H), 3.87 - 3.94 (m, 1 H), 3.77 - 3.83 (m, 1 H), 2.84 (ddd, J=7.7, 4.5, 3.5 Hz, 1 H), 2.46 - 2.49 (m, 3 H), 2.03 - 2.35 (m, 6 H), 2.00 (ddd, J=9.5, 6.2, 3.5 Hz, 1 H), 1.52 - 1.69 (m, 4 H), 1.27 - 1.40 (m, 12 H), 1.10 - 1.22 (m, 2 H), 0.99 - 1.08 (m, 9 H).

^{13}C NMR (101 MHz, Methanol-d4) δ ppm 177.7, 176.2, 174.6, 172.5, 153.0, 149.2, 142.4, 140.4, 133.6, 131.7, 130.5, 129.5, 129.1, 127.4, 127.1, 71.2, 61.0, 59.1, 58.2, 43.8, 39.1, 37.1, 36.8, 36.7, 33.4, 30.69, 30.67, 30.56, 30.53, 30.45, 30.42, 27.19, 27.16, 27.09, 25.3, 16.3, 16.0.

HRMS (ESI): [M+H]+ m/z calculated for $C_{43}H_{60}N_5O_5S$: 758.4315, found 758.4307.

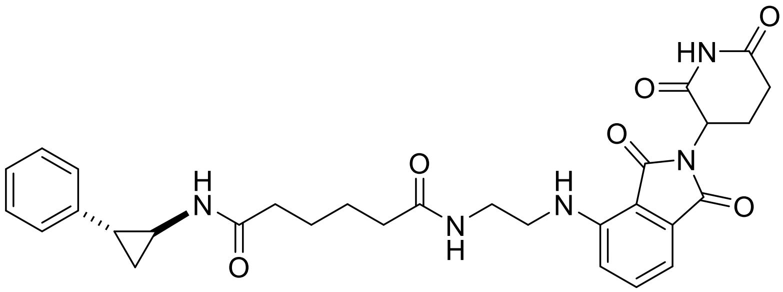

N^1-(2-((2-(2,6-dioxopiperidin-3-yl)-1,3-dioxoisoindolin-4-yl)amino)ethyl)-N^6-(2-phenylcyclopropyl)adipamide, 40: To a solution of **36** (0.0224 g, 0.085 mmol, 1.2 eq.) in dry DMF (1 mL) at 0 °C under nitrogen, DIPEA (0.037 mL, 0.213 mmol, 3.0 eq) and HATU (0.0404 g, 0.106 mmol, 1.2 eq) were added. The resulting solution stirred for 15 mins, after which a solution of **CRBN-NH_2** (0.025 g, 0.071 mmol, 1.0 eq) in dry DMF (1 mL) was added slowly. The reaction was allowed to warm to RT and was stirred for 18 h and monitored by TLC to completion. The reaction mixture was diluted with EtOAc (10 mL) and the organic phase was washed with saturated sodium hydrogen carbonate solution (2 x 10 mL) and brine (2 x 10 mL). The organic phase was dried over anhydrous sodium sulfate, filtered and concentrated *in vacuo* to yield the crude product as a yellow tar (0.036 g). This was purified by silica column chromatography (5 % MeOH in DCM) to yield **40** as a yellow solid (0.018 g, 50.6 %).

^{1}H NMR (400 MHz, Methanol-d4) δ ppm 7.49 - 7.57 (m, 1 H), 7.19 - 7.28 (m, 2 H), 7.07 - 7.17 (m, 4 H), 7.04 (d, J=6.8 Hz, 1 H), 5.04 (dd, J=12.5, 5.4 Hz, 1 H), 3.38 - 3.50 (m, 4 H), 2.62 - 2.89 (m, 4 H), 2.03 - 2.24 (m, 5 H), 1.98 (ddd, J=9.5, 6.2, 3.5 Hz, 1 H), 1.52 - 1.66 (m, 4 H), 1.07 - 1.21 (m, 2 H).

^{13}C NMR (101 MHz, Methanol-d4) δ ppm 177.2, 176.6, 174.8, 171.8, 170.7, 169.4, 148.2, 142.5, 137.4, 134.1, 129.5, 127.3, 127.1, 118.2, 112.3, 111.6, 50.3, 42.9, 39.9, 36.9, 36.7, 33.4, 32.3, 26.6, 26.6, 25.3, 23.9, 16.3.

HRMS (ESI): [M+H]+ m/z calculated for $C_{30}H_{34}N_5O_6$: 560.2509, found 560.2507; [M+Na]+ m/z calculated for $C_{30}H_{33}N_5O_6Na$: 582.2329, found 582.2328.

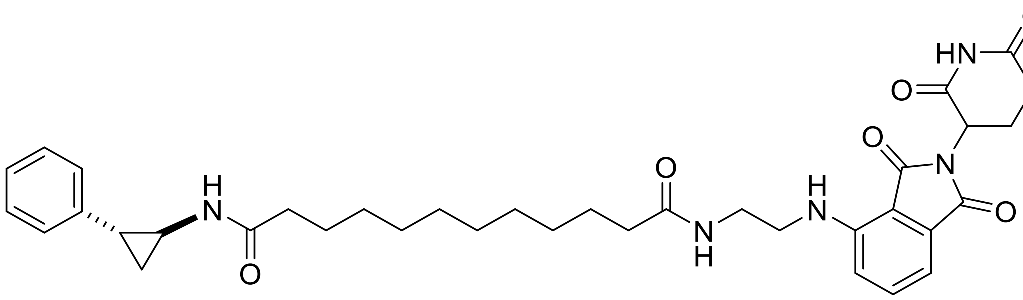

N^1-(2-((2-(2,6-dioxopiperidin-3-yl)-1,3-dioxoisoindolin-4-yl)amino)ethyl)-N^{12}-(2-phenylcyclopropyl)dodecanediamide, 41: To a solution of **37** (0.0357 g, 0.103 mmol, 1.2 eq.) in dry DMF (1 mL) at 0 °C under nitrogen, DIPEA (0.044 mL, 0.255 mmol, 3.0 eq) and HATU (0.0486 g, 0.128 mmol, 1.5 eq) were added. The resulting solution stirred for 15 mins, after which a solution of **CRBN-NH2** (0.0300 g, 0.085 mmol, 1.0 eq) in dry DMF (1 mL) was added slowly. The reaction was allowed to warm to RT and was stirred for 18 h and monitored by TLC to completion. The reaction mixture was diluted with EtOAc (10 mL) and the organic phase was washed with saturated sodium hydrogen carbonate solution (2 x 10 mL) and brine (2 x 10 mL). The organic phase was dried over anhydrous sodium sulfate, filtered and concentrated *in vacuo* to yield the crude product as a yellow oil (0.053 g). This was purified by silica column chromatography twice (first manually in 5 % MeOH in DCM, then again in 1-9 % MeOH in DCM) to yield **41** as a yellow solid (0.032 g, 59 %).

^{1}H NMR (400 MHz, Methanol-d4) δ ppm 7.53 (dd, J=8.5, 7.2 Hz, 1 H), 7.20 - 7.26 (m, 2 H), 7.08 - 7.15 (m, 4 H), 7.04 (d, J=7.1 Hz, 1 H), 5.04 (dd, J=12.5, 5.5 Hz, 1 H), 3.39 - 3.48 (m, 4 H), 2.63 - 2.91 (m, 4 H), 2.04 - 2.20 (m, 5 H), 1.99 (ddd, J=9.6, 6.3, 3.4 Hz, 1 H), 1.52 - 1.63 (m, 5 H), 1.10 - 1.34 (m, 16 H).

^{13}C NMR (101 MHz, Methanol-d_4) δ ppm 177.7, 177.0, 174.8, 171.7, 170.7, 169.4, 148.3, 142.4, 137.4, 134.1, 129.5, 127.4, 127.1, 118.2, 112.2, 111.6, 50.3, 42.9, 39.9, 37.3, 37.1, 33.4, 32.4, 30.64, 30.63, 30.53, 30.50, 30.39, 30.37, 27.09, 27.06, 25.3, 24.0, 16.3.

HRMS (ESI): [M+H]+ m/z calculated for $C_{36}H_{46}N_5O_6$: 644.3448, found 644.3451; [M+Na]+ m/z calculated for $C_{36}H_{45}N_5O_6Na$: 666.3268, found 666.3262.

1.3.2 Synthesis of starting material **42**

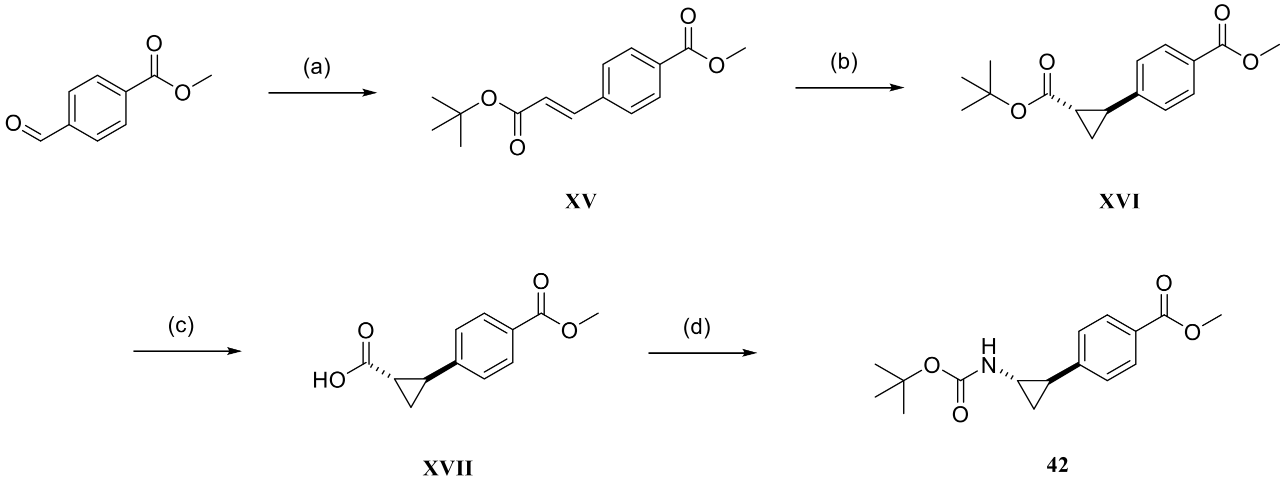

Reagents and conditions: (a) Tert-butyl diethylphosphonoacetate, KO^{t}Bu, THF, 0 °C, 24 h, 80%; (b) Trimethylsulfoxonium iodide, KO^{t}Bu, DMSO, rt, 24 h, 52%; (c) TFA, DCM, rt, 18 h, 78%; (d) DPPA, Et_3N, tBuOH, reflux, 48 h, 26%.

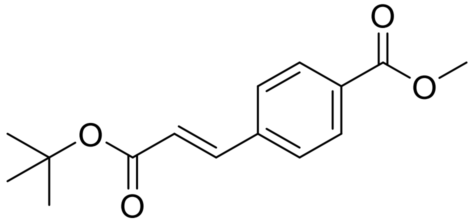

Methyl (*E*)-4-(3-(*tert*-butoxy)-3-oxoprop-1-en-1-yl)benzoate, XV: To a solution of potassium tert-butoxide (0.753 g, 6.701 mmol, 1.1 eq.) in dry THF (30 mL) at 0 °C under nitrogen, Tert-butyl diethylphosphonoacetate (1.573 mL, 6.701 mmol, 1.1 eq.) was added dropwise over 15 mins. The solution was stirred at the same temperature for 1 h, following which methyl-4-formylbenzoate (1.000 g, 6.092 mmol, 1.0 eq.) dissolved in dry THF (10 mL) was added over 20 mins to the solution with vigorous stirring at the same temperature. After this, the solution was allowed to warm to RT and was stirred overnight. The reaction was monitored by TLC to completion. It was poured into iced water (50 mL) and extracted with ethyl acetate (5 x 25 mL). The combined organic phases were washed with saturated sodium bicarbonate soluton (25 mL), water (25 mL) and brine (25 mL), then dried over sodium sulfate and concentrated *in vacuo* to yield the crude colourless oil or white solid (1.932 g). This was purified by silica column chromatography (12.5 % ethyl acetate in hexane) to yield **XV** as a white solid (1.283 g, 80 %).

^{1}H NMR (400 MHz, $CDCl_3$) δ ppm 8.03 (d, J=8.3 Hz, 2 H), 7.54 - 7.63 (m, 3 H), 6.45 (d, J=16.0 Hz, 1 H), 3.93 (s, 3 H), 1.54 (s, 9 H).

^{13}C NMR (101 MHz, $CDCl_3$) δ ppm 166.5, 165.8, 142.1, 138.9, 131.1, 130.0, 127.8, 122.6, 80.9, 52.2, 28.1.

HRMS (ESI): [M-(C_4H_8)]+ m/z calculated for $C_{11}H_{11}O_4$: 207.0657, found 207.0660.

Data was consistent with previously reported data.[9]

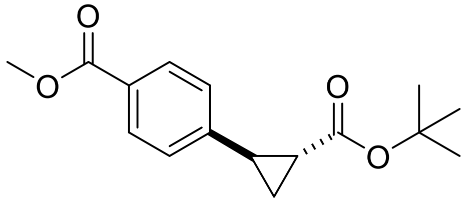

Methyl 4-((trans)-2-(*tert*-butoxycarbonyl)cyclopropyl)benzoate, XVI: Potassium tert-butoxide (0.631 g, 5.625 mmol, 1.15 eq.) and trimethylsulfoxonium iodide (1.184 g, 5.380 mmol, 1.1 eq.) was dissolved in dry DMSO (25 mL) and stirred under nitrogen at RT for 30 mins. **XV** (1.283 g, 4.891 mmol, 1.0 eq.) dissolved in further DMSO (20 mL) was then added dropwise over 30 mins, following which the reaction mixture was stirred overnight at RT under nitrogen. Water (50 mL) and ethyl acetate (50 mL) was added and the organic phase separated, following which this was extracted with ethyl acetate (5 x 25 mL). Combined organic layers were washed with saturated brine (1 x 50 mL), dried over sodium sulfate and concentrated *in vacuo* to yield the crude product as a yellow liquid (1.8 g, containing residual DMSO). This was purified by silica column chromatography (10 % ethyl acetate in hexane) to yield **XVI** as a colourless oil (0.750 g, 52 %).

^{1}H NMR (400 MHz, $CDCl_3$) δ ppm 7.94 (d, J=8.4 Hz, 2 H), 7.13 (d, J=8.2 Hz, 2 H), 3.90 (s, 3 H), 2.48 (ddd, J=9.2, 6.3, 4.2 Hz, 1 H), 1.90 (ddd, J=8.5, 5.4, 4.2 Hz, 1 H), 1.59 (ddd, J=9.2, 5.4, 4.6 Hz, 1 H), 1.48 (s, 9 H), 1.24 - 1.31 (m, 2 H).

^{13}C NMR (101 MHz, $CDCl_3$) δ ppm 171.7, 166.5, 145.8, 129.4, 127.8, 125.6, 80.5, 51.7, 27.8, 25.5, 25.2, 17.2.

HRMS (ESI): [M-(C_4H_8)]+ m/z calculated for $C_{12}H_{13}O_4$: 221.0814, found 221.0803.

Data was consistent with previously reported data. [9]

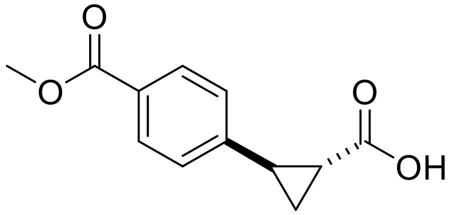

(Trans)-2-(4-(methoxycarbonyl)phenyl)cyclopropane-1-carboxylic acid, XVII: XVI (0.788 g, 2.852 mmol, 1.0 eq.) was dissolved in dry DCM (15 mL) with stirring under nitrogen. Trifluoroacetic acid (2.84 mL, 37.07 mmol, 13 eq.) was added slowly and the reaction was stirred at RT for 18 h. The reaction was monitored by TLC to completion, then it was concentrated *in vacuo*. The residue was redissolved in DCM and concentrated again (3 x 10 mL), to yield crude **XVII** as a pale yellow residue, which was used without further purification (0.549 g, 78 %).

^{1}H NMR (400 MHz, $CDCl_3$) δ ppm 11.55 (br s, 1 H), 7.96 (d, J=8.4 Hz, 2 H), 7.16 (d, J=8.4 Hz, 2 H), 3.93 (s, 3 H), 2.65 (ddd, J=9.5, 6.8, 4.2 Hz, 1 H), 1.99 (ddd, J=8.8, 5.1, 4.1 Hz, 1 H), 1.75 (dt, J=9.6, 5.0 Hz, 1 H), 1.48 (ddd, J=8.4, 6.7, 4.8 Hz, 1 H).

^{13}C NMR (101 MHz, $CDCl_3$) δ ppm 179.5, 167.3, 144.9, 129.9, 128.4, 126.2, 52.3, 27.1, 24.4, 18.1.

HRMS (ESI): [M+H]+ m/z calculated for $C_{12}H_{13}O_4$: 221.0814, found 221.0811.

Data was consistent with previously reported data.[9]

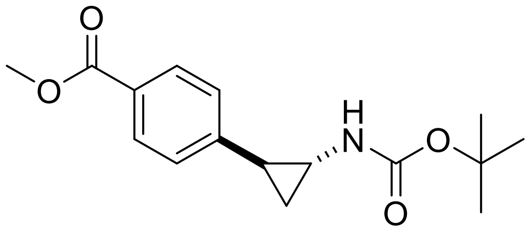

Methyl 4-((trans)-2-((*tert*-butoxycarbonyl)amino)cyclopropyl)benzoate, 42: XVII (0.439 g, 1.993 mmol, 1.0 eq.) was dissolved in tert-butanol (10 mL), to which was added triethylamine (0.42 mL, 2.990 mmol, 1.5 eq.) and DPPA (0.49 mL, 2.193 mmol, 1.1 eq.). The reaction was refluxed for 48 h and monitored by TLC to completion. The solution was allowed to cool, then ethyl acetate (10 mL) was added. The organic phase was washed with saturated sodium carbonate solution (3 x 10 mL) then the aqueous phase was extracted with further ethyl acetate (10 mL). Combined organic phases were washed with saturated sodium bicarbonate solution (10 mL), water (10 mL) and brine (10 mL), dried over magnesium sulfate and concentrated *in vacuo* to yield the crude brown oil (0.637 g). This was purified by silica common chromatography (20 % ethyl acetate in hexane) to yield **42** as an off-white solid (0.149 g, 26 %).

^{1}H NMR (400 MHz, $CDCl_3$) δ ppm 7.93 (d, J=8.4 Hz, 2 H), 7.17 (d, J=8.3 Hz, 2 H), 4.70 - 5.00 (m, 1 H), 3.90 (s, 3 H), 2.78 (br s, 1 H), 2.09 (td, J=7.8, 3.0 Hz, 1 H), 1.45 (s, 9 H), 1.24 (dd, J=7.2, 6.1 Hz, 2 H).

^{13}C NMR (101 MHz, $CDCl_3$) δ ppm 167.0, 156.2, 146.4, 129.7, 127.8, 126.2, 79.8, 52.0, 33.1, 28.4, 25.3, 17.0.

HRMS (ESI): no mass observed.

Data was consistent with previously reported data.[9]

1.3.3 Synthesis of TCP-phenyl carboxylic acid intermediates **43- 46**

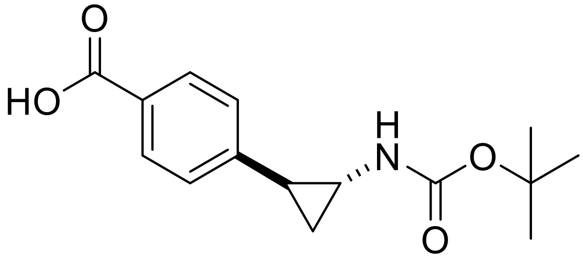

4-((Trans)-2-((tert-butoxycarbonyl)amino)cyclopropyl)benzoic acid, 43: 42 (0.1301 g, 0.447 mmol, 1.0 eq.) was dissolved in THF (4 mL) and LiOH (1 M aq., 2 mL, xs) was added. The solution was stirred overnight at RT and the reaction monitored by TLC, with a further 0.5 mL of LiOH solution added. The reaction was stirred for a further 5 h then was concentrated, then deionised water (10 mL) was added. The solution was acidified with HCl solution (1 M aq.) to ~pH 2, then ethyl acetate (10 mL) was added. The aqueous phase was extracted with ethyl acetate (3 x 10 mL) and the organic phases were combined, dried over anhydrous sodium sulphate, filtered and concentrated *in vacuo* to yield **43** as an off-white solid (110 mg, 89 %).

^{1}H NMR (400 MHz, Methanol-d_4) δ ppm 7.91 (d, J=8.3 Hz, 2 H), 7.20 (d, J=8.3 Hz, 2 H), 2.72 (br s, 1 H), 2.04 (td, J=7.7, 3.1 Hz, 1 H), 1.43 (s, 9 H), 1.19 - 1.26 (m, 2 H).

^{13}C NMR (101 MHz, $CDCl_3$) δ ppm 170.1, 159.1, 148.7, 131.0, 129.5, 127.1, 80.5, 34.7, 28.9, 26.0, 17.3.

HRMS (ESI): no mass observed.

Data was consistent with previously reported data.[10]

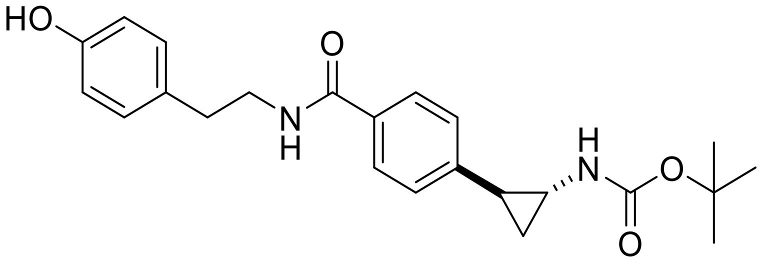

Tert-butyl ((trans)-2-(4-((4-hydroxyphenethyl)carbamoyl)phenyl)cyclopropyl)carbamate, 44: To a solution of **43** (0.0259 g, 0.0934 mmol, 1.2 eq.) in dry DMF (2 mL) at 0 °C under nitrogen,

DIPEA (0.44 mL, 0.255 mmol, 3.0 eq.) and HATU (0.0484g, 0.127 mmol, 1.5 eq.) were added. The resulting solution stirred for 15 mins, after which a solution of tyramine (0.0116 g, 0.0849 mmol, 1.0 eq.) in dry DMF (1 mL) was added slowly. The reaction was allowed to warm to RT and was stirred for 18 h and monitored by TLC to completion. The reaction mixture was diluted with EtOAc (10 mL) and the organic phase was washed with saturated sodium hydrogen carbonate solution (2 x 10 mL) and brine (2 x 10 mL). The organic phase was dried over anhydrous sodium sulphate, filtered and concentrated *in vacuo* to yield the crude product as a brown oil. This was purified by silica column chromatography (75 % EtOAc in hexane) to yield **44** as an off-white solid (0.008 g, 24 %).

^{1}H NMR (400 MHz, Methanol-d_4) δ ppm 7.67 (d, J=8.3 Hz, 2 H), 7.18 (d, J=8.3 Hz, 2 H), 7.06 (d, J=8.5 Hz, 2 H), 6.71 (d, J=8.4 Hz, 2 H), 3.52 (t, J=7.3 Hz, 2 H), 2.80 (t, J=7.4 Hz, 2 H), 2.65 - 2.73 (m, 1 H), 2.02 (td, J=7.8, 3.1 Hz, 1 H), 1.43 (s, 9 H), 1.20 (dd, J=8.0, 5.9 Hz, 2 H).

^{13}C NMR (101 MHz, Methanol-d_4) δ ppm 170.2, 157.1, 147.0, 133.3, 131.5, 131.0, 128.4, 127.2, 116.4, 43.1, 35.9, 34.4, 28.9, 25.7, 17.1.

HRMS (ESI): [M+H]+ m/z calculated for $C_{23}H_{29}N_2O_4$: 397.2127, found 397.2127.

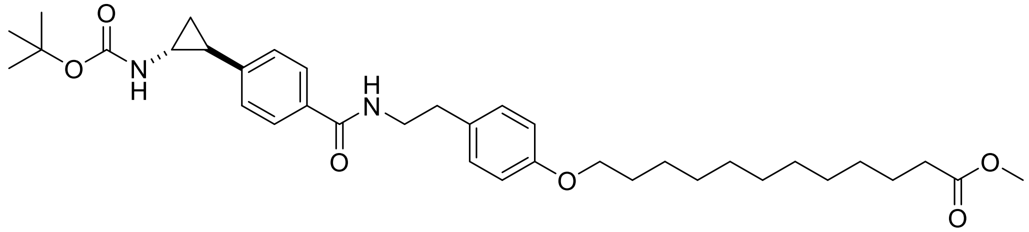

Methyl 12-(4-(2-(4-((trans)-2-((tert-butoxycarbonyl)amino)cyclopropyl)benzamido)ethyl) phenoxy)dodecanoate, 45: 44 (0.0324 g, 0.0818 mmol, 1.0 eq.), methyl 12-bromododecanoate (0.024 g, 0.0818 mmol, 1.0 eq.) and anhydrous potassium carbonate (0.0339 g, 0.246 mmol, 3.0 eq.) were dissolved in dry DMF (4 mL) and heated to 70 °C for 72 h, with further potassium carbonate (approx. 1.0 eq.) added after 48 h. On completion, the reaction was concentrated *in vacuo*. The crude material was purified by silica column chromatography (1 % methanol in DCM) to yield **45** as a white solid (24.4 mg, 49 %).

^1H NMR (400 MHz, $CDCl_3$) δ ppm 7.58 (d, J=8.3 Hz, 2 H), 7.12 (dd, J=8.5, 3.3 Hz, 4 H), 6.84 (d, J=8.6 Hz, 2 H), 6.14 (br t, J=5.7 Hz, 1 H), 3.93 (t, J=6.6 Hz, 2 H), 3.60 - 3.68 (m, 5 H), 2.84 (t, J=6.8 Hz, 2 H), 2.68 - 2.76 (m, 1 H), 2.29 (t, J=7.6 Hz, 2 H), 2.05 (td, J=7.8, 2.9 Hz, 1 H), 1.70 - 1.81 (m, 2 H), 1.61 (quin, J=7.3 Hz, 2 H), 1.44 (s, 10 H), 1.23 - 1.37 (m, 15 H), 1.19 (dd, J=7.5, 5.7 Hz, 2 H).

^{13}C NMR (101 MHz, $CDCl_3$) δ ppm 174.2, 167.1, 157.8, 144.6, 132.2, 130.6, 129.6, 126.8, 126.3, 114.6, 79.6, 68.0, 51.3, 41.2, 34.7, 34.0, 32.9, 29.5, 29.44, 29.34, 29.32, 29.24, 29.16, 29.1, 28.3, 26.0, 24.9, 16.6.

HRMS (ESI): [M+Na]+ m/z calculated for $C_{39}H_{58}N_2O_6Na$: 609.3904, found 609.3898; [M+H]+ m/z calculated for $C_{39}H_{59}N_2O_6$: 631.3723, found 631.3715.

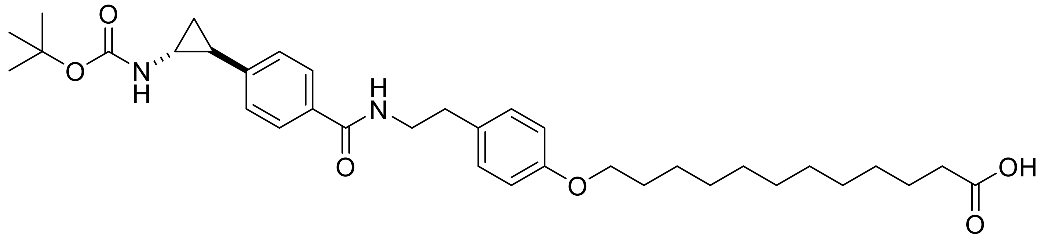

12-(4-(2-(4-((Trans)-2-((tert-butoxycarbonyl)amino)cyclopropyl)benzamido)ethyl)phenoxy) dodecanoic acid, 46: 45 (0.0364 g, 0.060 mmol, 1.0 eq.) was dissolved in THF (4 mL) and LiOH (1 M aq., 3 mL, xs) was added. The solution was stirred overnight at RT and the reaction monitored by TLC. The solution was concentrated *in vacuo*, then deionised water (10 mL) was added. The solution was acidified with HCl solution (1 M aq.) to ~pH 2, then ethyl acetate (10 mL) was added. The aqueous phase was extracted with ethyl acetate (3 x 10 mL) and the organic phases were combined, dried over anhydrous sodium sulphate, filtered and concentrated *in vacuo* to yield **46** as a colourless oil (0.0237 g, 67 %).

^1H NMR (400 MHz, $CDCl_3$) δ ppm 7.59 (d, J=8.3 Hz, 2 H), 7.13 (d, J=8.5 Hz, 4 H), 6.86 (d, J=8.5 Hz, 2 H), 6.14 (br t, J=5.6 Hz, 1 H), 4.95 (br s, 1 H), 3.94 (t, J=6.6 Hz, 2 H), 3.66 (q, J=6.6 Hz, 2 H), 2.86 (t, J=6.8 Hz, 2 H), 2.73 (br s, 1 H), 2.34 (t, J=7.5 Hz, 2 H), 2.01 - 2.10 (m, 1 H), 1.78 (quin, J=6.6 Hz, 2 H), 1.64 (quin, J=7.3 Hz, 2 H), 1.45 (s, 11 H), 1.16 - 1.39 (m, 19 H).

^{13}C NMR (101 MHz, $CDCl_3$) δ ppm 178.6, 167.3, 157.9, 144.7, 132.2, 130.6, 129.7, 126.9, 126.4, 114.7, 78.9, 68.0, 41.2, 34.7, 33.9, 29.7, 29.4, 29.34, 29.28, 29.2, 29.0, 28.4, 26.0, 25.0, 24.7, 16.7.

HRMS (ESI): [M+Na]+ m/z calculated for $C_{36}H_{52}N_2O_6Na$: 617.3567, found 617.3570; [M+H]+ m/z calculated for $C_{36}H_{53}N_2O_6$: 595.3747, found 595.3754.

1.3.4 Synthesis of VHL-based linker intermediate **50**

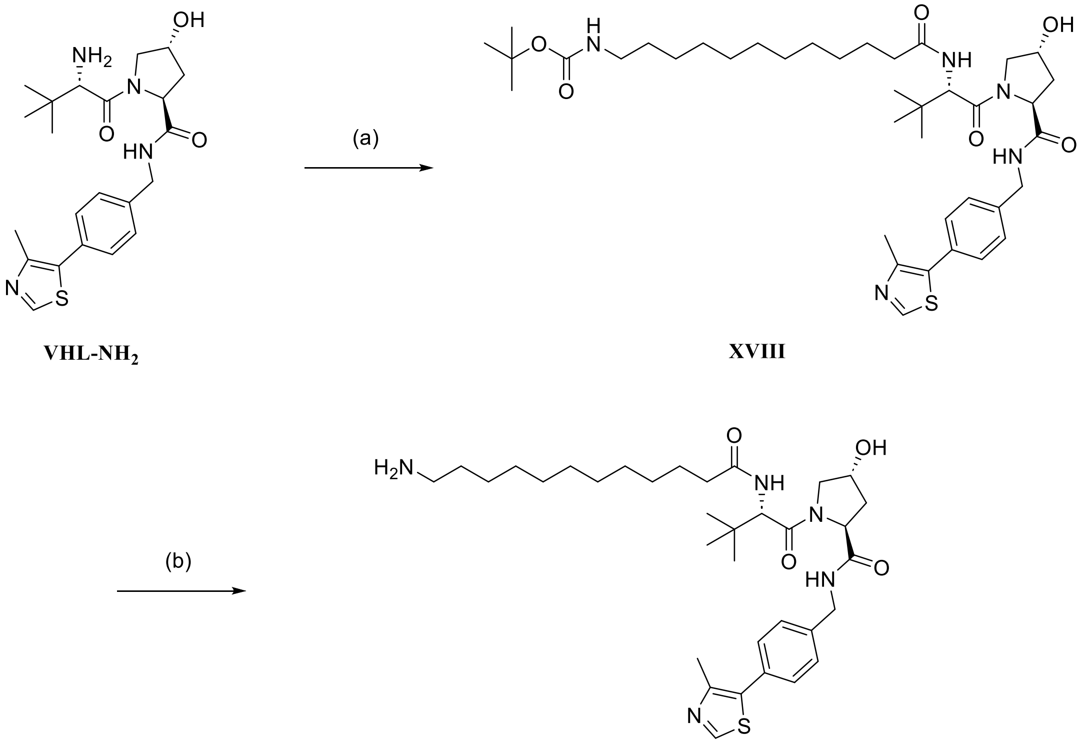

VHL-NH$_2$ **XVIII** **50**

Reagents and conditions: (a) 12-((tert-butoxycarbonyl)amino)dodecanoic acid, HATU, DIPEA, DMF, 0 °C, 4 h, 66%; (b) TFA, DCM, rt, 6 h, 64%.

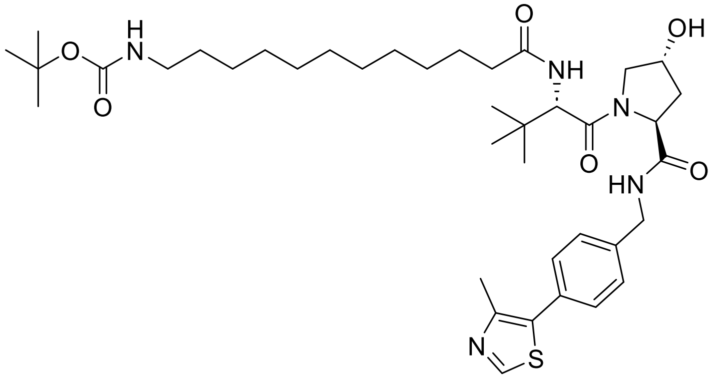

Tert-butyl (12-(((S)-1-((2S,4R)-4-hydroxy-2-((4-(4-methylthiazol-5-yl)benzyl)carbamoyl) pyrrolidin-1-yl)-3,3-dimethyl-1-oxobutan-2-yl)amino)-12-oxododecyl)carbamate, XVIII: To a solution of 12-((tert-butoxycarbonyl)amino)dodecanoic acid (0.0743 g, 0.236 mmol, 1.1 eq.) in dry DMF (4 mL) at 0 °C under nitrogen, DIPEA (0.111 mL, 0.642 mmol, 3.0 eq.) and HATU (0.122 g,

0.321 mmol, 1.5 eq.) were added. The resulting solution stirred for 15 mins, after which a solution of **VHL-NH$_2$** (hydrochloride, 0.1000 g, 0.214 mmol, 1.0 eq.) in dry DMF (4 mL) was added slowly. The reaction was allowed to warm to RT and was stirred for 18 h and monitored by TLC to completion. The reaction mixture was diluted with EtOAc (15 mL) and the organic phase was washed with saturated sodium hydrogen carbonate solution (2 x 10 mL) and brine (2 x 10 mL). The organic phase was dried over anhydrous sodium sulphate, filtered and concentrated *in vacuo* to yield the crude product as a brown oil. This was purified by silica column chromatography (5 % MeOH in DCM) to yield **XVIII** as an off-white oil (103 mg, 66 %).

^{1}H NMR (400 MHz, Methanol-*d*$_4$) δ ppm 8.87 (s, 1 H), 7.43 - 7.49 (m, 2 H), 7.38 - 7.42 (m, 2 H), 4.46 - 4.67 (m, 4 H), 4.31 - 4.40 (m, 1 H), 3.87 - 3.95 (m, 1 H), 3.77 - 3.85 (m, 1 H), 3.01 (t, J=7.0 Hz, 2 H), 2.47 (s, 3 H), 2.18 - 2.34 (m, 3 H), 2.09 (ddd, J=13.3, 9.0, 4.4 Hz, 1 H), 1.51 - 1.68 (m, 2 H), 1.42 (s, 11 H), 1.25 - 1.35 (m, 16 H), 1.03 (s, 9 H).

^{13}C NMR (101 MHz, Methanol-*d*$_4$) δ ppm 176.1, 174.5, 172.4, 158.6, 152.9, 149.1, 140.4, 133.5, 131.57, 130.5, 129.1, 79.8, 71.2, 60.9, 59.0, 58.1, 43.8, 41.5, 39.0, 36.8, 36.7, 31.1, 30.8, 30.74, 30.70, 30.6, 30.4, 29.0, 28.0, 27.2, 27.1, 16.0.

HRMS (ESI): [M+Na]+ m/z calculated for $C_{39}H_{61}N_5O_6SNa$: 750.4240, found 750.4240, [M+H]+ m/z calculated for $C_{39}H_{62}N_5O_6S$: 728.4421, found 728.4421.

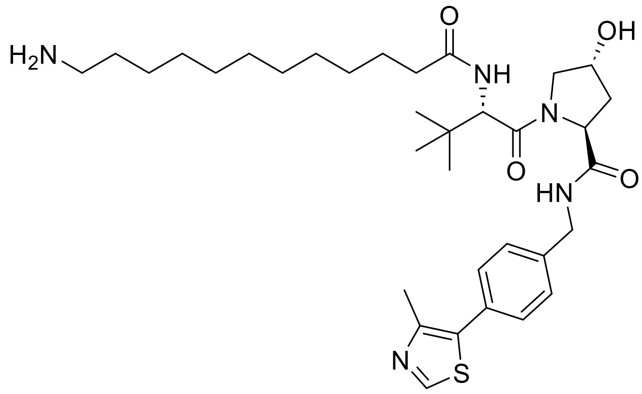

(2S,4R)-1-((S)-2-(12-aminododecanamido)-3,3-dimethylbutanoyl)-4-hydroxy-N-(4-(4-methylthiazol-5-yl)benzyl)pyrrolidine-2-carboxamide, 50: XVIII (0.105 g, 0.144 mmol, 1.0 eq.) was dissolved in DCM (8 mL) with stirring under nitrogen. Trifluoroacetic acid (0.144 mL, 1.875

mmol, 13 eq.) was added slowly and the reaction was stirred at RT for 6.5 h. The reaction was monitored by TLC to completion, then it was concentrated *in vacuo*. The residue agitated in MeOH (5 mL) with MP-carbonate resin (0.613 g, 3.02 mmol/g loading capacity) for 2 h, following which it was filtered and concentrated to yield **50** as a pale yellow oil (58 mg, 64 %).

^{1}H NMR (400 MHz, Methanol-*d*$_4$) δ ppm 8.87 (s, 1 H), 7.38 - 7.49 (m, 4 H), 4.32 - 4.67 (m, 5 H), 3.87 - 3.93 (m, 1 H), 3.77 - 3.84 (m, 1 H), 2.83 (t, J=7.4 Hz, 2 H), 2.47 (s, 3 H), 2.18 - 2.34 (m, 3 H), 2.08 (ddd, J=13.3, 9.0, 4.5 Hz, 1 H), 1.54 - 1.66 (m, 4 H), 1.24 - 1.40 (m, 15 H), 0.99 (s, 9 H).

^{13}C NMR (101 MHz, Methanol-*d*$_4$) δ ppm 176.1, 174.6, 172.5, 153.0, 149.1, 140.4, 133.5, 131.6, 130.5, 129.1, 71.2, 61.0, 59.1, 58.1, 43.8, 41.3, 39.1, 36.8, 36.7, 30.71, 30.70, 30.6, 30.5, 30.4, 30.0, 27.7, 27.2, 27.1, 16.0.

HRMS (ESI): [M+Na]+ m/z calculated for $C_{34}H_{53}N_5O_4SNa$: 650.3716, found 650.3697, [M+H]+ m/z calculated for $C_{34}H_{54}N_5O_4S$: 628.3897, found 628.3901.

1.3.5 Synthesis of **49, 52** and **54**

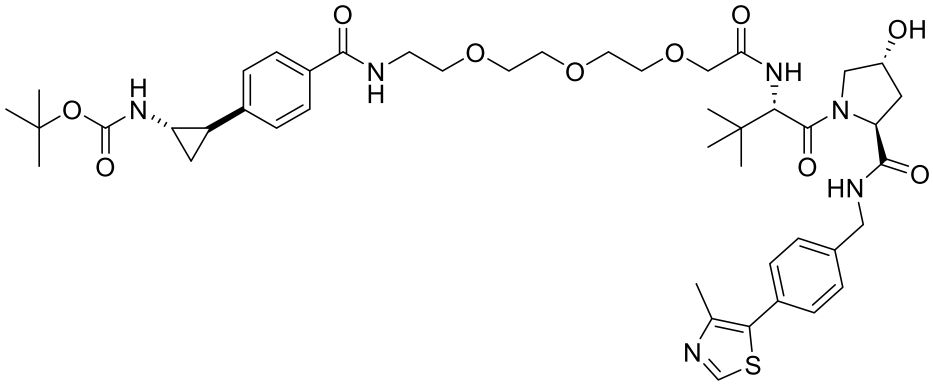

Tert-butyl ((trans)-2-(4-(((S)-13-((2S,4R)-4-hydroxy-2-((4-(4-methylthiazol-5-yl)benzyl)carbamoyl)pyrrolidine-1-carbonyl)-14,14-dimethyl-11-oxo-3,6,9-trioxa-12-azapentadecyl)carbamoyl)phenyl)cyclopropyl)carbamate, 48: To a solution of **43** (0.0090 g, 0.317 mmol, 1.1 eq.) in dry DMF (1 mL) at 0 °C under nitrogen, DIPEA (0.015 mL, 0.0866 mmol, 3.0 eq.) and HATU (0.0165 g, 0.0433 mmol, 1.5 eq.) were added. The resulting solution stirred for 15 mins, after which a solution of **47** (0.0200 g, 0.0289 mmol, 1.0 eq.) in dry DMF (1 mL) was added slowly. The reaction was allowed to warm to RT and was stirred for 18 h and monitored by TLC to completion. The reaction mixture was concentrated *in vacuo* and loaded directly onto a silica column for chromatography (3 % MeOH in DCM) to yield **48** as a yellow oil (0.020 g, 79 %).

^{1}H NMR (400 MHz, Methanol-d_4) δ ppm 8.86 (s, 1 H), 7.70 (d, J=8.3 Hz, 2 H), 7.39 - 7.46 (m, 4 H), 7.17 (d, J=8.3 Hz, 2 H), 4.70 (s, 1 H), 4.48 - 4.61 (m, 3 H), 4.34 (d, J=15.5 Hz, 1 H), 3.98 (qd, J=15.4, 2.3 Hz, 2 H), 3.83 - 3.90 (m, 1 H), 3.77 - 3.83 (m, 1 H), 3.59 - 3.70 (m, 11 H), 3.50 - 3.58 (m, 2 H), 2.69 (br s, 1 H), 2.47 (s, 3 H), 2.22 (ddt, J=13.0, 7.6, 1.5, 1.5 Hz, 1 H), 2.08 (ddd, J=13.4, 9.2, 4.3 Hz, 1 H), 2.01 (td, J=8.3, 2.9 Hz, 1 H), 1.43 (s, 9 H), 1.19 (t, J=7.5 Hz, 2 H), 1.04 (s, 9 H).

^{13}C NMR (101 MHz, Methanol-d_4) δ ppm 174.5, 172.2, 171.8, 170.2, 153.0, 149.2, 147.1, 140.4, 133.5, 133.1, 131.7, 130.5, 129.1, 128.5, 127.2, 80.4, 72.4, 71.7, 71.6, 71.5, 71.2, 70.7, 61.0, 58.3, 58.2, 43.9, 41.1, 39.1, 37.3, 34.5, 28.9, 27.1, 25.7, 17.2, 16.0.

HRMS (ESI): [M+Na]+ m/z calculated for $C_{45}H_{62}N_6O_{10}SNa$: 901.4146, found 901.4156, [M+H]+ m/z calculated for $C_{45}H_{63}N_6O_{10}S$: 879.4326, found 879.4340.

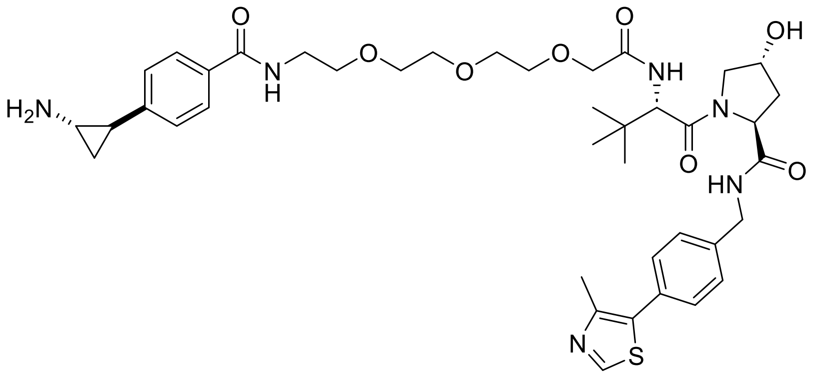

(2S,4R)-1-((15S)-1-(4-((trans)-2-aminocyclopropyl)phenyl)-15-(tert-butyl)-1,13-dioxo-5,8,11-trioxa-2,14-diazahexadecan-16-oyl)-4-hydroxy-N-(4-(4-methylthiazol-5-yl)benzyl)pyrrolidine-2-carboxamide, 49: 48 (0.020 g, 0.0228 mmol, 1.0 eq.) was dissolved in DCM (2 mL) with stirring under nitrogen. Trifluoroacetic acid (0.05 mL, 0.296+ mmol, 13 eq.) was added slowly and the reaction was stirred at RT for 3 h. The reaction was monitored by TLC to completion, then it was concentrated in vacuo. The residue was agitated in MeOH (5 mL) with MP-carbonate resin (0.2290 g, 3.02 mol/g) for 2.5 h, following which it was filtered and concentrated. This was columned on alumina (5% MeOH in DCM) to yield **49** as an off-white solid (0.011 g, 62%).

^{1}H NMR (400 MHz, Methanol-d$_4$) δ ppm 8.87 (s, 1 H), 7.69 (d, J=8.3 Hz, 2 H), 7.43 (m, 4 H), 7.03 - 7.19 (m, 2 H), 4.69 (s, 1 H), 4.48 - 4.61 (m, 3 H), 4.34 (d, J=15.5 Hz, 1 H), 3.97 - 4.03 (m, 1 H), 3.89 - 3.97 (m, 1 H), 3.83 - 3.89 (m, 1 H), 3.77 - 3.83 (m, 1 H), 3.48 - 3.71 (m, 12 H), 2.55 (dddd, J=7.51, 4.6, 3.1, 1.9 Hz, 1 H), 2.47 (s, 3 H), 2.17 - 2.26 (m, 1 H), 2.08 (ddd, J=13.2, 9.1, 4.4 Hz, 1 H), 1.96 (ddd, J=9.2, 6.0, 3.3 Hz, 1 H), 0.98 - 1.17 (m, 11 H).

^{13}C NMR (101 MHz, Methanol-d$_4$) δ ppm 174.5, 172.2, 171.8, 170.2, 153.0, 149.2, 147.8, 140.4, 133.6, 132.9, 131.7, 130.5, 129.1, 128.6, 126.8, 72.4, 71.7, 71.6, 71.5, 71.2, 70.7, 61.0, 58.2, 43.9, 41.1, 39.1, 37.3, 36.4, 27.1, 26.2, 18.6, 16.0.

HRMS (ESI): [M+Na]+ m/z calculated for $C_{40}H_{54}N_6O_8SNa$: 801.3622, found 801.3638, [M+H]+ m/z calculated for $C_{40}H_{55}N_6O_8S$: 779.3802, found 779.3822.

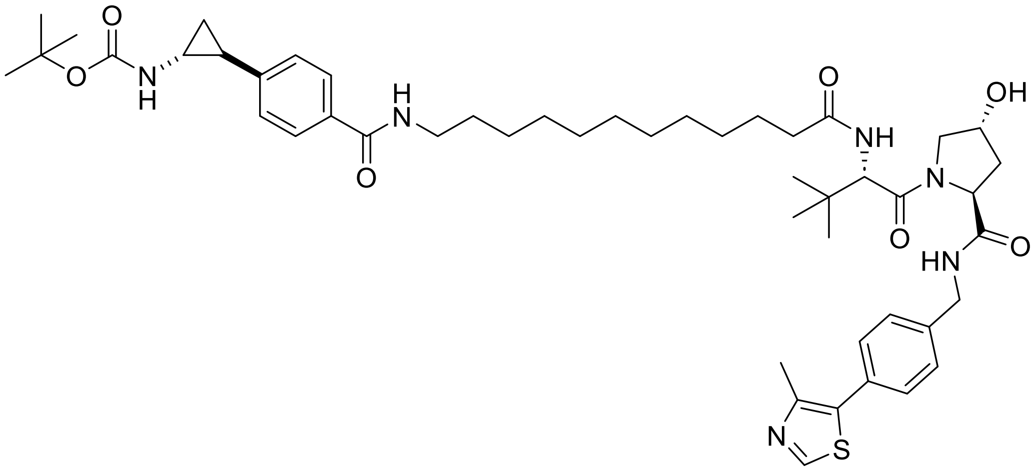

Tert-butyl ((trans)-2-(4-((12-(((S)-1-((2S,4R)-4-hydroxy-2-((4-(4-methylthiazol-5-yl)benzyl)carbamoyl)pyrrolidin-1-yl)-3,3-dimethyl-1-oxobutan-2-yl)amino)-12-oxododecyl)carbamoyl)phenyl)cyclopropyl)carbamate, 51: To a solution of **43** (0.0091 g, 0.0329 mmol, 1.1 eq.) in dry DMF (1 mL) at 0 °C under nitrogen, DIPEA (0.016 mL, 0.0898 mmol, 3.0 eq.) and HATU (0.0171 g, 0.0449 mmol, 1.5 eq.) were added. The resulting solution stirred for 15 mins, after which a solution of **50** (0.0188 g, 0.0299 mmol, 1.0 eq.) in dry DMF (1 mL) was added slowly. The reaction was allowed to warm to RT and was stirred for 18 h and monitored by TLC to completion. The reaction mixture was diluted with EtOAc (5 mL) and the organic phase was washed with saturated sodium hydrogen carbonate solution (2 x 5 mL) and brine (2 x 5 mL). The organic phase was dried over anhydrous sodium sulphate, filtered and concentrated *in vacuo* to yield the crude product as a brown oil. This was purified by silica column chromatography (5 % MeOH in DCM, then a second alumina column at 3 % MeOH in DCM) to yield **51** as an off-white solid (0.009 g, 34 %).

^{1}H NMR (400 MHz, Methanol-d_4) δ ppm 8.87 (s, 1 H), 7.71 (d, J=8.3 Hz, 2 H), 7.44 - 7.48 (m, 2 H), 7.39 - 7.43 (m, 2 H), 7.18 (d, J=8.3 Hz, 2 H), 4.63 (s, 1 H), 4.48 - 4.60 (m, 4 H), 4.35 (d, J=15.5 Hz, 1 H), 3.87 - 3.93 (m, 1 H), 3.77 - 3.83 (m, 1 H), 3.34 (t, J=7.2 Hz, 2 H), 2.65 - 2.74 (m, 1 H), 2.47 (s, 3 H), 2.17 - 2.34 (m, 3 H), 1.97 - 2.12 (m, 2 H), 1.54 - 1.66 (m, 4 H), 1.43 (s, 9 H), 1.25 - 1.39 (m, 16 H), 1.17 - 1.22 (m, 2 H), 1.03 (s, 9 H).

^{13}C NMR (101 MHz, Methanol-d_4) δ ppm 176.2, 174.6, 172.5, 170.2, 159.1, 153.0, 149.2, 147.0, 140.4, 133.6, 133.4, 131.7, 130.5, 129.1, 128.4, 127.2, 80.5, 71.2, 61.0, 59.1, 58.2, 43.8, 41.1, 39.1, 36.8, 36.7, 34.5, 30.76, 30.72, 30.66, 30.58, 30.46, 28.9, 28.2, 27.2, 27.2, 25.7, 17.1, 16.0.

HRMS (ESI): [M+Na]+ m/z calculated for $C_{49}H_{70}N_6O_7SNa$: 909.4924, found 909.4938, [M+H]+ m/z calculated for $C_{49}H_{71}N_6O_7S$: 887.5105, found 887.5517.

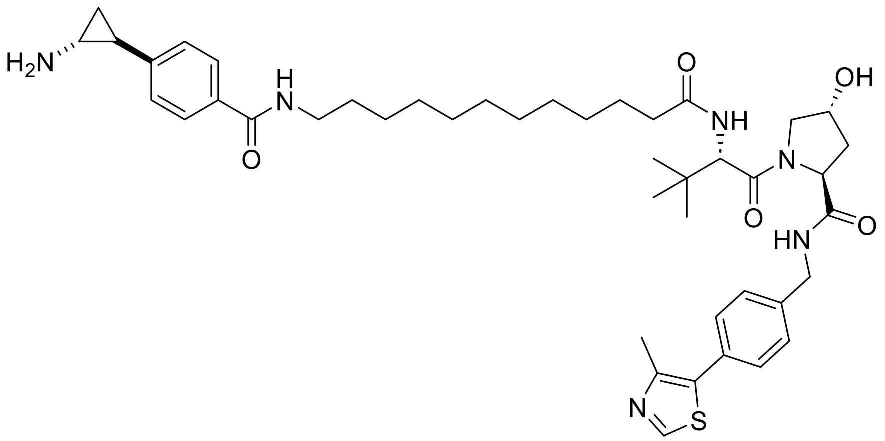

(2S,4R)-1-((2S)-2-(12-(4-((trans)-2-aminocyclopropyl)benzamido)dodecanamido)-3,3-dimethylbutanoyl)-4-hydroxy-N-(4-(4-methylthiazol-5-yl)benzyl)pyrrolidine-2-carboxamide, 52: **51** (0.009 g, 0.0101 mmol, 1.0 eq.) was dissolved in DCM (2 mL) with stirring under nitrogen. Trifluoroacetic acid (0.03 mL, 0.132 mmol, 13+ eq.) was added slowly and the reaction was stirred at RT for 3 h. The reaction was monitored by TLC to completion, then it was concentrated in vacuo. The residue was agitated in MeOH (5 mL) with MP-carbonate resin (0.1502 g, 3.02 mol/g) for 2.5 h, following which it was filtered and concentrated to yield **52** as an off-white solid (7.4 mg, 92.5%).

^{1}H NMR (400 MHz, Methanol-d_4) δ ppm 8.87 (s, 1 H), 7.68 (d, J=8.3 Hz, 2 H), 7.38 - 7.49 (m, 4 H), 7.10 (d, J=8.4 Hz, 2 H), 4.63 (s, 1 H), 4.47 - 4.59 (m, 3 H,), 4.35 (d, J=15.5 Hz, 1 H), 3.86 - 3.94 (m, 1 H), 3.77 - 3.84 (m, 1 H), 3.32 - 3.38 (m, 2 H), 2.51 (ddd, J=7.5, 4.5, 3.3 Hz, 1 H), 2.47 (s, 3 H), 2.17 - 2.33 (m, 3 H), 2.08 (ddd, J=13.5, 9.0, 4.6 Hz, 1 H), 1.91 (ddd, J=9.1, 5.9, 3.2 Hz, 1 H), 1.49 - 1.70 (m, 4 H), 1.25 - 1.37 (m, 14 H), 0.99 - 1.12 (m, 11 H).

^{13}C NMR (101 MHz, Methanol-d4) δ ppm 176.2, 174.6, 172.5, 170.2, 153.0, 149.2, 148.1, 140.4, 133.6, 133.0, 131.7, 130.5, 129.1, 128.4, 126.7, 71.2, 61.0, 59.1, 58.2, 43.8, 41.1, 39.1, 36.8, 36.7, 30.78, 30.75, 30.72, 30.66, 30.6, 30.5, 28.2, 27.18, 27.17, 26.6, 19.0, 16.0.

HRMS (ESI): [M+H]+ m/z calculated for $C_{44}H_{63}N_6O_5S$: 787.4581, found 787.4557.

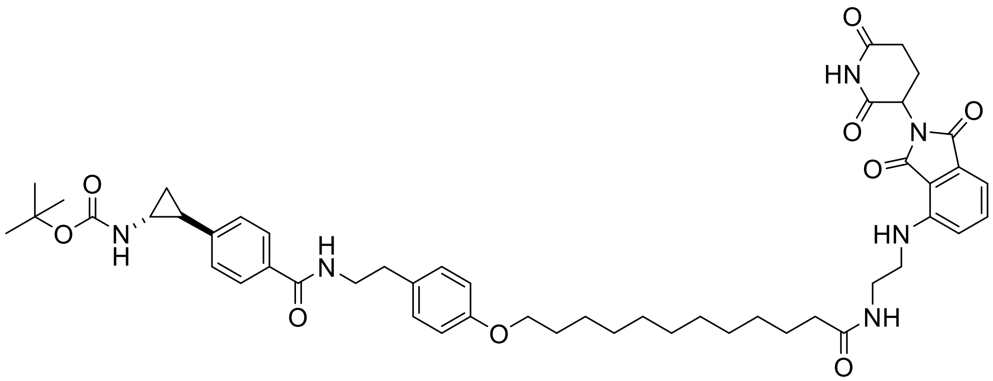

Tert-butyl ((trans)-2-(4-((4-((12-((2-((2-(2,6-dioxopiperidin-3-yl)-1,3-dioxoisoindolin-4-yl)amino)ethyl)amino)-12-oxododecyl)oxy)phenethyl)carbamoyl)phenyl)cyclopropyl) carbamate, 53: To a solution of **46** (0.020 g, 0.0336 mmol, 1.0 eq.) in dry DMF (3 mL) at 0 °C under nitrogen, DIPEA (0.02 mL, 0.1009 mmol, 3.0 eq.) and HATU (0.0192 g, 0.0504 mmol, 1.5 eq.) were added. The resulting solution stirred for 15 mins, after which **CRBN-NH2** (0.0119 g, 0.0336 mmol, 1.0 eq.) was added. The reaction was allowed to warm to RT and was stirred for 18 h and monitored by TLC to completion. The reaction mixture was concentrated *in vacuo*, diluted with water then washed with saturated sodium bicarbonate solution (2 x 10 mL) then brine (2 x 10 mL). The organic phase was dried over magnesium sulfate, concentrated *in vacuo*, then purified by silica column chromatography (5 % MeOH in DCM) to yield **53** as a yellow solid (0.023 g, 77 %).

^{1}H NMR (500 MHz, $CDCl_3$) δ ppm 8.46 (br s, 1 H), 7.59 (d, J=8.2 Hz, 2 H), 7.46 - 7.53 (m, 1 H), 7.07 - 7.18 (m, 5 H), 7.00 (d, J=8.5 Hz, 1 H), 6.84 (d, J=8.5 Hz, 2 H), 6.39 (br t, J=5.2 Hz, 1 H), 6.19 (br t, J=5.55 Hz, 1 H), 6.13 (br s, 1 H), 4.95 (br s, 1 H), 4.91 (dd, J=12.1, 5.2 Hz, 1 H), 3.93 (t, J=6.5 Hz, 2 H), 3.65 (q, J=6.7 Hz, 2 H), 3.40 - 3.51 (m, 3 H), 2.66 - 2.87 (m, 6 H), 2.16 (t, J=7.5 Hz, 2 H), 2.02 - 2.13 (m, 2 H), 1.76 (quin, J=6.7 Hz, 2 H), 1.60 (quin, J=5.6 Hz, 2 H), 1.39 - 1.49 (m, 11 H), 1.23 - 1.38 (m, 13 H), 1.17 - 1.22 (m, 2 H).

^{13}C NMR (126 MHz, $CDCl_3$) δ ppm 174.0, 171.1, 169.4, 168.5, 167.5, 167.2, 157.8, 146.8, 144.7, 136.3, 132.4, 132.2, 130.7, 129.7, 126.9, 126.4, 116.8, 114.7, 111.9, 110.2, 77.2, 68.0, 48.9, 42.1, 41.2, 39.1, 36.6, 34.7, 31.4, 29.7, 29.42, 29.41, 29.37, 29.29, 29.24, 29.20, 28.4, 25.9, 25.6, 22.7, 16.7.

HRMS (ESI): [M+Na]+ m/z calculated for $C_{36}H_{52}N_2O_6Na$: 617.3567, found 617.3570; [M+H]+ m/z calculated for $C_{36}H_{53}N_2O_6$: 595.3747, found 595.3754.

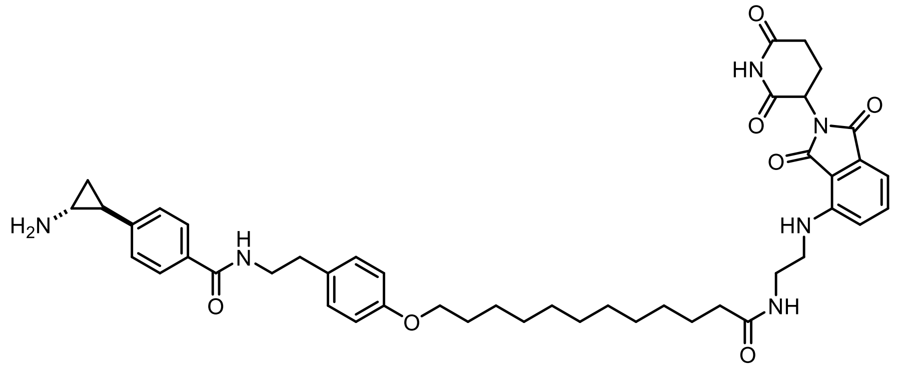

4-((Trans)-2-aminocyclopropyl)-N-(4-((12-((2-((2-(2,6-dioxopiperidin-3-yl)-1,3-dioxoisoindolin-4-yl)amino)ethyl)amino)-12-oxododecyl)oxy)phenethyl)benzamide, 54: 53 (0.023 g, 0.0258 mmol, 1.0 eq.) was dissolved in dichloromethane (3 mL) with stirring under nitrogen. HCl in 1,4-dioxane (4 M, 0.5 mL, excess) was added and the mixture stirred for 18 hours. The reaction was monitored by TLC to completion. The mixture was concentrated *in vacuo*, then purified by silica column chromatography (8 % methanol in DCM) to yield **54** as a yellow solid (0.0175 g, 82 %).

^{1}H NMR (500 MHz, Methanol-d_4) δ ppm 7.72 (d, J=8.3 Hz, 2 H), 7.53 (dd, J=8.1, 7.6 Hz, 1 H), 7.24 (d, J=8.4 Hz, 2 H), 7.14 (d, J=8.2 Hz, 2 H), 7.11 (br d, J=8.6 Hz, 1 H), 7.04 (d, J=7.1 Hz, 1 H), 6.82 (d, J=8.6 Hz, 2 H), 5.04 (dd, J=12.6, 5.5 Hz, 1 H), 3.92 (t, J=6.4 Hz, 2 H), 3.54 (t, J=7.4 Hz, 2 H), 3.44 (dd, J=13.1, 5.1 Hz, 4 H), 2.91 (dt, J=7.8, 4.0 Hz, 1 H), 2.79 - 2.86 (m, 3 H), 2.65 - 2.76 (m, 2 H), 2.41 (ddd, J=10.1, 6.5, 3.6 Hz, 1 H), 2.16 (t, J=7.4 Hz, 2 H), 2.08 (dtd, J=12.6, 5.3, 5.3, 2.4 Hz, 1 H), 1.72 (quin, J=6.5 Hz, 2 H), 1.53 - 1.62 (m, 2 H), 1.36 - 1.50 (m, 4 H), 1.21 - 1.35 (m, 12 H).

^{13}C NMR (126 MHz, Methanol-d_4) δ ppm 177.1, 174.8, 171.7, 170.7, 169.8, 169.4, 159.3, 148.3, 143.9, 137.4, 134.4, 134.1, 132.6, 130.9, 128.8, 127.6, 118.2, 115.7, 112.2, 111.6, 69.1, 50.3, 42.99, 42.98, 39.9, 37.3, 35.8, 32.5, 32.4, 30.8, 30.72, 30.68, 30.58, 30.56, 30.4, 27.3, 27.1, 24.0, 22.6, 14.5.

HRMS (ESI): [M+Na]+ m/z calculated for $C_{45}H_{56}N_6O_7Na$: 815.4108, found 815.4108; [M+H]+ m/z calculated for $C_{45}H_{57}N_6O_7$: 793.4289, found 793.4285.

2. Supplementary Information: Biology

2.1. LSD-HDAC1-CoREST expression and purification

The grid below shows the buffers used in Purification.

Lysis Buffer (Buffer A)<br>• 50 mM Tris/Cl pH 7.5<br>• 50 mM Potassium Acetate<br>• 5 % v/v Glycerol<br>• 0.4 % v/v Triton X-100	Wash Buffer (Buffer B)<br>• 50 mM Tris/Cl pH 7.5<br>• 50 mM Potassium Acetate<br>• 5 % v/v Glycerol
Cleavage Buffer (Buffer C)<br>• 50 mM Tris/Cl pH 7.5<br>• 50 mM Potassium Acetate<br>• 5 % v/v Glycerol<br>• 0.5 mM tris(2-carboxyethyl)phosphine (TCEP)	Gel Filtration Buffer (Buffer D)<br>Filtered at vacuum.<br>• 50 mM Tris/Cl pH 7.5<br>• 50 mM Potassium Acetate<br>• 0.5 mM TCEP

The CoREST ternary complex was comprised of full length native LSD1 (UniProt ID: O60341), full length HDAC1 (UniProt ID: Q13547) and N-terminally truncated RCOR1 (86-485aa) (UniProt ID: Q9UKL0). Protein constructs were cloned into pcDNA3 vector. The RCOR1 constructs contained an N-terminal (His)10(Flag)3 tag followed by a Tev protease cleavage site. The constructs for ternary complex were co-transfected into suspension-grow HEK 293F cells (Thermofisher Scientific) with polyethylenimine (PEI) (Sigma) and harvested after 48 hours. This section of the procedure was carried out as described previously in literature.[11,12]

Cell pellets from 1.2 L culture were defrosted, and buffer A (30 mL) was added. The solution was sonicated (Soniprep 150) in an beaker of ice using a 9 mm probe at 10 ampules; operated with 10

seconds on, 30 seconds off for 3 cycles. The resulting suspension was split into two Beckman Tubes (topped up with buffer A to make equal weight) and centrifugation was used to remove insoluble materials by being spun at 18000 rpm for 25 mins at 4 °C (108000 × *g*, Avanti™ J-30I, Beckman Coulter, Rotor JA30.50). The resulting supernatant was loaded onto a resin (Anti-Flag M2 affinity gel, Sigma Aldrich, 1 mL per 1.2 L culture) and placed on a tube roller for 30 minutes at 4 °C. The loaded resin was pelleted (4000 rpm, 10 mins, 4 °C) and subsequently washed with buffer B (3 x 13 mL) and buffer C (4 x 13 mL), having being re-suspended and centrifuged (4000 rpm, 2 mins, 4 °C) each time. The loaded resin was then taken up in buffer C (5 mL). Tev protease (30 µL, 1.2 mg/mL) was added and cleaved overnight on a roller at 4 °C.

After Tev cleavage, the resin was pelleted (4000 rpm, 10 mins, 4 °C) and the supernatant was concentrated by centrifugation (4000 rpm, 4 °C) using a 4 ml Amicon®Ultra centrifugal filter (Merck Millipore) with a membrane nominal molecular weight cut off of 10 kDa, until 500 µL remained and further filtered using a 0.22 µm centrifugal filter (Merck Millipore).

Finally, the complex was purified by gel filtration, *via.* size exclusion chromatography. This was achieved on a pre-packed Superose 6 (10/300) GL column (GE Healthcare) in buffer D. The column was equilibrated in buffer D for 3 hr (0.1 mL/min). Fractions were collected in a 96 well plate (0.5 mL per fraction). Fractions containing pure complex, determined SDS-PAGE gel, were pooled and concentrated.

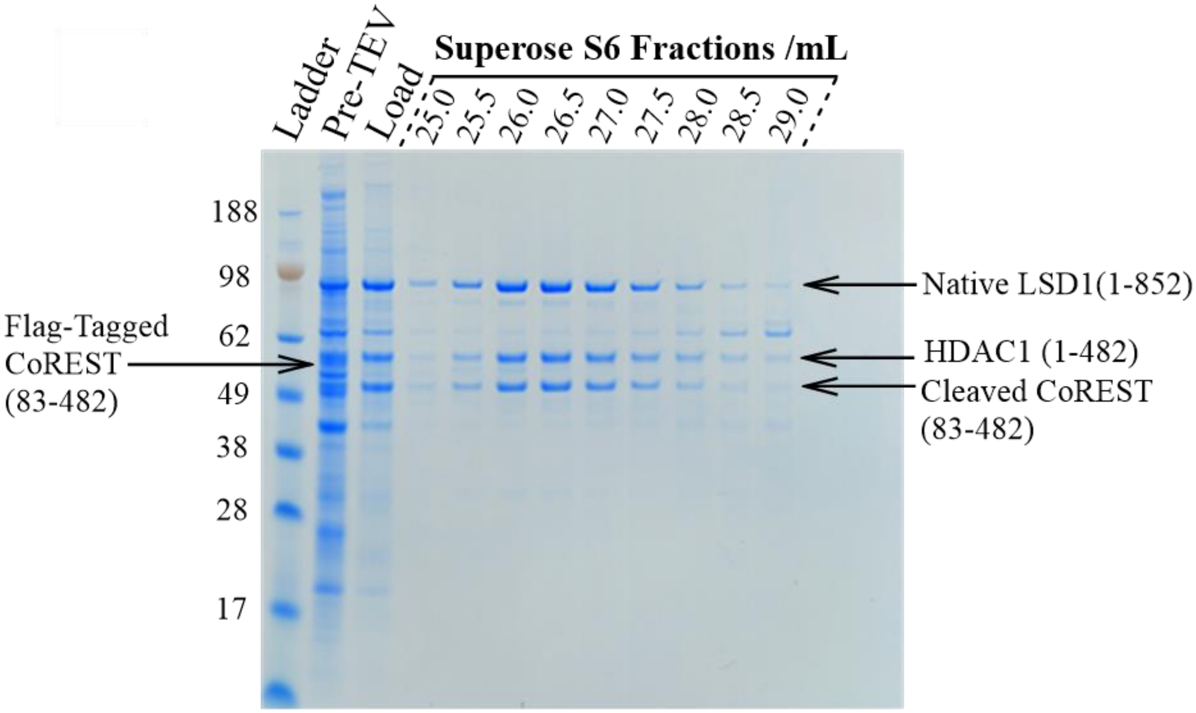
Ladder
Pre-TEV
Load
Superose S6 Fractions /mL
25.0
25.5
26.0
26.5
27.0
27.5
28.0
28.5
29.0
188
98
62
49
38
28
17
Flag-Tagged CoREST (83-482)
Native LSD1(1-852)
HDAC1 (1-482)
Cleaved CoREST (83-482)

2.2. SP2509/SP2577 analogues LSD1 HRP-coupled demethylase assay protocol

The commercial reagents for the assay were HRP and Amplex® Ultrared, which are available to purchase from ThermoFisher (31490) and Invitrogen (10737474), respectively. HRP was made up to 8 mg/mL stock solution in 1xPBS buffer, divided into 10 µL aliquots, snap-frozen in liquid nitrogen and stored at -80 °C. Amplex® Ultrared was made up to 10 mM solution in DMSO, divided into 20 µL aliquots and wrapped in foil, away from light and stored at -20 °C. For each assay experiment, CoREST complex was diluted to 500 nM in assay buffer (20 mM HEPES pH 7.5 and 50 mM NaCl). HRP was defrosted by diluting each aliquot in 90 µL 1xPBS solution, making a 0.8 mg/mL solution. Amplex® Ultrared was made up to 200 µM in assay buffer. HRP and Amplex® Ultrared were pre-mixed before addition to the plate. $H3K4Me_2$ substrate (4 mM in deionised water) was diluted to 300 µM in assay buffer. All stocks were stored on ice during assay preparation.

The assay was carried out using a 'black well' 96 well plate (Corning no. 3915) in a reaction volume of 100 µL. The final concentrations in the assay were as follows: 50 nM CoREST complex, 10 µM Amplex® UltraRed, 0.04 mg/mL HRP and 15 µM $H3K4Me_2$ peptide substrate. The final DMSO concentration in each well was 1.1 %. Three wells of each experiments were designated 'background controls' and contained 85 µL assay buffer, premixed Amplex® UltraRed and HRP (10 µL) and H3K4Me peptide substrate (300 µM, 5 µL).

A stock solution of inhibitor (1-5 mM in DMSO) was subjected to an inhibitor dilution series, in Eppendorf tubes, in 100 % DMSO. Each new inhibitor stock (4 µL) was diluted in assay buffer (1 in 75) within a new Eppendorf set with assay buffer (20 mM HEPES pH 7.5 and 50 mM NaCl), ensuring the percentage of DMSO was consistent throughout the series. To each well of interest, Inhibitor in assay buffer (75 µL) was added, and CoREST complex (500 nM, 10 µL) was added thereafter. The plate was placed on a rocker and pre-incubated for 30 mins. To each well, premixed Amplex® UltraRed and HRP (10 µL) was added before $H3K4Me_2$ peptide substrate in assay buffer solution (300 µM, 5 µL) was added to the wall of the plate. The plate was covered in foil and spun down for one minute before being placed in the plate reader. The final percentage concentration of DMSO in each well was 1.1 %.

The data were measured with an excitation wavelength of 530 nm and an emission wavelength of 590 nm on a Victor X5 plate reader (Perkin Elmer) over 21 minutes which were used to generate a final

‘Fluorescence Change’ reading for each well, after subtraction of the ‘background fluorescence’ reading. Data were processed in Microsoft Excel 2013 (v 15.0) and dose response cures and IC_{50} values were determined with GraphPad Prism 9.

<u>Figure S1. SP2509/SP2577 analogues dose response curves</u>

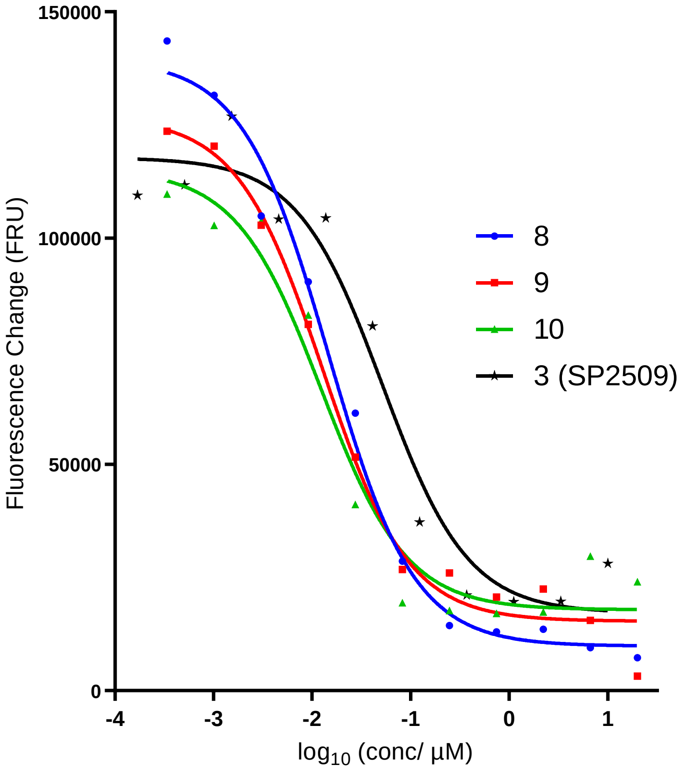

8: 15 nM

9: 13 nM

10: 12 nM

Range of measurement:

0 – 20 µM

3 (SP2509): 52 nM

Range of measurement:

0 – 10 µM

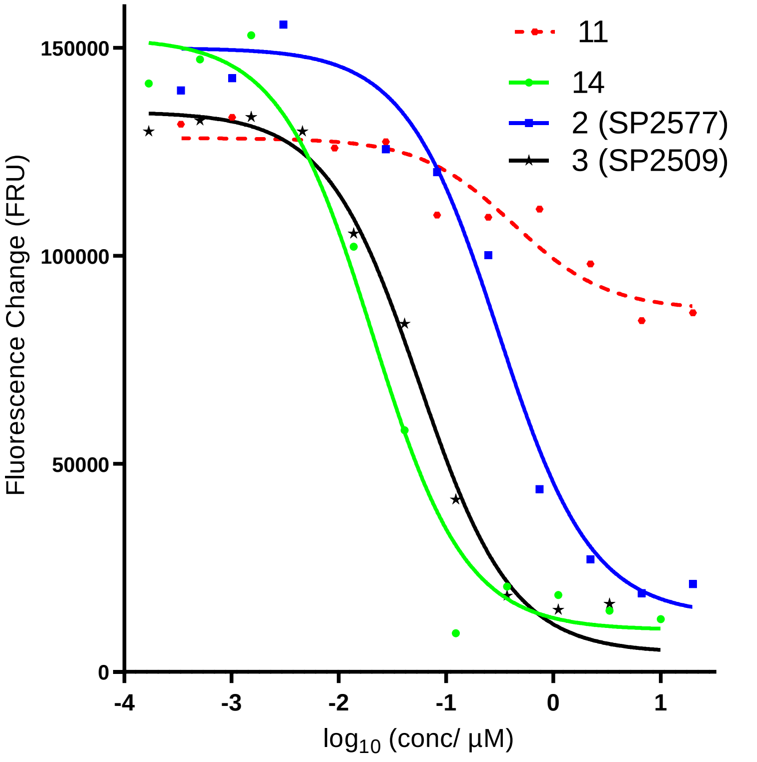

11: >20 µM

2: (SP2577): 306 nM

Range of measurement:

0 – 20 µM

14: 21 nM

3: (SP2509): 56 nM

Range of measurement:

0 – 10 µM

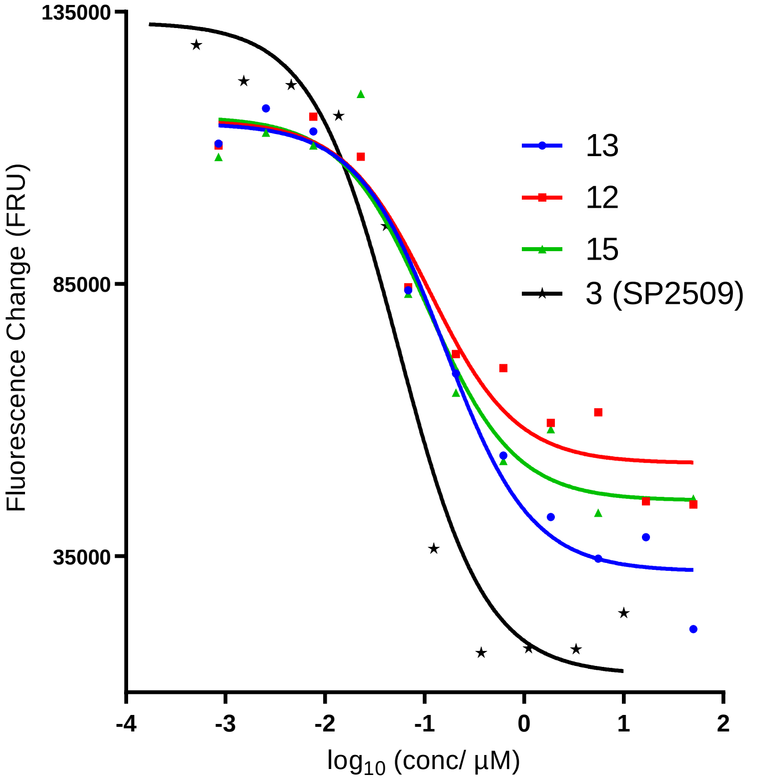

13: 159 nM

12: 113 nM

15: 108 nM

Range of measurement:

0 – 50 µM

3 (SP2509): 55 nM

Range of measurement:

0 – 10 µM

2.3. TCP analogues LSD1 HRP-coupled demethylase assay protocol

Components and concentrations for the HRP-coupled demethylase assay

Component	Stock concentration	Initial dilution	Final concentration (per well)
Horseradish peroxidase (HRP)	8 mg/mL in PBS	In PBS to 0.8 mg/mL, then mixed 1:1 with Amplex solution	0.04 mg/mL
Amplex UltraRed	10 mM in DMSO	In assay buffer to 200 µM, then mixed 1:1 with HRP solution	10 µM
CoREST complex	Concentration variable, in 25 mM Tris/Cl pH 7.5 with 50 mM Kac, 0.5 mM TCEP and 25% glycerol	In assay buffer to 500 nM	50 nM
H3 peptide (21 aa, H3K4me or H3K4me2)	4 mM in deionised water	In assay buffer to 300 µM	15 µM
DMSO	-	-	1% in control wells
Compounds	Variable in DMSO	-	Variable, with DMSO volume of 1%
Assay buffer (20 mM HEPES pH 7.5, 50 mM NaCl, 10 mg/mL BSA	-	-	-

The assay components are shown at their final concentrations per well in the table above. CoREST complex (HDAC 1/RCOR1/LSD1) was added to the assay buffer alongside either DMSO or the relevant inhibitor at the desired concentration on black-walled 96 well plates. Alongside this was another control, with DMSO but no CoREST or inhibitor. Plates were spun to 3000 rpm between each addition of a reagent. Plates were incubated at 20 °C for 30 mins, following which HRP/Amplex Ultrared (1:1 mixture) and peptide was added. Plates were spun, then read on an PerkinElmer 2030 Explorer plate reader, with excitation of 530 nm and absorption of 590 nm, with 7 readings taken over 24 minutes.

Components, where bought in, are listed below:

HRP – Pierce™ Horseradish Peroxidase, Invitrogen

H3K4me2 peptide – Histone H3 dimethyl lysine-4 peptide, Enzo Life Sciences

Amplex UltraRed, ThermoFisher Scientific

To the assay buffer was added HRP/AMP in each well to concentrations equal to those in above, following which hydrogen peroxide solution (diluted from 3% hydrogen peroxide solution into a 20 mM stock) was added, in a 1 in 3 dilution series with final concentrations starting from 3 mM. Negative control wells contained either 100% buffer or as above with no hydrogen peroxide solution.

Figure S2. TCP analogues dose response curves

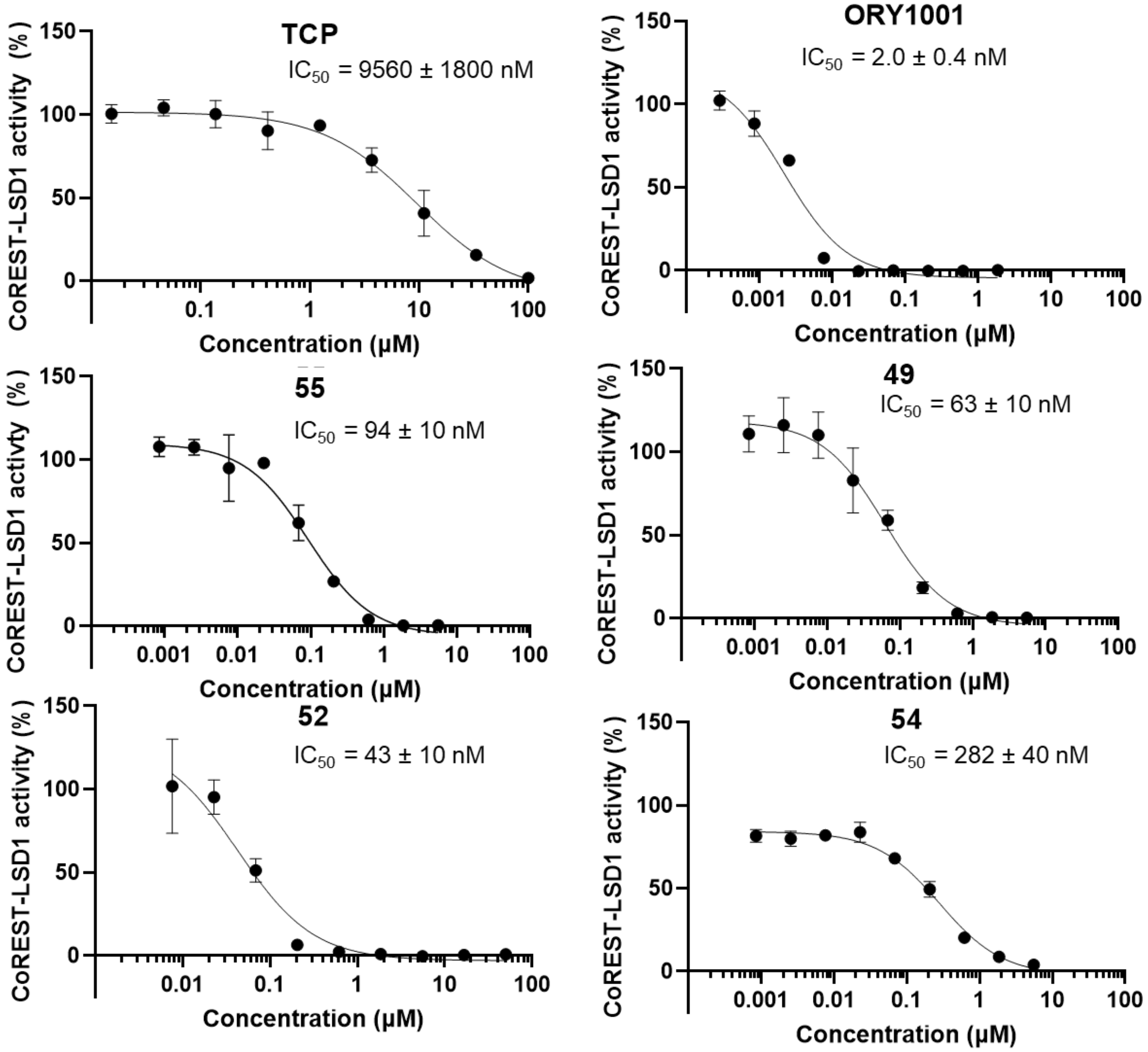

2.4. CTG cell viability assays

HCT116 cells were seeded onto 96-well plates at a density of 2000 cells/well and allowed 24 h to settle. Cells were then treated with LSD1 inhibitors (0.0041-10 uM) alongside WT, DMSO and cell free control wells for 24-48 h. After this time, plates were equilibrated to RT for 30 mins, then CellTiter-Glo (Promega, G9242) reagent (5 uL/100 uL media) was added. Cells were lysed on an orbital shaker for 10 mins, then incubated at RT for 2 mins to allow luminescence to stabilise, following which luminescence was recorded on a HiDEX Sense microplate reader. To obtained plotted values, luminescence of each tested well was divided by that of the DMSO control, which were plotted against concentration using GraphPad Prism. Standard linear regression was applied to obtain apparent IC_{50} values where possible.

Figure S3. Cell viability dose response curves

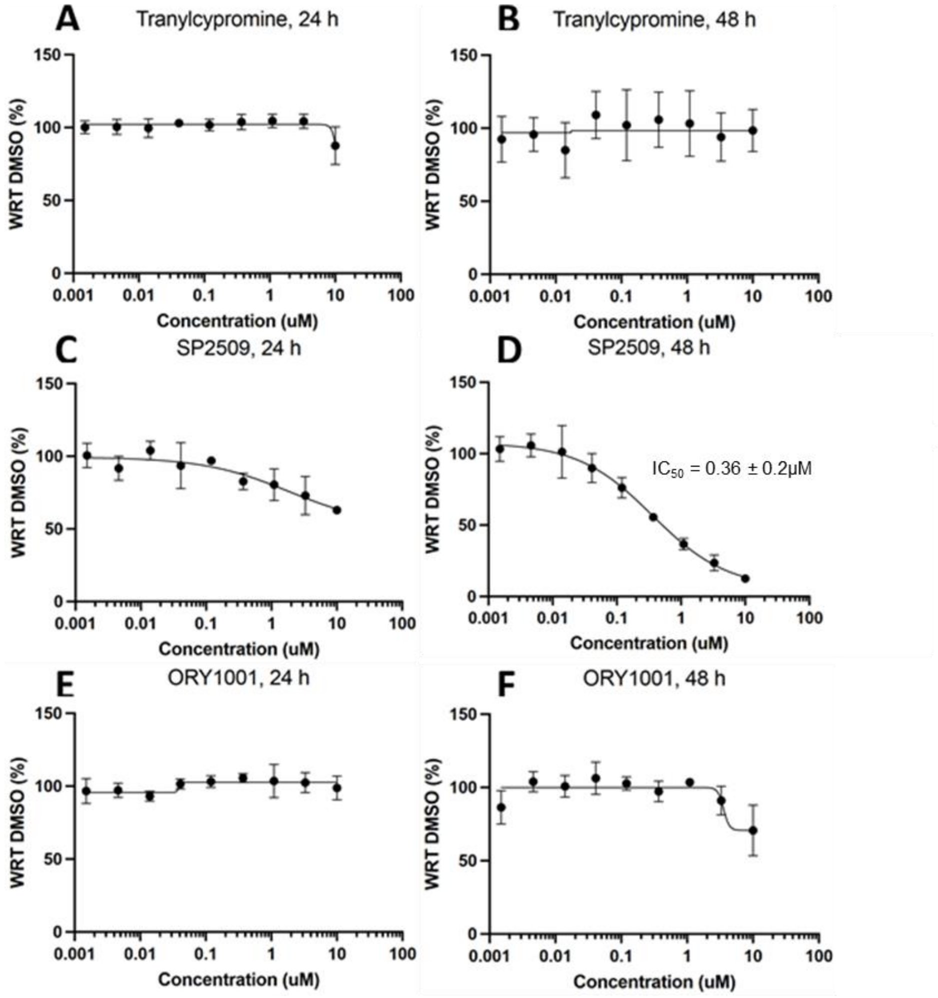

2.5 Protocols for LSD1 degradation and histone methylation in HCT116 cells

HCT116 human colon carcinoma cells were grown in Dulbecco's Modified Eagle Medium (DMEM) supplemented with Fetal Bovine Serum (FBS, 10% final concentration) and 1x glutamine/penicillin/streptomycin. Cells were incubated at 37 °C and 5% CO_2. Cells were treated with compounds (0.1-20 μM) with a final DMSO volume of 0.04%. DMSO (0.04%) treated and untreated (WT) cells were included as controls.

HCT116 cells were seeded into six well plates (400000 cells per well) and after requisite compound treatment time (24h, 48h, 72h) cells were harvested and either snap-frozen in liquid nitrogen or lysed in lysis buffer (50mM Tris-HCl, 150 mM NaCl, 0.5% NP-40, 0.5% Triton X-100 + protease inhibitor) on ice for 30 mins. This was centrifuged (15000 rpm, 4 °C, 15 mins) and the supernatant collected. H_2SO_4 (0.4 M, equal volume) was added to the pellets for histone extraction and the tubes incubated overnight at 4 °C. Following this, the histone extract was centrifuged (15000 rpm, 4 °C, 15 mins) and the supernatant collected. Protein concentrations of whole cell extract were quantified via Bradford Assay using BIO-RAD Protein Assay Dye Reagent Concentrate.

Western blots were run on NuPAGE 4-12% Bis-Tris gels, with 30 μg of protein (whole cell extract) or an equivalent volume of histone extract loaded per lane, with all extracts dyed with 4x NuPAGE LDS Sample Buffer. PageRuler Plus Prestained Ladder was used as a reference. Gel electrophoresis was carried out at 140 V for 60-90 mins, following which the proteins were transferred onto nitrocellulose membrane at 30 V for 60 mins. Membranes were blocked in LI-COR Intercept Blocking Buffer overnight at RT on a roller, following which the membrane was probed with primary antibodies for 1 h at RT. The membrane was washed with PBS + 0.1% Tween (3 x 10 mins), then incubated with secondary antibodies for 50 mins at RT. The membrane was washed again (PBS Tween 2 x 10 mins, PBS 1 x 10 mins) following which the blot was visualised with the Odyssey Infrared Imaging System. Image processing and quantification was performed with ImageStudio Lite.

Antibodies used:
a-tubulin (mouse) – Sigma, t5168 (1:10000 dilution)
LSD1 (rabbit) – Abcam, ab129195 (1:10000 dilution)
H3K4me2 (rabbit) – EpiCypher 13-0027 SnapChip Certified (1:1000)

2.6 Western blots for LSD1 degradation and histone methylation with SP2509/SP2577 analogues – 24h

Figure S4

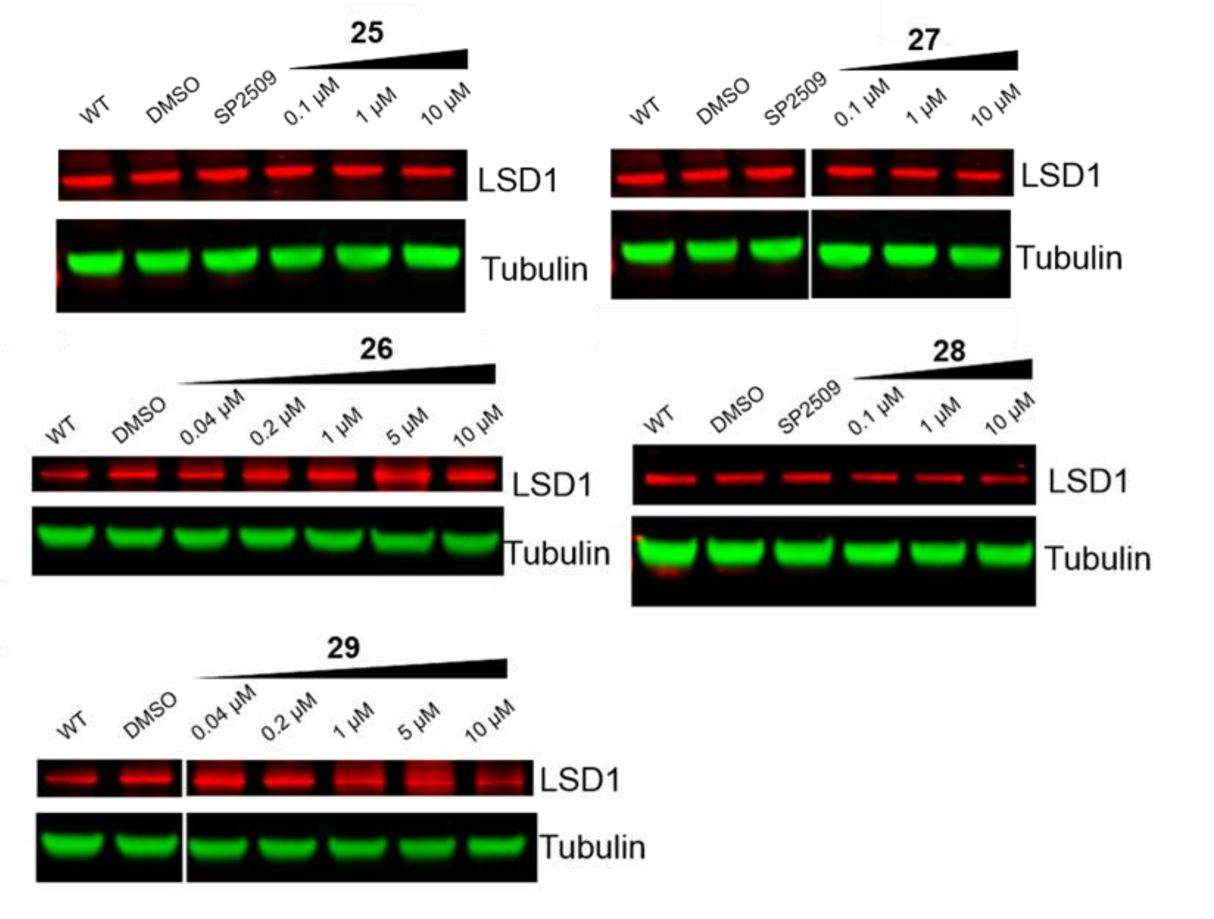

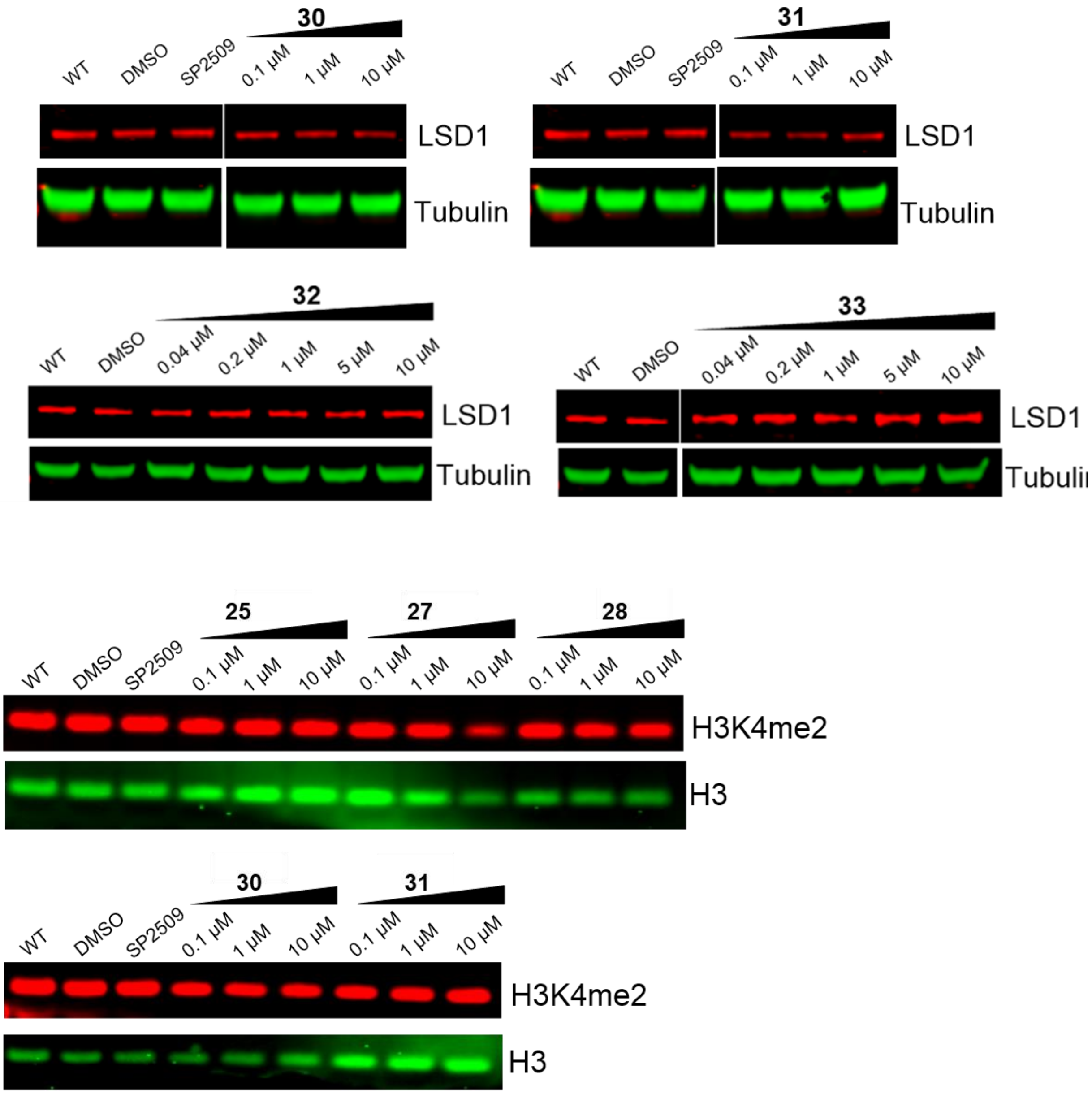
30
WT
DMSO
SP2509
0.1 μM
1 μM
10 μM
LSD1
Tubulin
31
WT
DMSO
SP2509
0.1 μM
1 μM
10 μM
LSD1
Tubulin
32
WT
DMSO
0.04 μM
0.2 μM
1 μM
5 μM
10 μM
LSD1
Tubulin
33
WT
DMSO
0.04 μM
0.2 μM
1 μM
5 μM
10 μM
LSD1
Tubulin
25
27
28
WT
DMSO
SP2509
0.1 μM
1 μM
10 μM
0.1 μM
1 μM
10 μM
0.1 μM
1 μM
10 μM
H3K4me2
H3
30
31
WT
DMSO
SP2509
0.1 μM
1 μM
10 μM
0.1 μM
1 μM
10 μM
H3K4me2
H3

2.7 Western blots for LSD1 degradation and histone methylation with TCP analogues – 24h

Figure S5

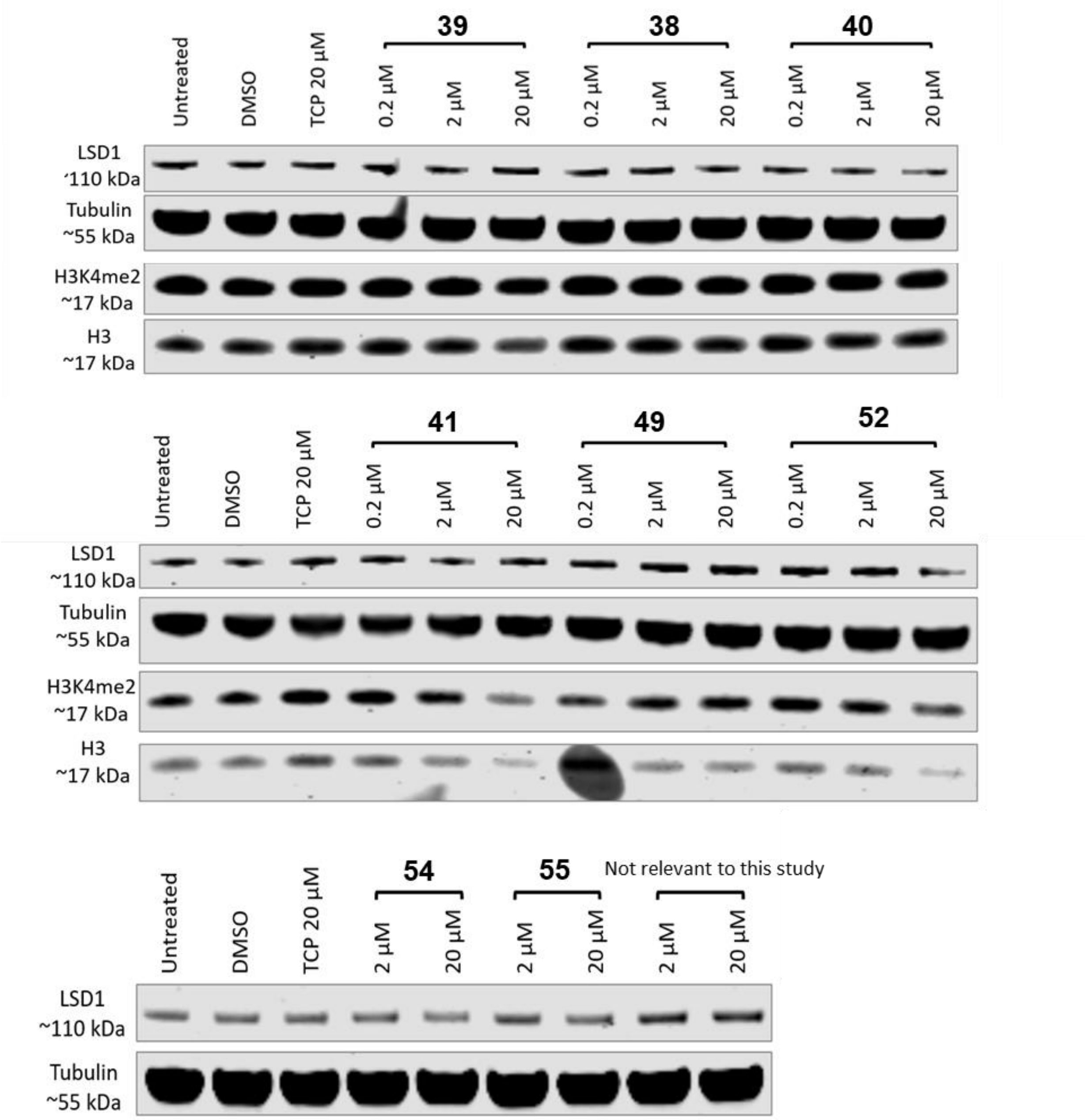

2.8 Western blots for LSD1 degradation and histone methylation with selected TCP analogues – 48h and 72h

Figure S6

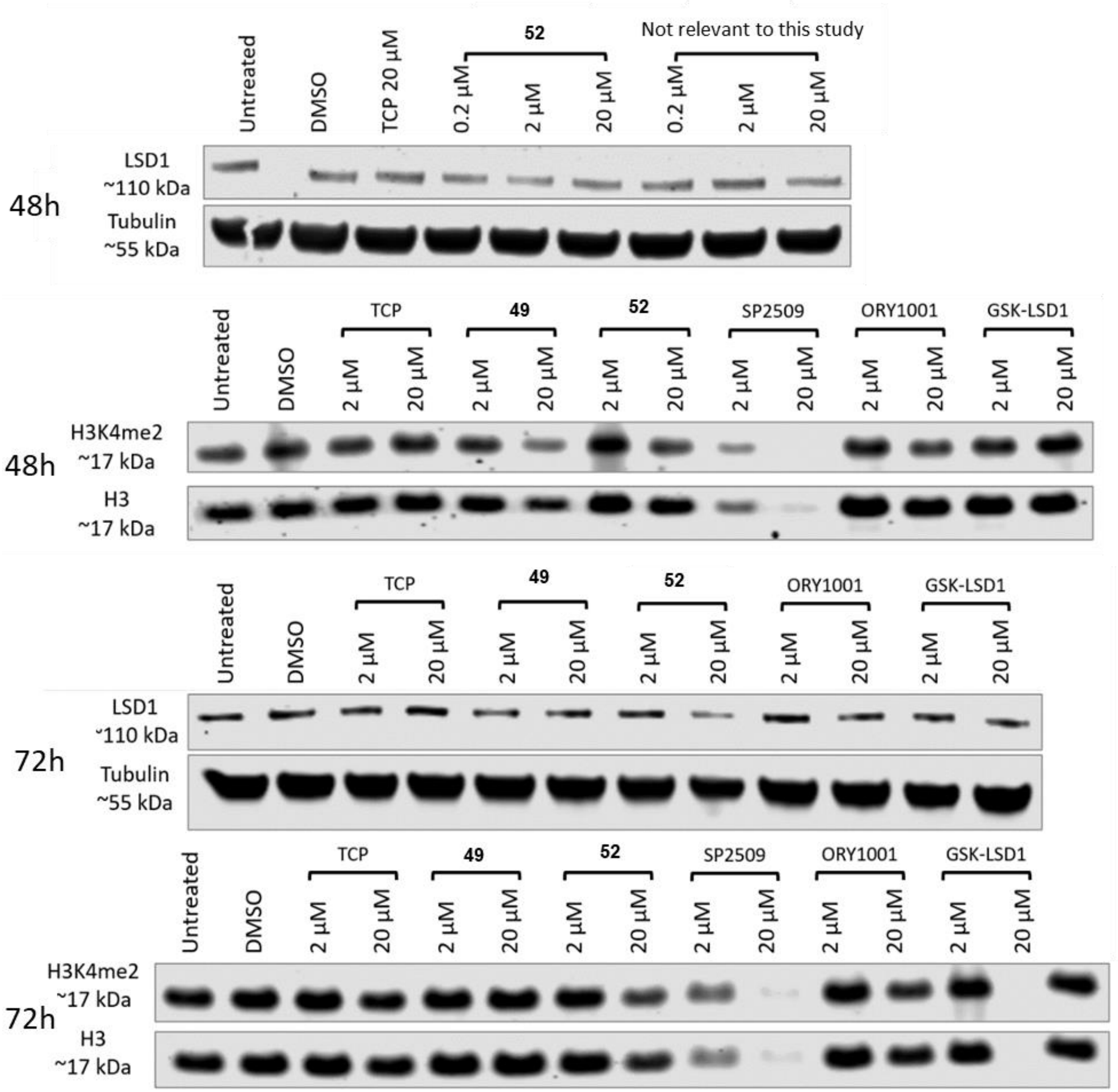

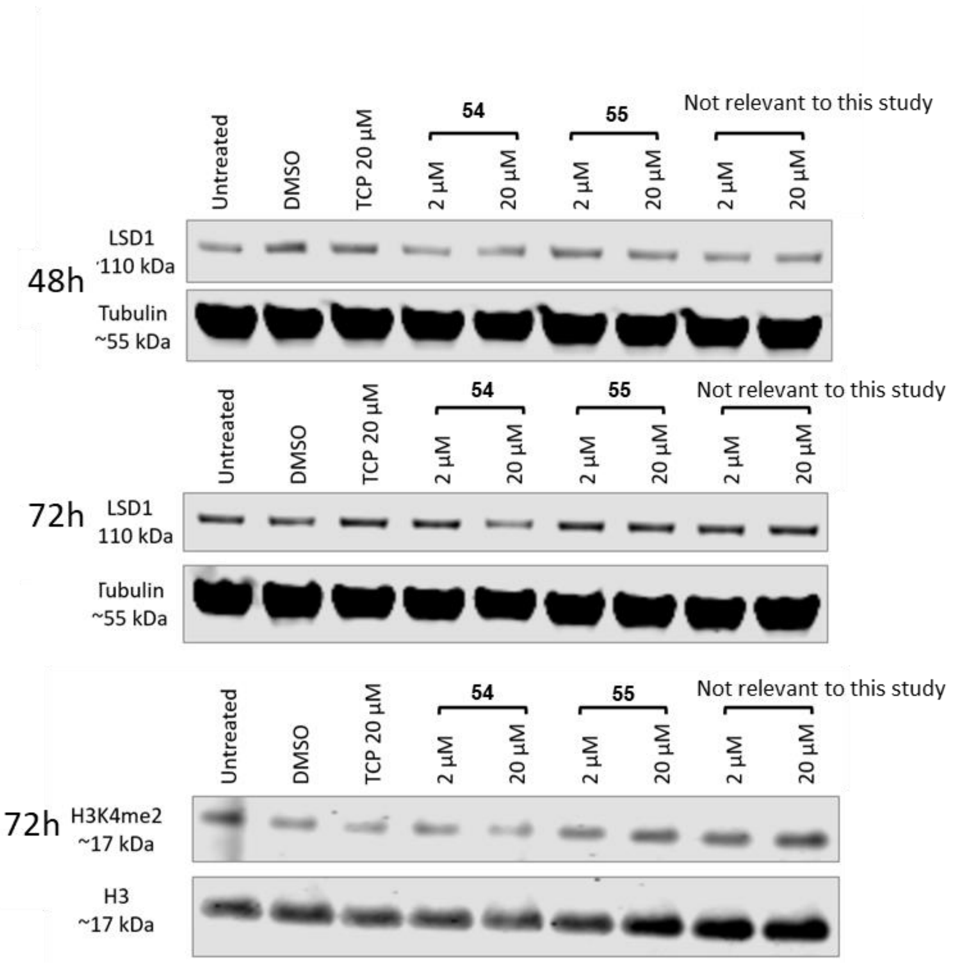
48h
Untreated
DMSO
TCP 20 µM
54
2 µM
20 µM
55
2 µM
20 µM
Not relevant to this study
2 µM
20 µM
LSD1
110 kDa
Tubulin
~55 kDa
72h
LSD1
110 kDa
Tubulin
~55 kDa
72h
H3K4me2
~17 kDa
H3
~17 kDa

3. References

1 Y. Zhou, Y. Li, W. J. Wang, P. Xiang, X. M. Luo, L. Yang, S. Y. Yang and Y. L. Zhao, *Bioorganic Med. Chem. Lett.*, 2016, **26**, 4552–4557.

2 F. Zhang, D. Zheng, L. Lai, J. Cheng, J. Sun and J. Wu, *Org. Lett.*, 2018, **20**, 1167–1170.

3 L. Y. Li, J. Di Peng, W. Zhou, H. Qiao, X. Deng, Z. H. Li, J. D. Li, Y. D. Fu, S. Li, K. Sun, H. M. Liu and W. Zhao, *Eur. J. Med. Chem.*, 2018, **148**, 359–371.

4 United States Patent Office, US 2009/0036420 A1, 2009, 1–75.

5 M. D. Varney, G. P. Marzoni, C. L. Palmer, J. G. Deal, S. Webber, K. M. Welsh, R. J. Bacquet, C. A. Bartlett, C. A. Morse, C. L. J. Booth, S. M. Herrmann, E. F. Howland, R. W. Ward and J. White, *J. Med. Chem.*, 1992, **35**, 663–676.

6 L. Peng, Z. Zhang, C. Lei, S. Li, Z. Zhang, X. Ren, Y. Chang, Y. Zhang, Y. Xu and K. Ding, *ACS Med. Chem. Lett.*, 2019, **10**, 767–772.

7 C. Jacquot, C. M. McGinley, E. Plata, T. R. Holman and W. A. Van Der Donk, *Org. Biomol. Chem.*, 2008, **6**, 4242–4252.

8 T. Aiba, M. Sato, D. Umegaki, T. Iwasaki, N. Kambe, K. Fukase and Y. Fujimoto, *Org. Biomol. Chem.*, 2016, **14**, 6672–6675.

9 M. Teresa Borrello, H. Benelkebir, A. Lee, C. Hin Tam, M. Shafat, S. A. Rushworth, K. M. Bowles, L. Douglas, P. J. Duriez, S. Bailey, S. J. Crabb, G. Packham and A. Ganesan, *ChemMedChem*, 2021, **16**, 1316–1324.

10 C. Binda, S. Valente, M. Romanenghi, S. Pilotto, R. Cirilli, A. Karytinos, G. Ciossani, O. A. Botrugno, F. Forneris, M. Tardugno, D. E. Edmondson, S. Minucci, A. Mattevi and A. Mai, *J. Am. Chem. Soc.*, 2010, **132**, 6827–6833.

11 Y. Song, L. Dagil, L. Fairall, N. Robertson, M. Wu, T. J. Ragan, C. G. Savva, A. Saleh, N. Morone, M. B. A. Kunze, A. G. Jamieson, P. A. Cole, D. F. Hansen and J. W. R. Schwabe, *Cell Rep.*, 2020, **30**, 2699–2711.

12 J. H. Kalin, M. Wu, A. V. Gomez, Y. Song, J. Das, D. Hayward, N. Adejola, M. Wu, I. Panova, H. J. Chung, E. Kim, H. J. Roberts, J. M. Roberts, P. Prusevich, J. R. Jeliazkov, S. S. Roy Burman, L. Fairall, C. Milano, A. Eroglu, C. M. Proby, A. T. Dinkova-Kostova, W. W. Hancock, J. J. Gray, J. E. Bradner, S. Valente,

A. Mai, N. M. Anders, M. A. Rudek, Y. Hu, B. Ryu, J. W. R. Schwabe, A. Mattevi, R. M. Alani and P. A. Cole, *Nat. Commun.*, 2018, **9**, 53.

Appendix: Analytical data for the final compounds

Compound **25**

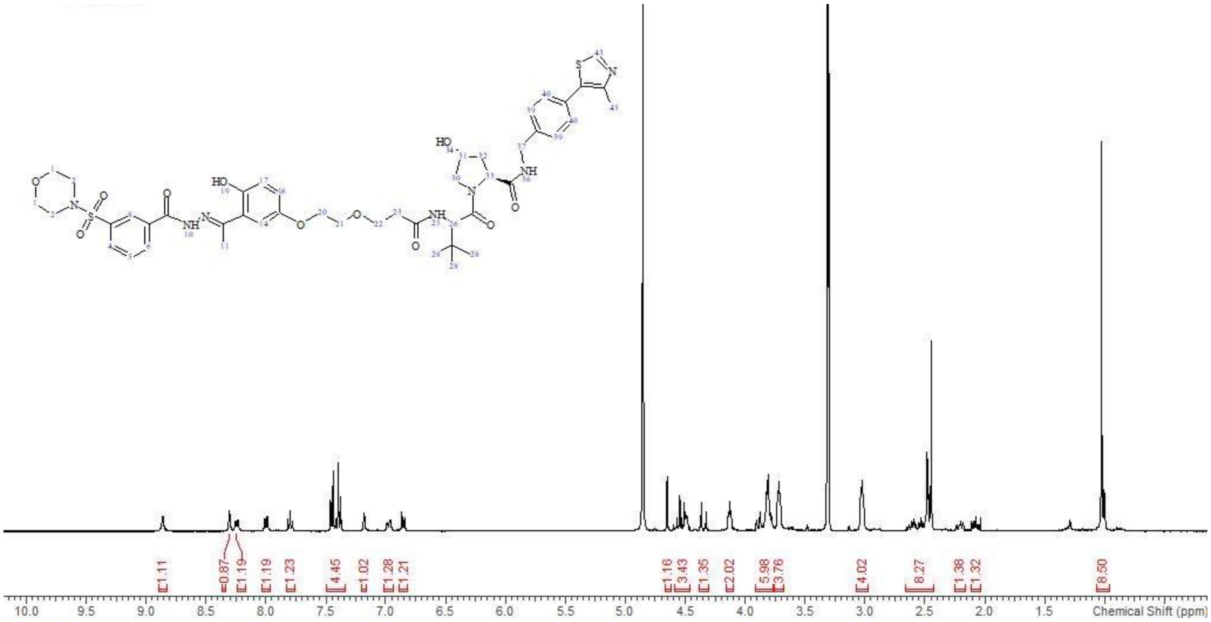

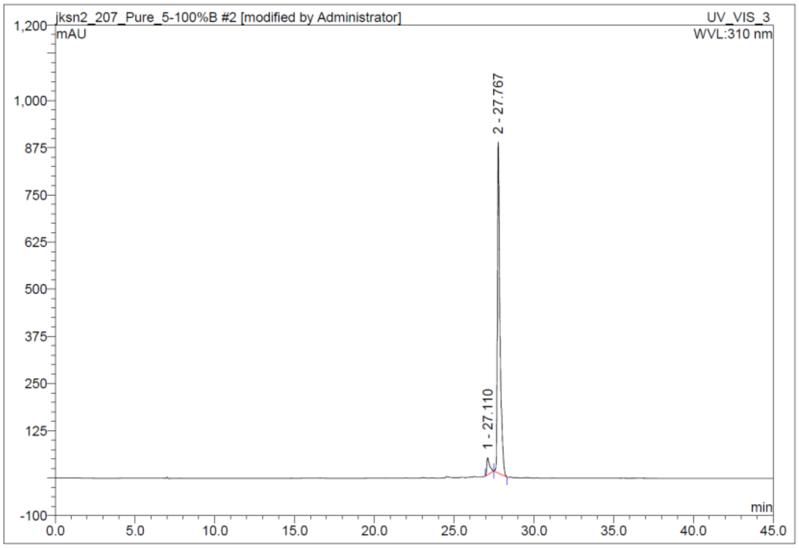

No.	Ret.Time min	Peak Name	Height mAU	Area mAU*min	Rel.Area %	Amount	Type
1	27.11	n.a.	44.480	9.008	5.25	n.a.	BMb
2	27.77	n.a.	878.192	162.476	94.75	n.a.	bMB
Total:			922.672	171.483	100.00	0.000	

Compound **26**

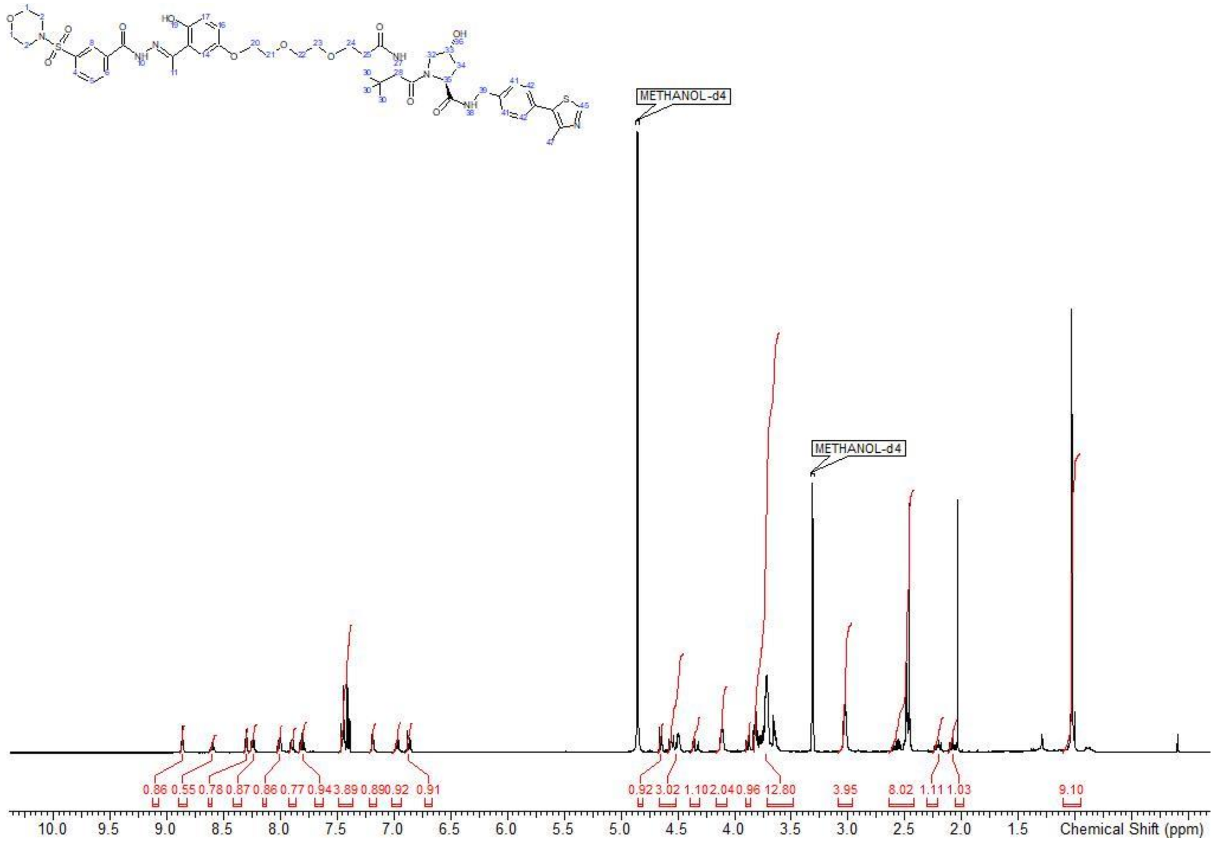

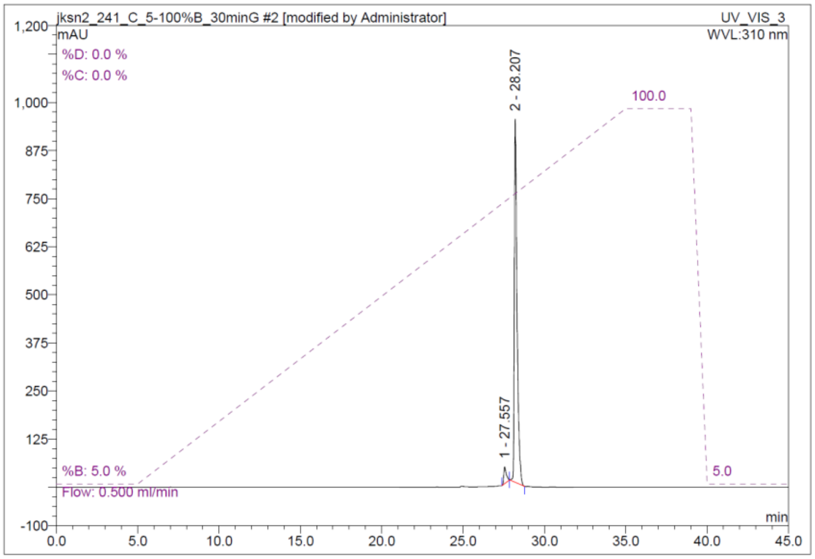

No.	Ret.Time min	Peak Name	Height mAU	Area mAU*min	Rel.Area %	Amount	Type
1	27.56	n.a.	43.213	8.064	4.36	n.a.	BMb
2	28.21	n.a.	944.139	177.094	95.64	n.a.	bMB
Total:			987.352	185.159	100.00	0.000	

Compound **27**

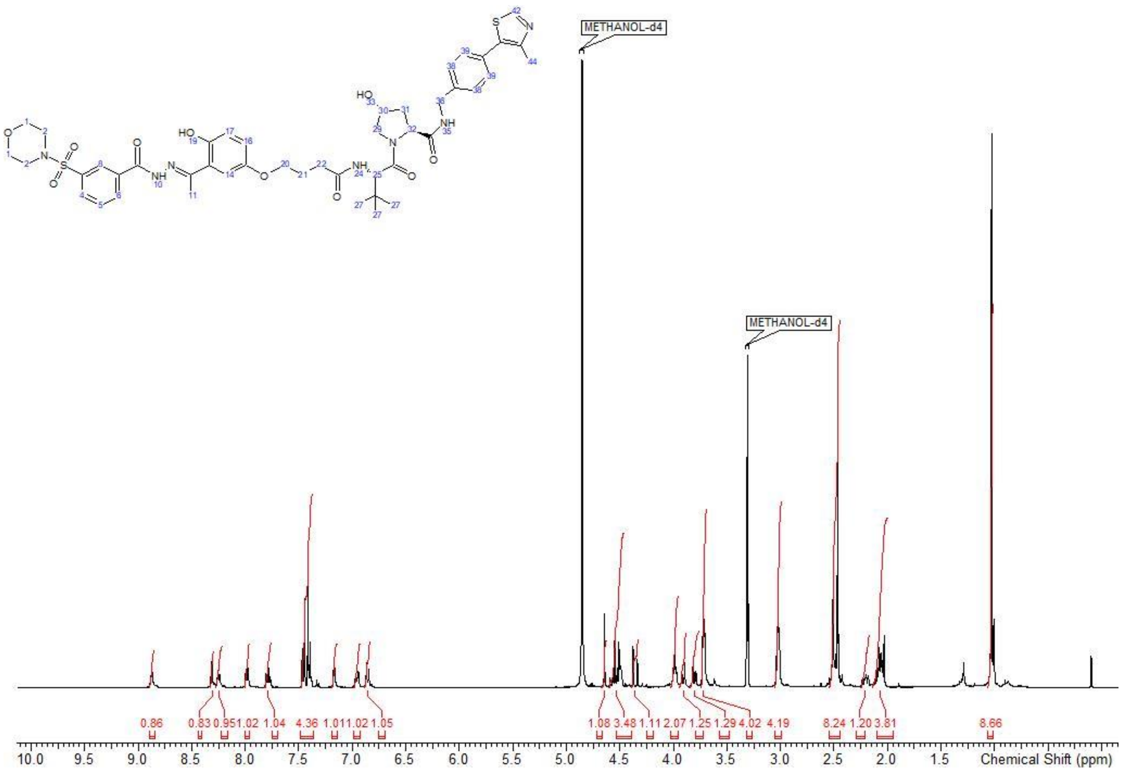

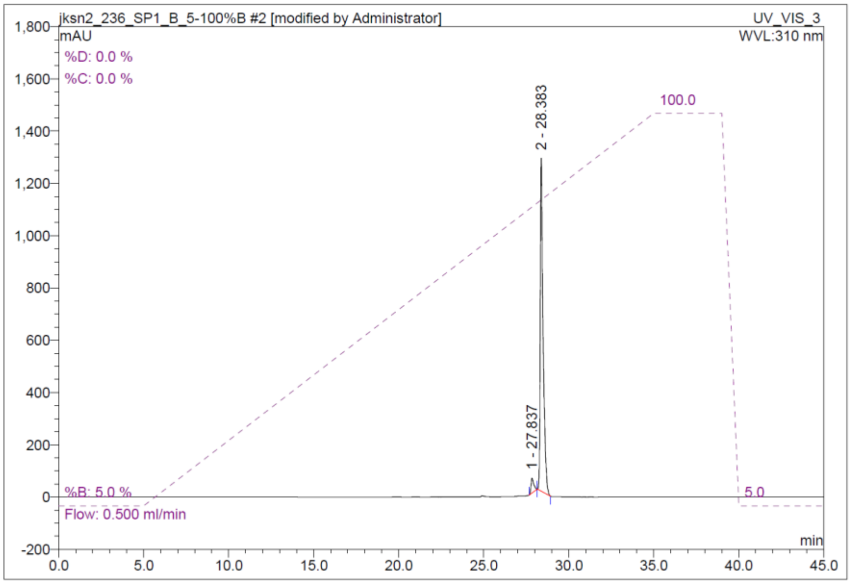

No.	Ret.Time min	Peak Name	Height mAU	Area mAU*min	Rel.Area %	Amount	Type
1	27.84	n.a.	56.919	10.887	4.14	n.a.	BMb
2	28.38	n.a.	1275.014	252.000	95.86	n.a.	bMB
Total:			1331.933	262.887	100.00	0.000	

Compound **28**

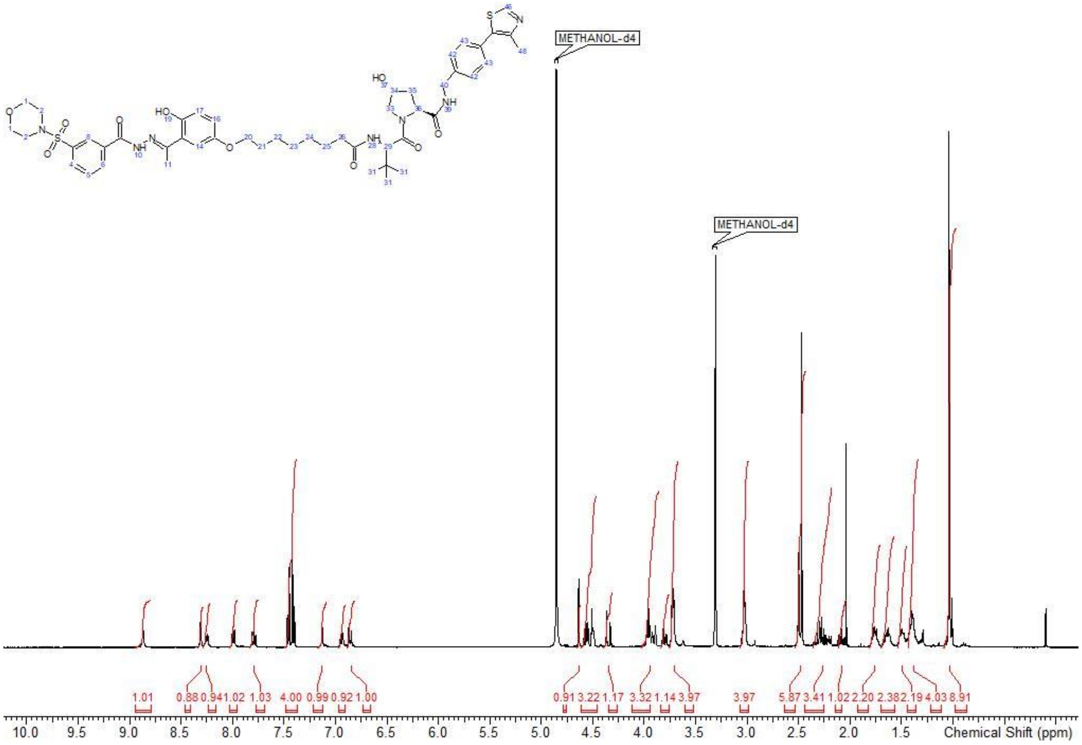

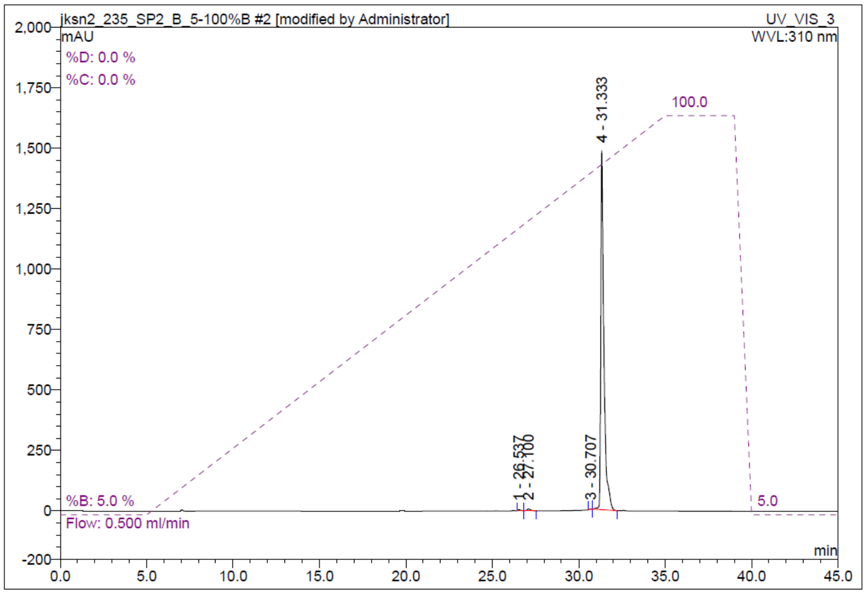

No.	Ret.Time min	Peak Name	Height mAU	Area mAU*min	Rel.Area %	Amount	Type
1	26.54	n.a.	5.104	0.900	0.28	n.a.	BMB
2	27.10	n.a.	7.681	1.799	0.57	n.a.	BMB
3	30.71	n.a.	1.115	0.181	0.06	n.a.	BMb
4	31.33	n.a.	1483.976	313.825	99.09	n.a.	bMB
Total:			1497.875	316.704	100.00	0.000	

Compound **29**

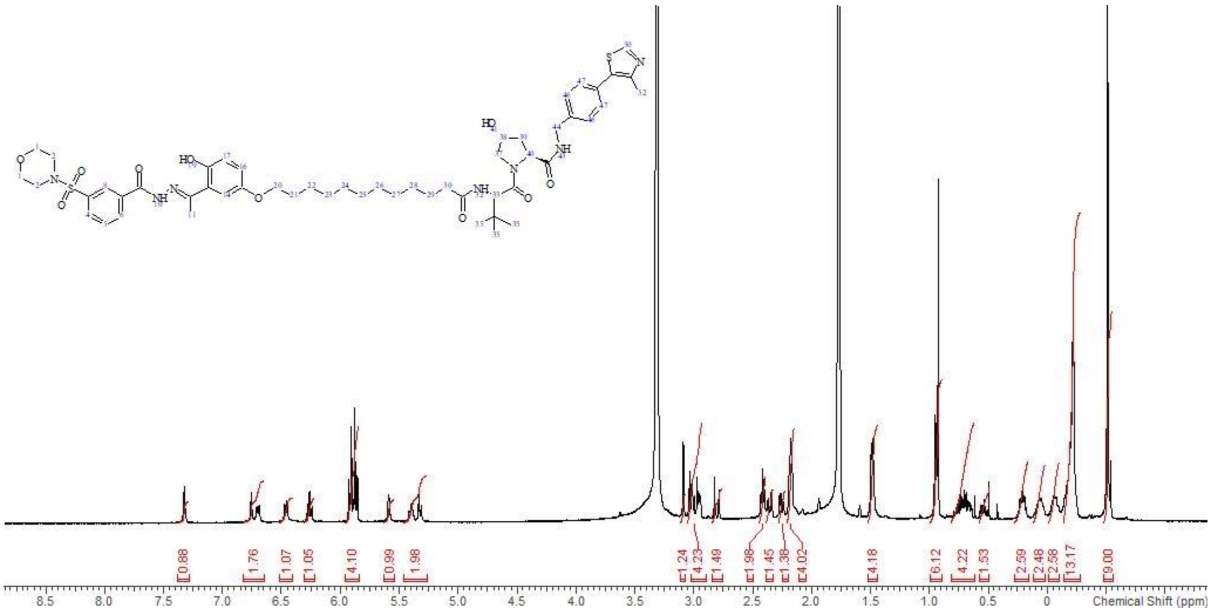

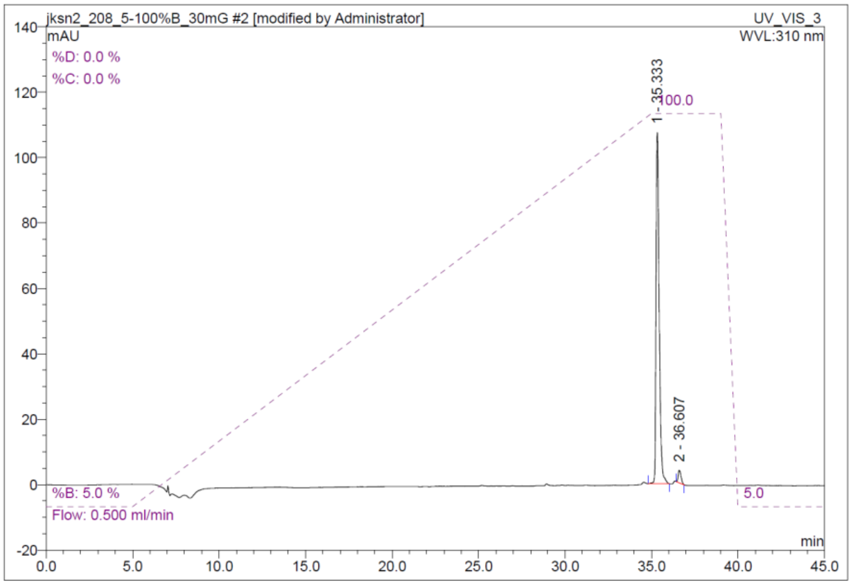

No.	Ret.Time min	Peak Name	Height mAU	Area mAU*min	Rel.Area %	Amount	Type
1	35.33	n.a.	107.375	22.259	96.85	n.a.	BMB*
2	36.61	n.a.	3.852	0.724	3.15	n.a.	BMB
Total:			111.227	22.983	100.00	0.000	

Compound **30**

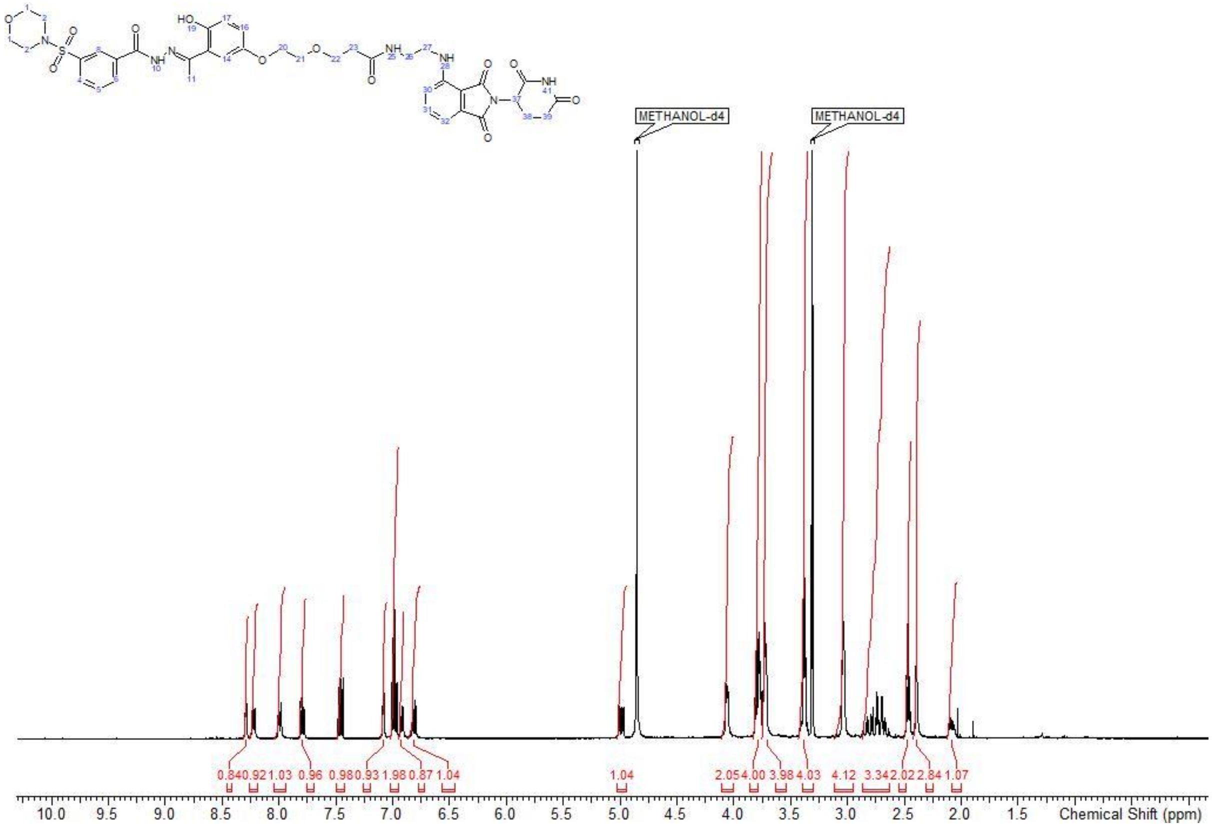

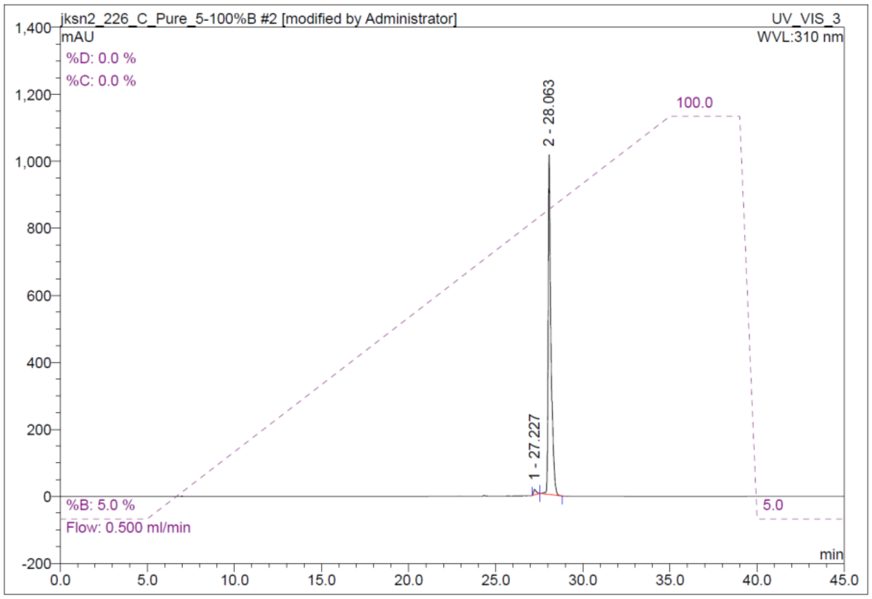

No.	Ret.Time min	Peak Name	Height mAU	Area mAU*min	Rel.Area %	Amount	Type
1	27.23	n.a.	16.821	3.079	1.58	n.a.	BMb
2	28.06	n.a.	1014.070	191.392	98.42	n.a.	bMB
Total:			1030.891	194.471	100.00	0.000	

Compound **31**

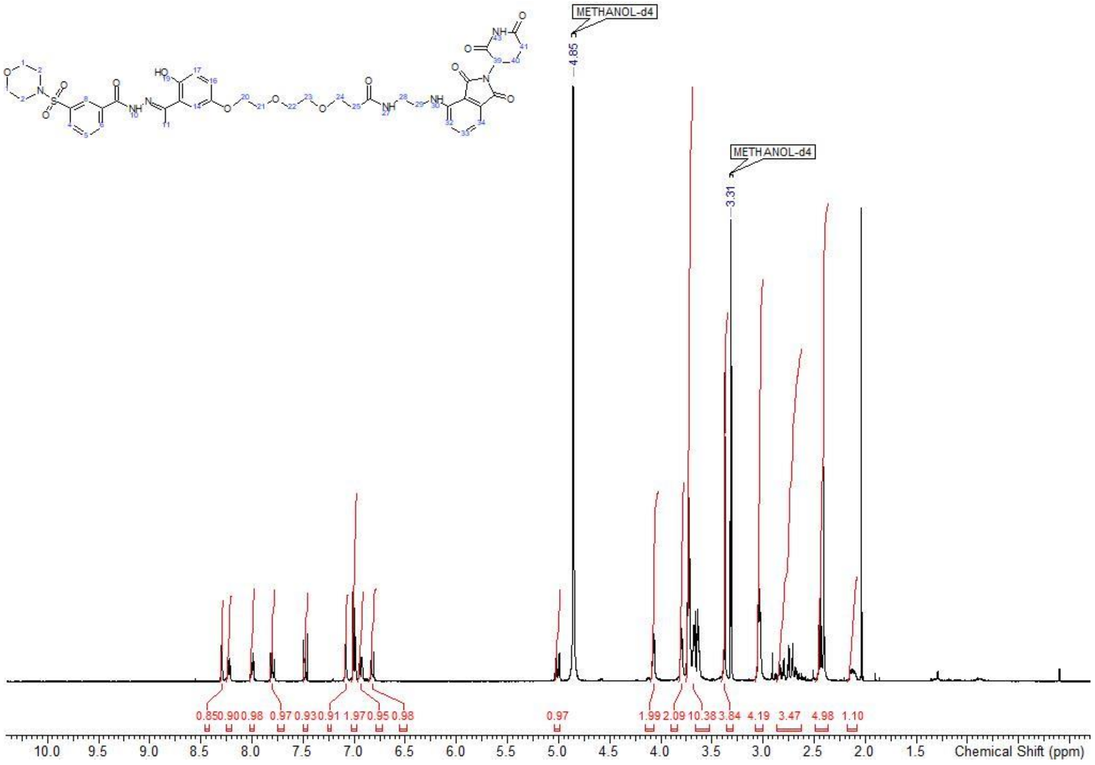

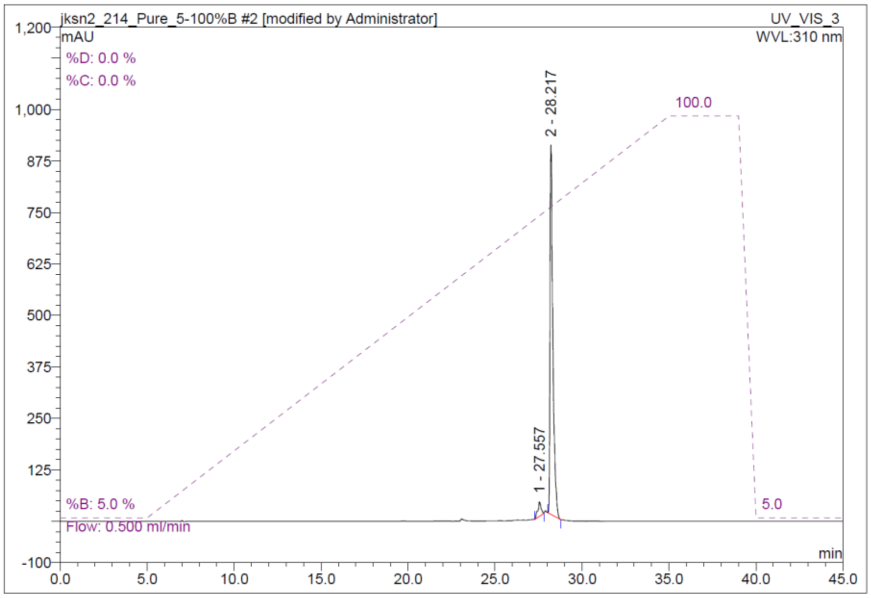

No.	Ret.Time min	Peak Name	Height mAU	Area mAU*min	Rel.Area %	Amount	Type
1	27.56	n.a.	34.725	7.570	4.36	n.a.	BMB*
2	28.22	n.a.	897.716	166.179	95.64	n.a.	BMB*
Total:			932.441	173.749	100.00	0.000	

Compound **32**

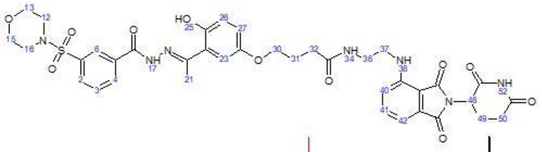

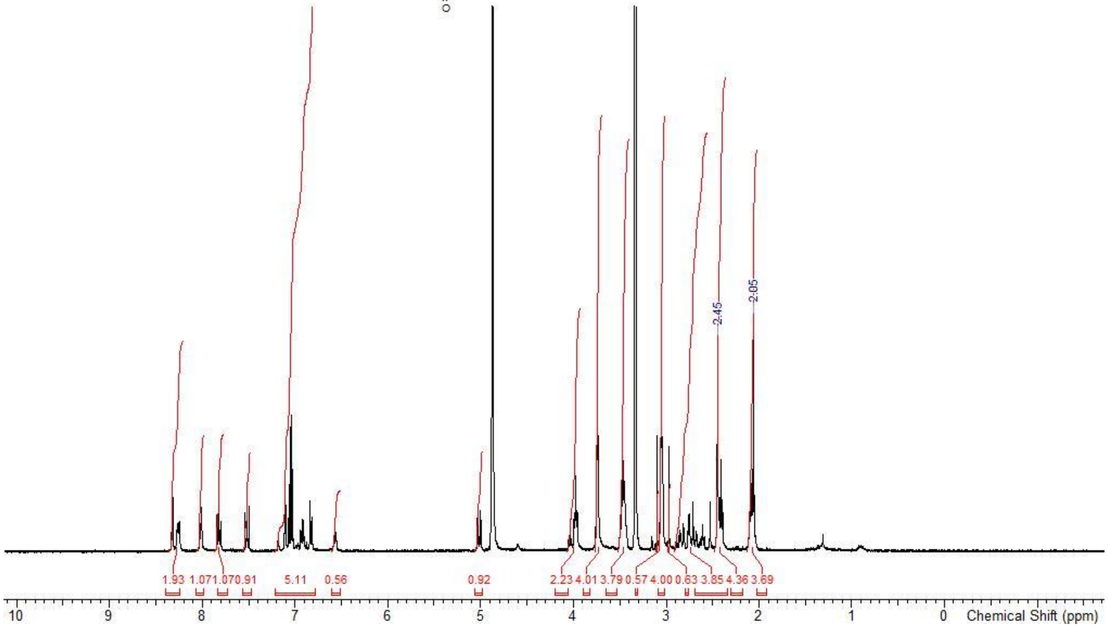

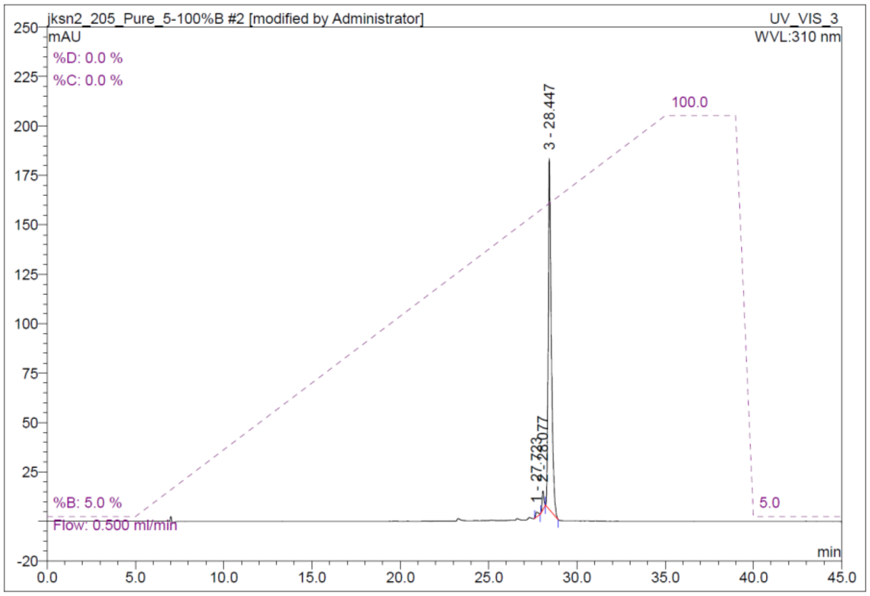

No.	Ret.Time min	Peak Name	Height mAU	Area mAU*min	Rel.Area %	Amount	Type
1	27.72	n.a.	2.515	0.416	1.11	n.a.	BMB
2	28.08	n.a.	9.606	1.166	3.11	n.a.	BMb
3	28.45	n.a.	177.661	35.872	95.78	n.a.	bMB
Total:			189.782	37.454	100.00	0.000	

Compound **33**

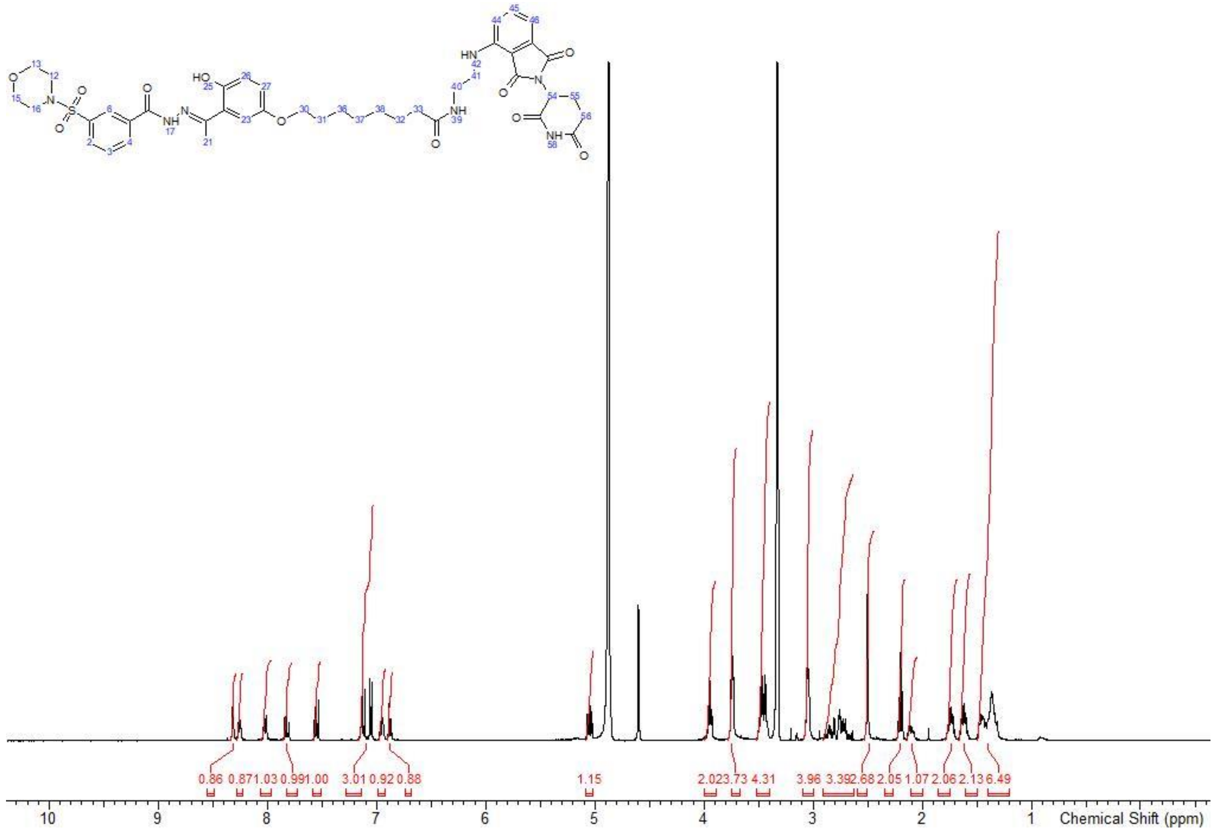

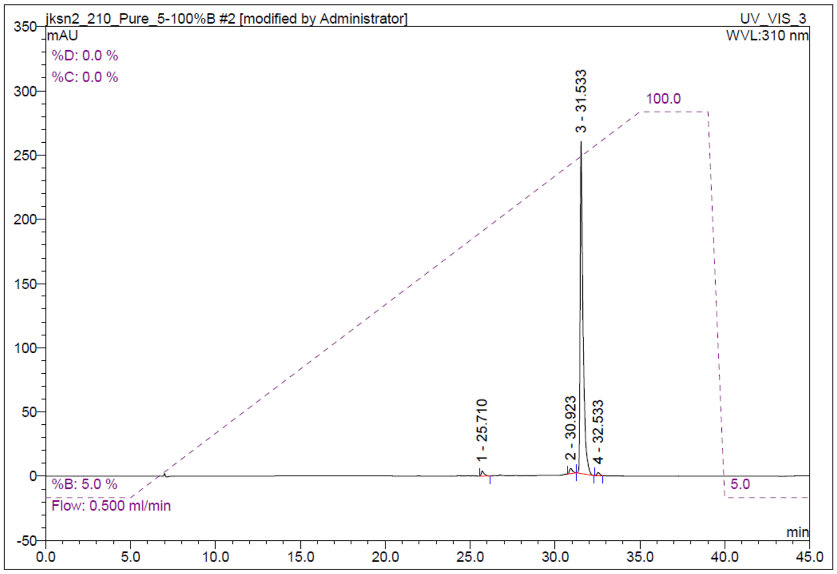

No.	Ret.Time min	Peak Name	Height mAU	Area mAU*min	Rel.Area %	Amount	Type
1	25.71	n.a.	3.899	0.773	1.45	n.a.	BMB
2	30.92	n.a.	4.035	0.803	1.50	n.a.	BMb
3	31.53	n.a.	258.562	51.407	96.18	n.a.	bMB
4	32.53	n.a.	2.520	0.467	0.87	n.a.	BMB
Total:			269.016	53.450	100.00	0.000	

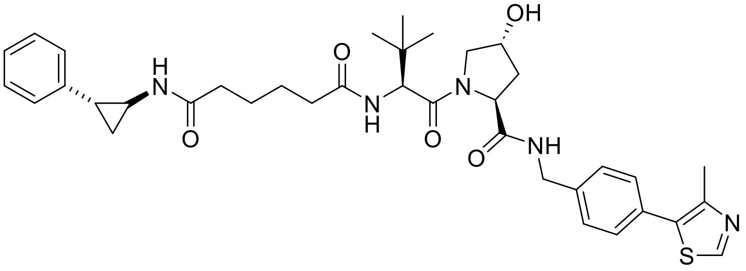

Compound **38**

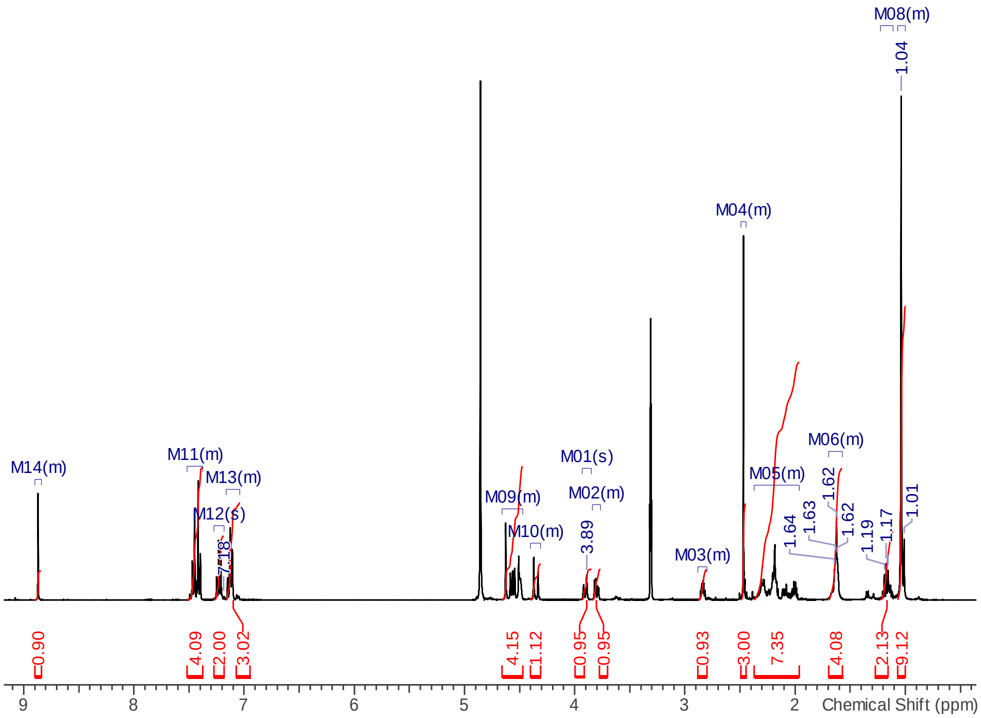
M14(m)
M11(m)
M13(m)
M12(s)
7.18
M09(m)
M10(m)
M01(s)
M02(m)
3.89
M03(m)
M04(m)
M05(m)
1.64
1.63
1.62
M06(m)
1.62
1.19
1.17
1.01
M08(m)
1.04
0.90
4.09
2.00
3.02
4.15
1.12
0.95
0.95
0.93
3.00
7.35
4.08
2.13
9.12
9
8
7
6
5
4
3
2
Chemical Shift (ppm)

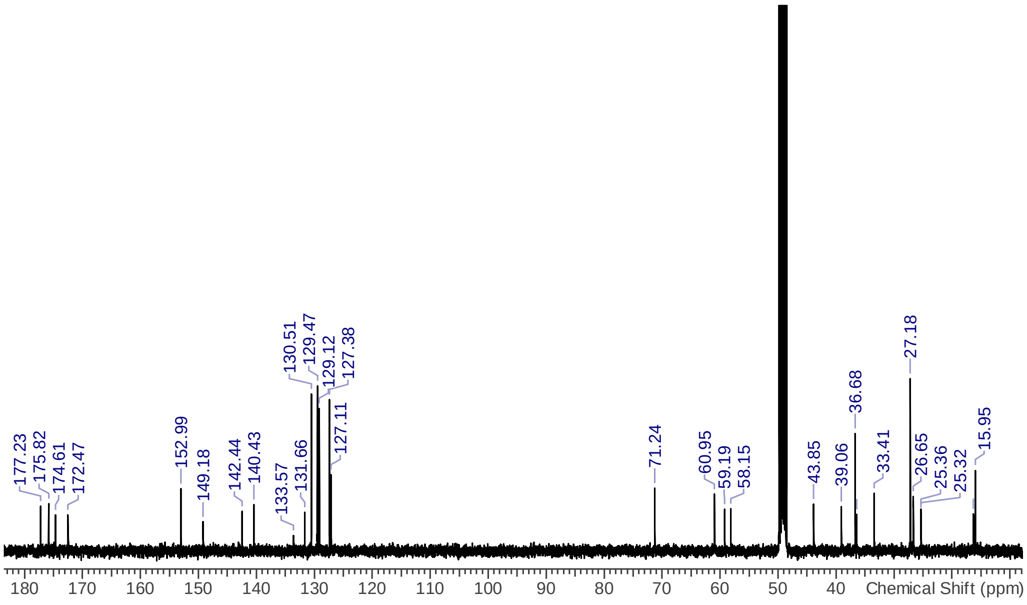
177.23
175.82
174.61
172.47
152.99
149.18
142.44
140.43
133.57
131.66
130.51
129.47
129.12
127.38
127.11
71.24
60.95
59.19
58.15
43.85
39.06
36.68
33.41
27.18
26.65
25.36
25.32
15.95
180
170
160
150
140
130
120
110
100
90
80
70
60
50
40
Chemical Shift (ppm)

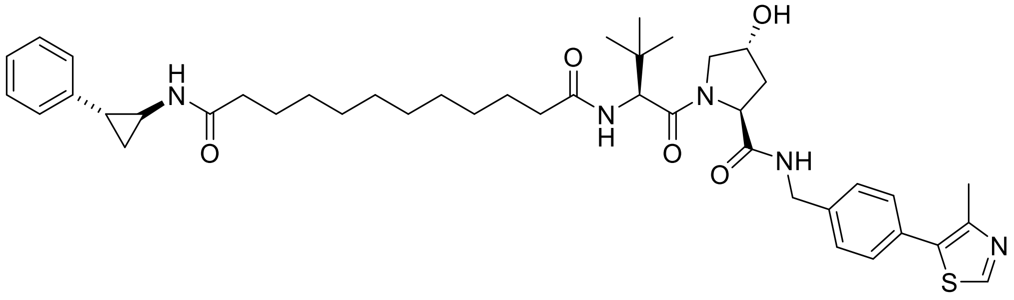

OH
H
N
O
O
N
H
O
N
O
NH
N
S

Compound **39**

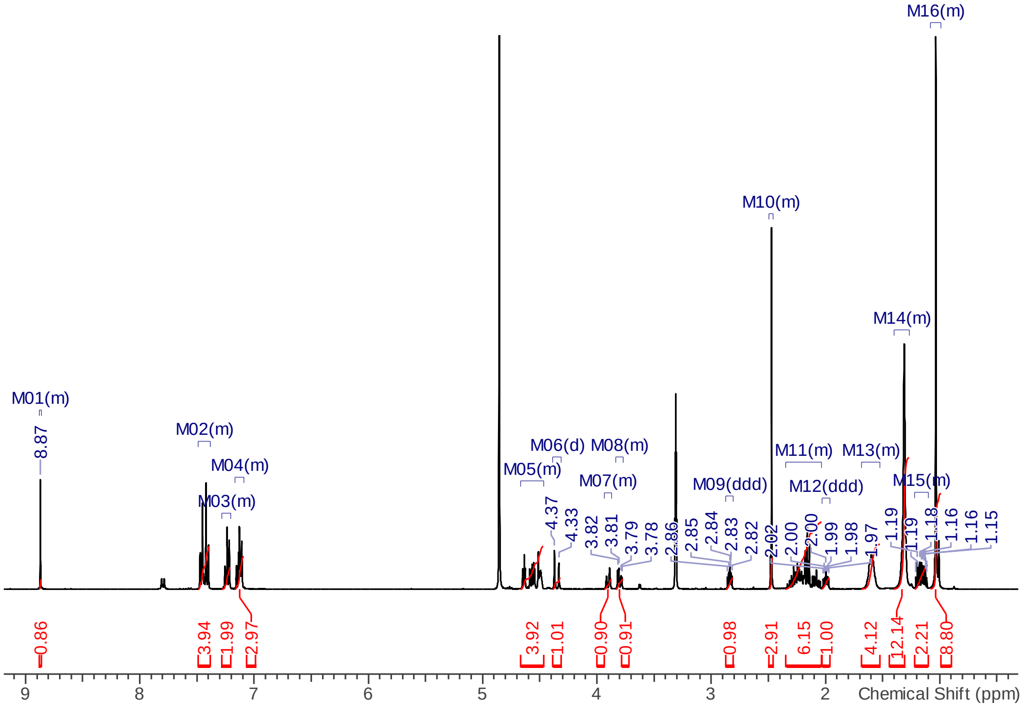

M01(m)
8.87
M02(m)
M03(m)
M04(m)
M05(m)
M06(d)
M07(m)
M08(m)
M09(ddd)
M10(m)
M11(m)
M12(ddd)
M13(m)
M14(m)
M15(m)
M16(m)
4.37
4.33
3.82
3.81
3.79
3.78
2.86
2.85
2.84
2.83
2.82
2.02
2.00
2.00
1.99
1.98
1.97
1.19
1.19
1.18
1.16
1.16
1.15
0.86
3.94
1.99
2.97
3.92
1.01
0.90
0.91
0.98
2.91
6.15
1.00
4.12
12.14
2.21
8.80
9
8
7
6
5
4
3
2
Chemical Shift (ppm)

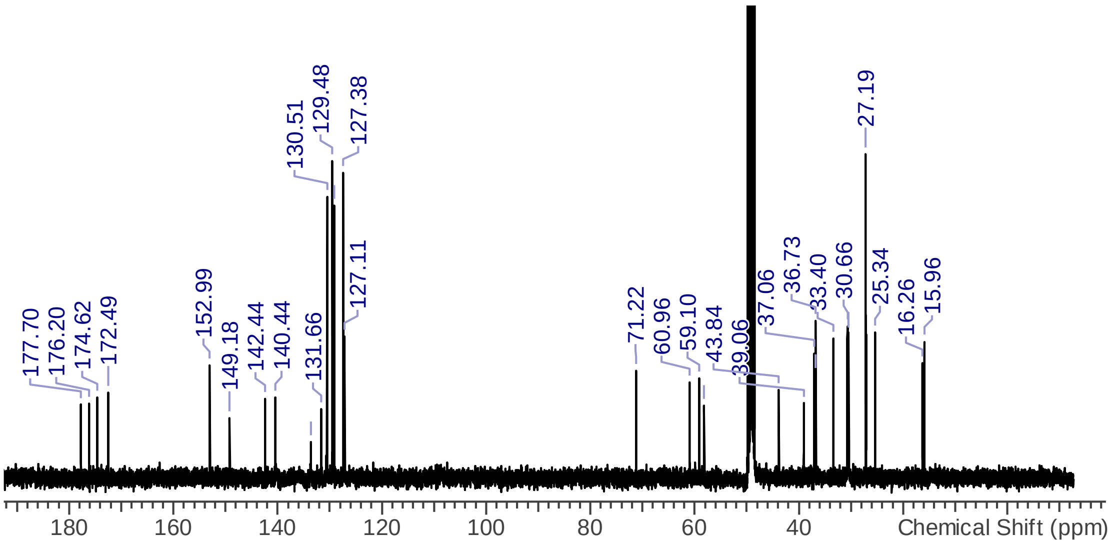

177.70
176.20
174.62
172.49
152.99
149.18
142.44
140.44
131.66
130.51
129.48
127.38
127.11
71.22
60.96
59.10
43.84
39.06
37.06
36.73
33.40
30.66
27.19
25.34
16.26
15.96
180
160
140
120
100
80
60
40
Chemical Shift (ppm)

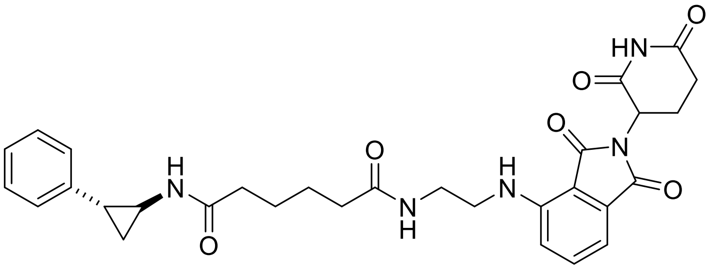

Compound **40**

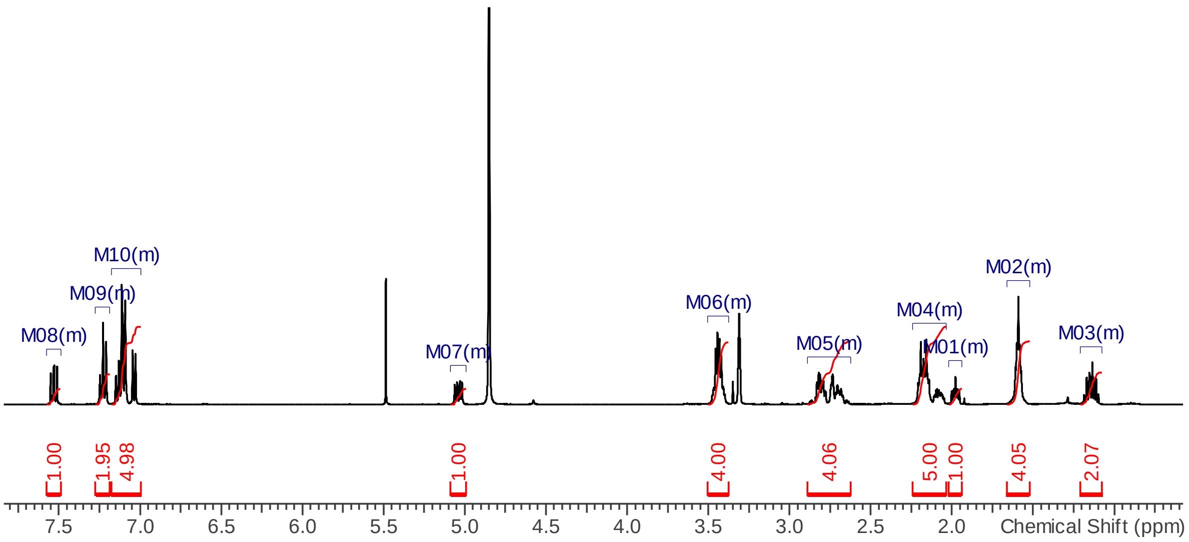
M08(m)
M09(m)
M10(m)
M07(m)
M06(m)
M05(m)
M04(m)
M01(m)
M02(m)
M03(m)
1.00
1.95
4.98
1.00
4.00
4.06
5.00
1.00
4.05
2.07
7.5
7.0
6.5
6.0
5.5
5.0
4.5
4.0
3.5
3.0
2.5
2.0
Chemical Shift (ppm)

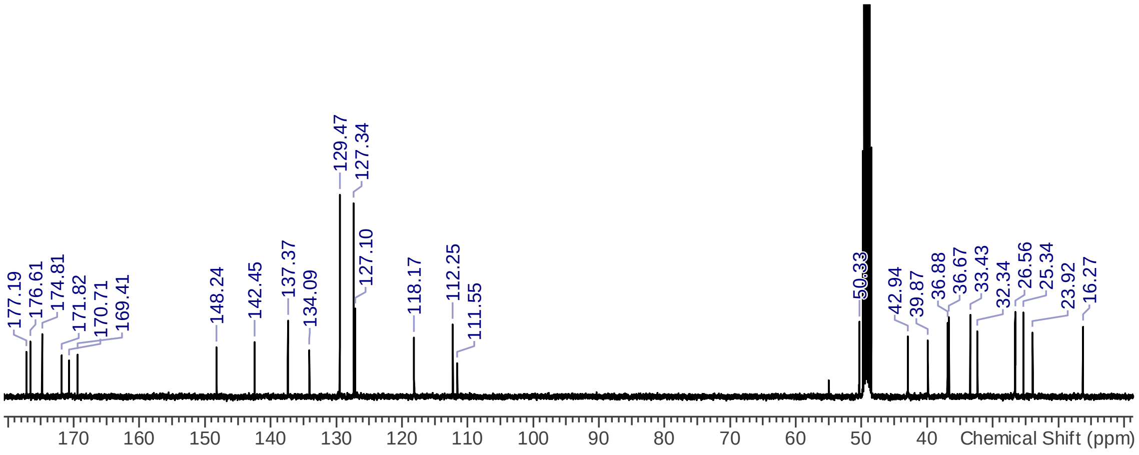
177.19
176.61
174.81
171.82
170.71
169.41
148.24
142.45
137.37
134.09
129.47
127.34
127.10
118.17
112.25
111.55
50.33
42.94
39.87
36.88
36.67
33.43
32.34
26.56
25.34
23.92
16.27
170
160
150
140
130
120
110
100
90
80
70
60
50
40
Chemical Shift (ppm)

Compound **41**

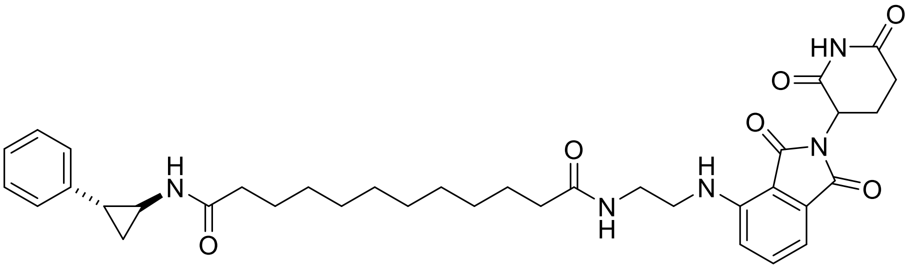

HN
O
O
O
N
O
H
N
O
O
N
H
H
N

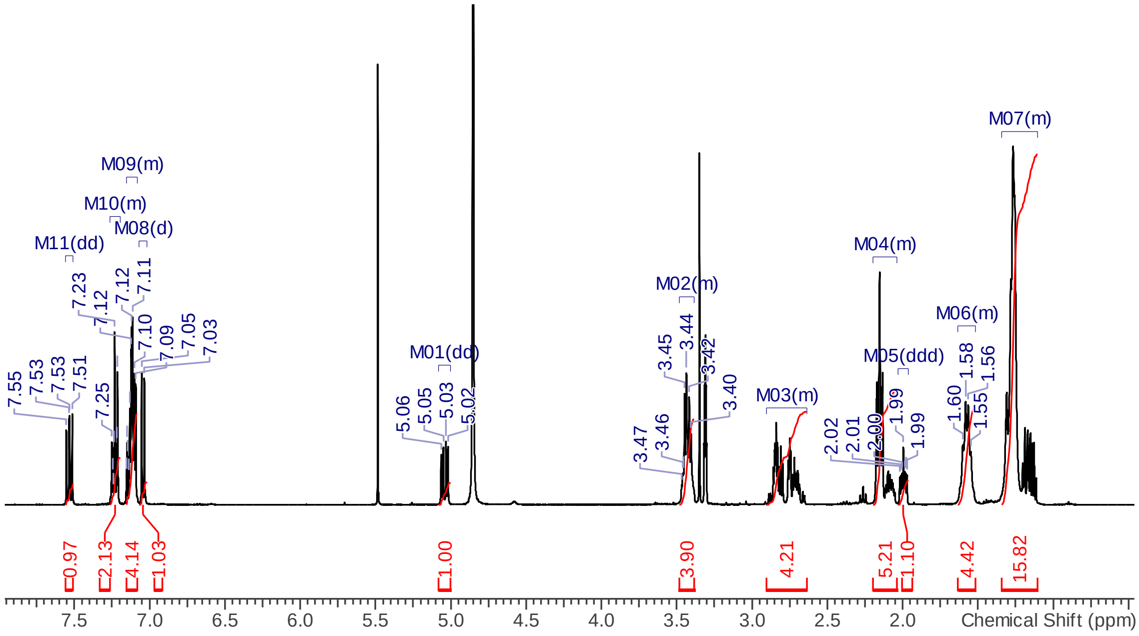

M11(dd)
M10(m)
M09(m)
M08(d)
M01(dd)
M02(m)
M03(m)
M04(m)
M05(ddd)
M06(m)
M07(m)
7.55
7.53
7.53
7.51
7.25
7.23
7.12
7.12
7.11
7.10
7.09
7.05
7.03
5.06
5.05
5.03
5.02
3.47
3.46
3.45
3.44
3.42
3.40
2.02
2.01
2.00
1.99
1.99
1.60
1.58
1.56
1.55
0.97
2.13
4.14
1.03
1.00
3.90
4.21
5.21
1.10
4.42
15.82
7.5
7.0
6.5
6.0
5.5
5.0
4.5
4.0
3.5
3.0
2.5
2.0
Chemical Shift (ppm)

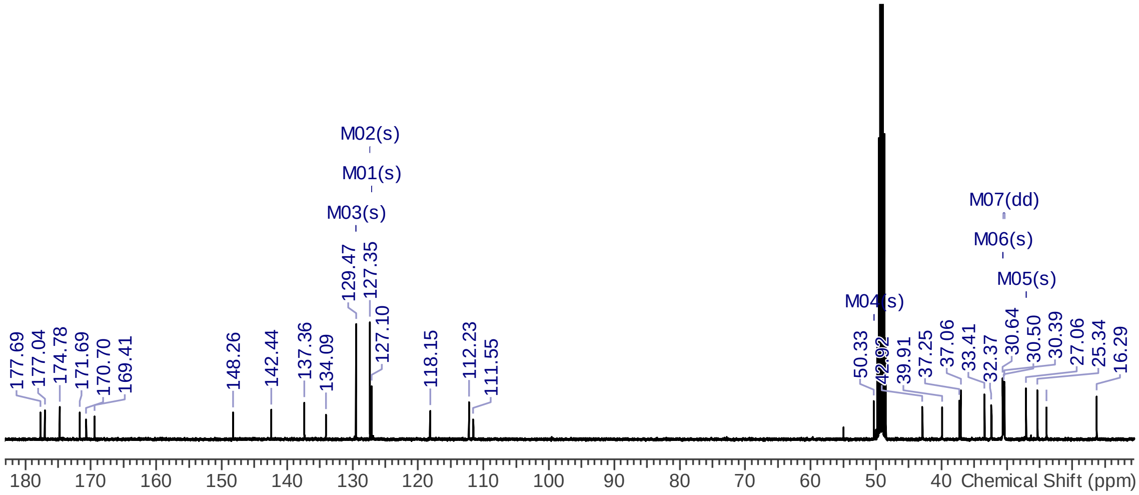

M02(s)
M01(s)
M03(s)
M04(s)
M07(dd)
M06(s)
M05(s)
177.69
177.04
174.78
171.69
170.70
169.41
148.26
142.44
137.36
134.09
129.47
127.35
127.10
118.15
112.23
111.55
50.33
42.92
39.91
37.25
37.06
33.41
32.37
30.64
30.50
30.39
27.06
25.34
16.29
180
170
160
150
140
130
120
110
100
90
80
70
60
50
40
Chemical Shift (ppm)

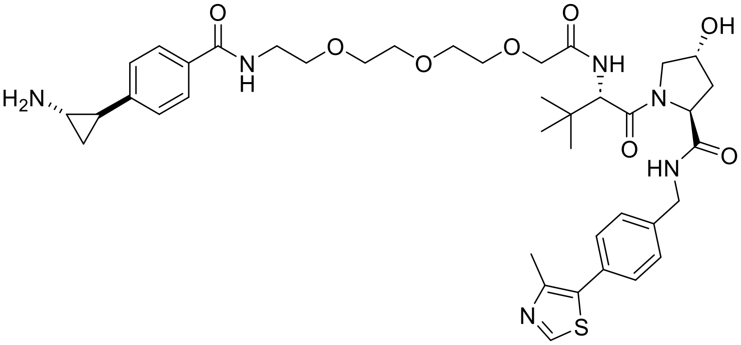

Compound **49**

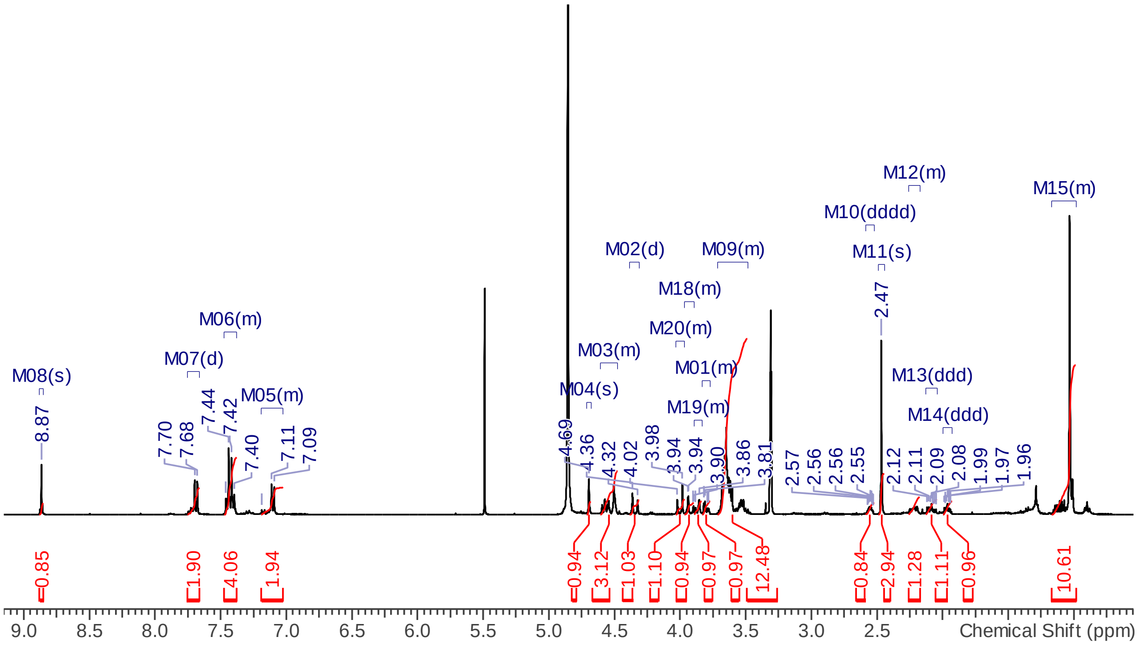
M08(s)
M07(d)
M06(m)
M05(m)
M04(s)
M03(m)
M02(d)
M18(m)
M20(m)
M01(m)
M19(m)
M09(m)
M10(dddd)
M11(s)
M12(m)
M13(ddd)
M14(ddd)
M15(m)
8.87
7.70
7.68
7.44
7.42
7.40
7.11
7.09
4.69
4.36
4.32
4.02
3.98
3.94
3.94
3.90
3.86
3.81
2.57
2.56
2.56
2.55
2.47
2.12
2.11
2.09
2.08
1.99
1.97
1.96
0.85
1.90
4.06
1.94
0.94
3.12
1.03
1.10
0.94
0.97
0.97
12.48
0.84
2.94
1.28
1.11
0.96
10.61
9.0
8.5
8.0
7.5
7.0
6.5
6.0
5.5
5.0
4.5
4.0
3.5
3.0
2.5
Chemical Shift (ppm)

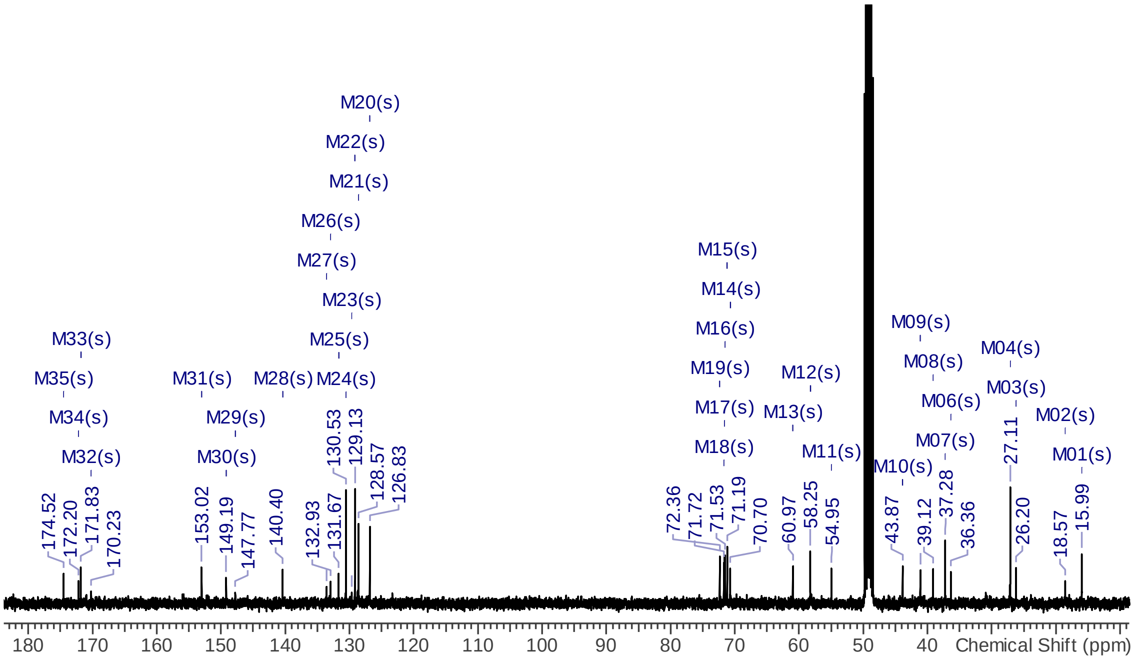
M33(s)
M35(s)
M34(s)
M32(s)
M31(s)
M29(s)
M30(s)
M28(s)
M24(s)
M25(s)
M23(s)
M27(s)
M26(s)
M21(s)
M22(s)
M20(s)
M15(s)
M14(s)
M16(s)
M19(s)
M17(s)
M18(s)
M12(s)
M13(s)
M11(s)
M10(s)
M09(s)
M08(s)
M06(s)
M07(s)
M04(s)
M03(s)
M02(s)
M01(s)
174.52
172.20
171.83
170.23
153.02
149.19
147.77
140.40
132.93
131.67
130.53
129.13
128.57
126.83
72.36
71.72
71.53
71.19
70.70
60.97
58.25
54.95
43.87
39.12
37.28
36.36
27.11
26.20
18.57
15.99
180
170
160
150
140
130
120
110
100
90
80
70
60
50
40
Chemical Shift (ppm)

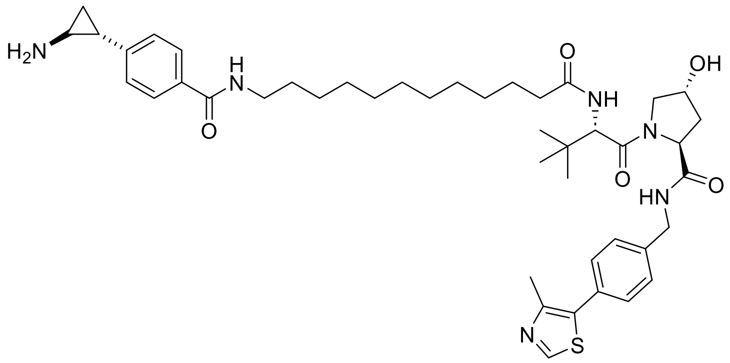

H2N
H
N
O
O
NH
OH
N
O
O
HN
N
S

Compound **52**

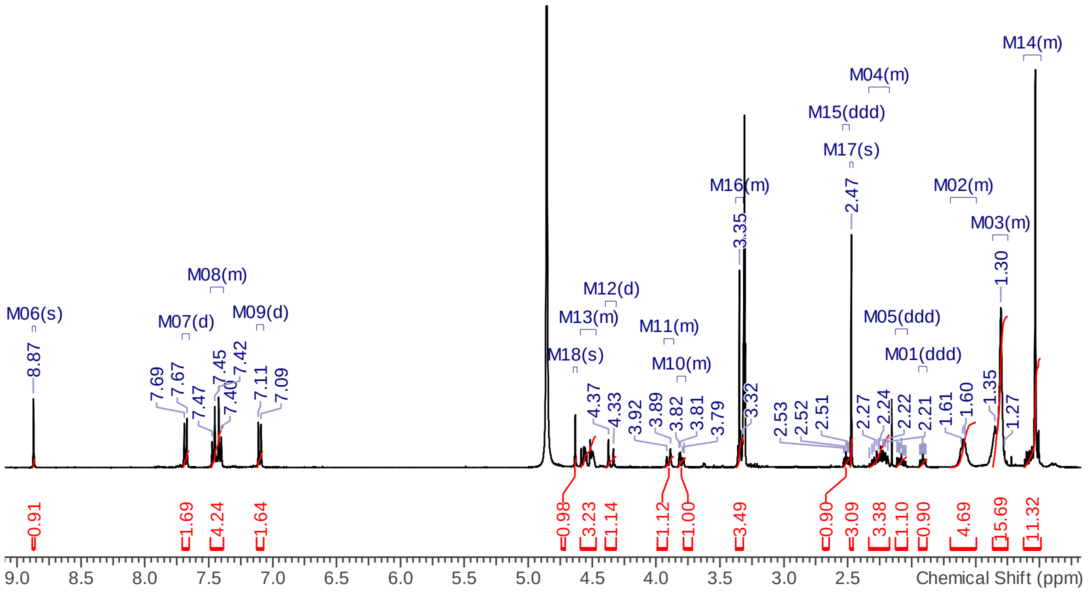

M06(s)
M07(d)
M08(m)
M09(d)
M12(d)
M13(m)
M18(s)
M11(m)
M10(m)
M16(m)
M15(ddd)
M04(m)
M17(s)
M05(ddd)
M01(ddd)
M02(m)
M03(m)
M14(m)
8.87
7.69
7.67
7.47
7.45
7.42
7.40
7.11
7.09
4.37
4.33
3.92
3.89
3.82
3.81
3.79
3.35
3.32
2.53
2.52
2.51
2.47
2.27
2.24
2.22
2.21
1.61
1.60
1.35
1.30
1.27
0.91
1.69
4.24
1.64
0.98
3.23
1.14
1.12
1.00
3.49
0.90
3.09
3.38
1.10
0.90
4.69
15.69
11.32
9.0
8.5
8.0
7.5
7.0
6.5
6.0
5.5
5.0
4.5
4.0
3.5
3.0
2.5
Chemical Shift (ppm)

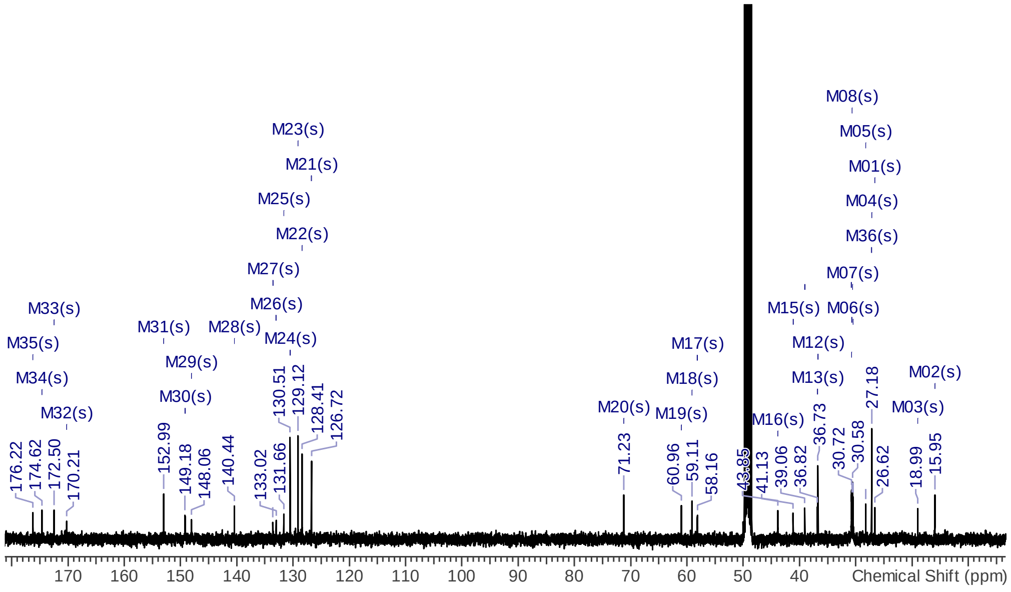

M33(s)
M35(s)
M34(s)
M32(s)
M31(s)
M29(s)
M30(s)
M28(s)
M23(s)
M21(s)
M25(s)
M22(s)
M27(s)
M26(s)
M24(s)
M20(s)
M17(s)
M18(s)
M19(s)
M16(s)
M08(s)
M05(s)
M01(s)
M04(s)
M36(s)
M07(s)
M15(s)
M06(s)
M12(s)
M13(s)
M02(s)
M03(s)
176.22
174.62
172.50
170.21
152.99
149.18
148.06
140.44
133.02
131.66
130.51
129.12
128.41
126.72
71.23
60.96
59.11
58.16
43.85
41.13
39.06
36.82
36.73
30.72
30.58
27.18
26.62
18.99
15.95
170
160
150
140
130
120
110
100
90
80
70
60
50
40
Chemical Shift (ppm)

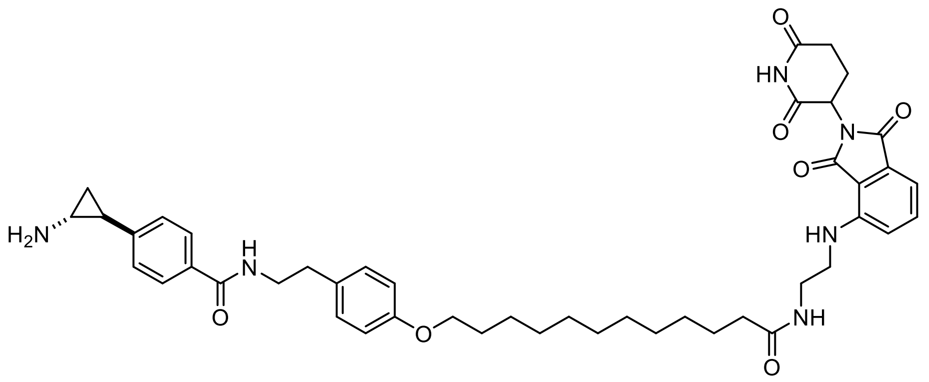

Compound **54**

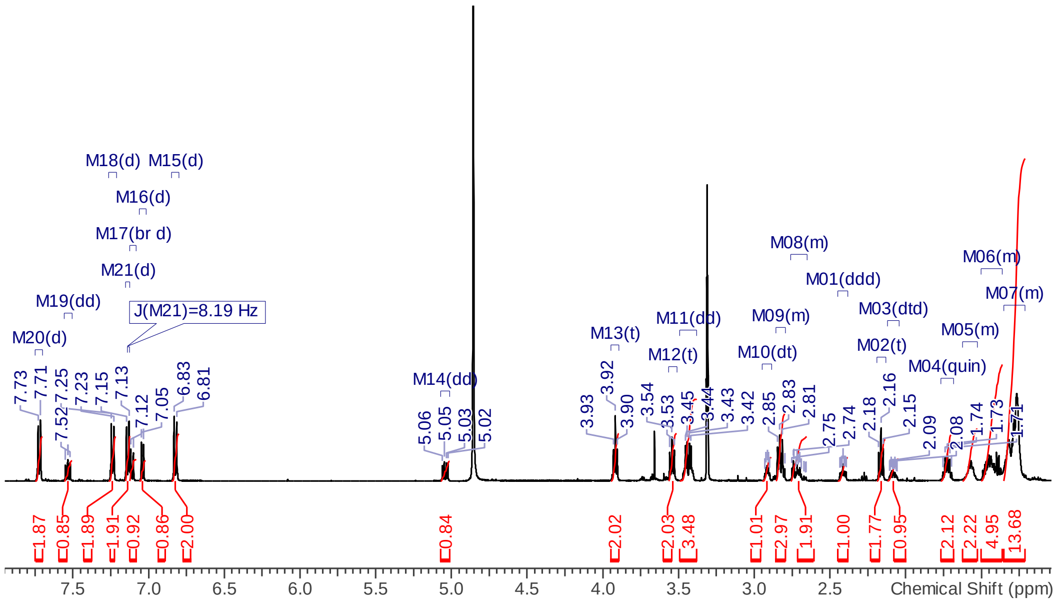

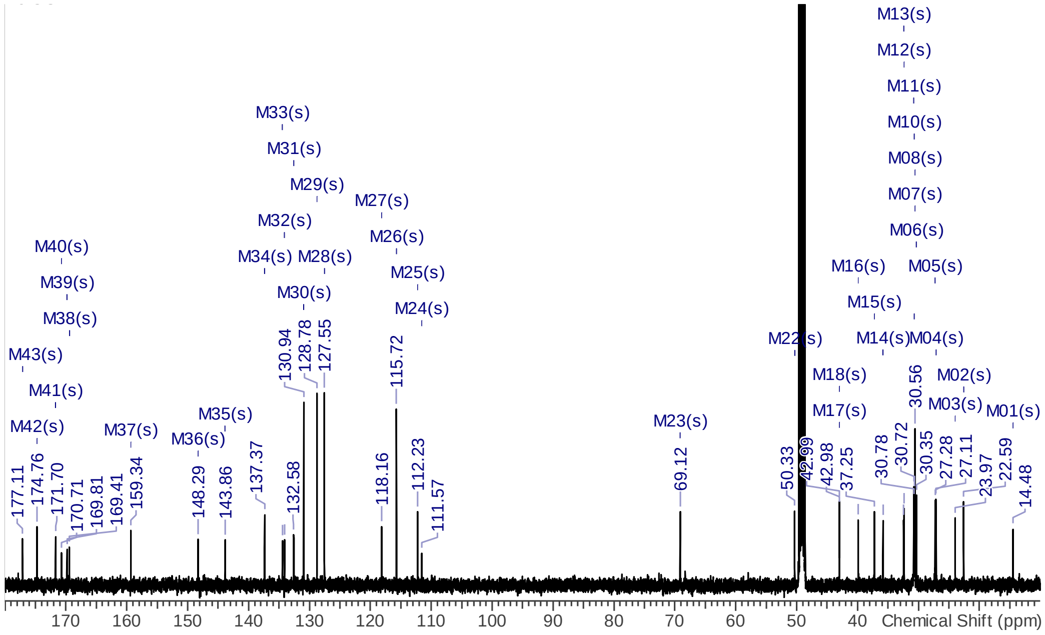

Compound **55**

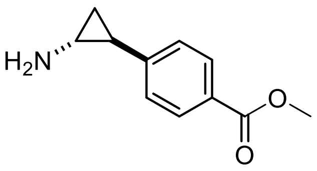
H2N
O
O

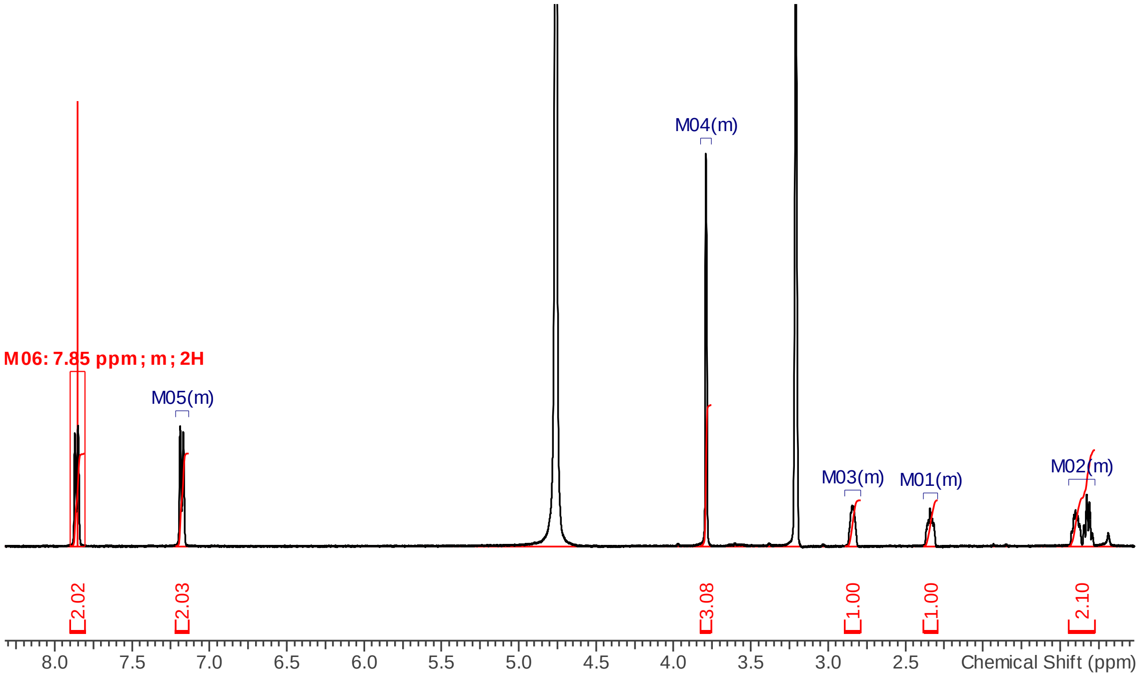
M06: 7.85 ppm ; m ; 2H
M05(m)
M04(m)
M03(m)
M01(m)
M02(m)
2.02
2.03
3.08
1.00
1.00
2.10
8.0 7.5 7.0 6.5 6.0 5.5 5.0 4.5 4.0 3.5 3.0 2.5
Chemical Shift (ppm)

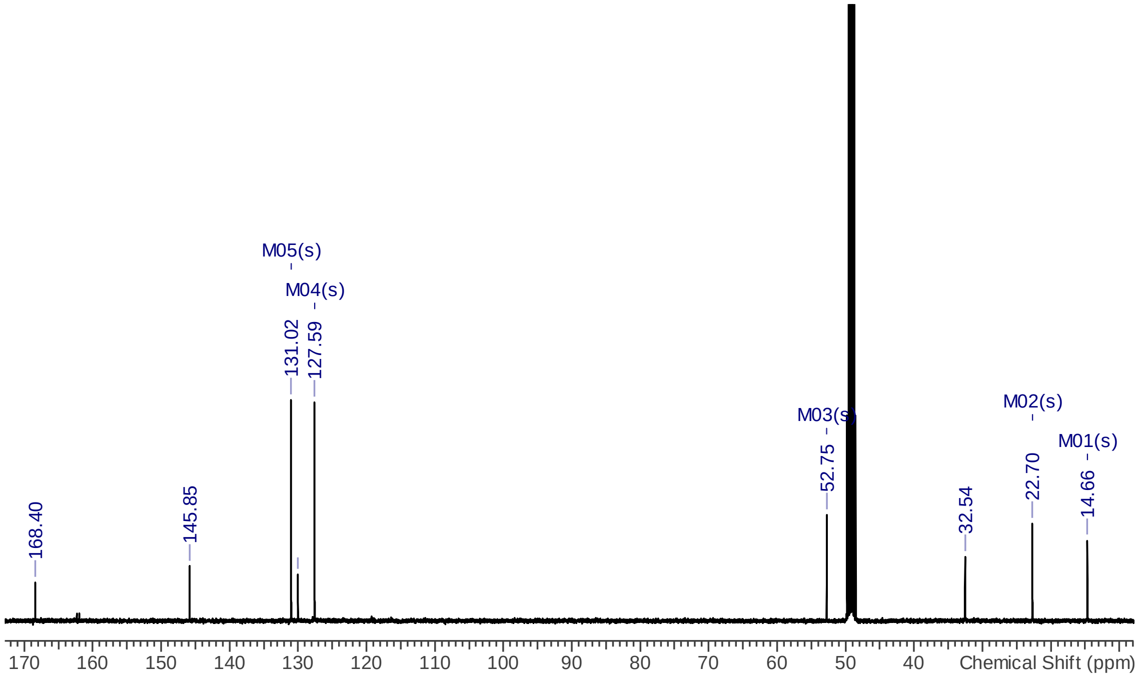
M05(s)
M04(s)
M03(s)
M02(s)
M01(s)
168.40
145.85
131.02
127.59
52.75
32.54
22.70
14.66
170 160 150 140 130 120 110 100 90 80 70 60 50 40
Chemical Shift (ppm)
